# Large-scale sequencing of flatfish genomes provides insights into the polyphyletic origin of their specialized body plan

**DOI:** 10.1038/s41588-021-00836-9

**Published:** 2021-04-19

**Authors:** Zhenming Lü, Li Gong, Yandong Ren, Yongjiu Chen, Zhongkai Wang, Liqin Liu, Haorong Li, Xianqing Chen, Zhenzhu Li, Hairong Luo, Hui Jiang, Yan Zeng, Yifan Wang, Kun Wang, Chen Zhang, Haifeng Jiang, Wenting Wan, Yanli Qin, Jianshe Zhang, Liang Zhu, Wei Shi, Shunping He, Bingyu Mao, Wen Wang, Xiaoyu Kong, Yongxin Li

**Affiliations:** 1 National Engineering Laboratory of Marine Germplasm Resources Exploration and Utilization, Zhejiang Ocean University, Zhoushan, China; 2 School of Ecology and Environment, Northwestern Polytechnical University, Xi’an, China; 3 CAS Key Laboratory of Tropical Marine Bio-resources and Ecology, Guangdong Provincial Key Laboratory of Applied Marine Biology, South China Sea Institute of Oceanology, Chinese Academy of Sciences, Guangzhou, China; 4 State Key Laboratory of Genetic Resources and Evolution, Kunming Institute of Zoology, Chinese Academy of Sciences, Kunming, China; 5 Key Laboratory of Aquatic Biodiversity and Conservation, Institute of Hydrobiology, Chinese Academy of Sciences, Wuhan, China; 6 Center for Excellence in Animal Evolution and Genetics, Chinese Academy of Sciences, Kunming, China

**Keywords:** Genetics, Zoology

## Abstract

The evolutionary and genetic origins of the specialized body plan of flatfish are largely unclear. We analyzed the genomes of 11 flatfish species representing 9 of the 14 Pleuronectiforme families and conclude that Pleuronectoidei and Psettodoidei do not form a monophyletic group, suggesting independent origins from different percoid ancestors. Genomic and transcriptomic data indicate that genes related to WNT and retinoic acid pathways, hampered musculature and reduced lipids might have functioned in the evolution of the specialized body plan of Pleuronectoidei. Evolution of Psettodoidei involved similar but not identical genes. Our work provides valuable resources and insights for understanding the genetic origins of the unusual body plan of flatfishes.

## Abstract (web summary)

Analysis of 11 flatfish genomes indicates that Pleuronectoidei and Psettodoidei are not a monophyletic group. Genomic and transcriptomic data suggest that the WNT and RA pathways played a role in the evolution of the specialized body of flatfish.

## Main

The colonization of the seafloor is one of the most important events in evolutionary history, which led to an explosive radiation and large-scale morphological diversification of marine phyla^1,2^. Flatfishes are one of the most successful groups of seafloor colonizers and have evolved a specialized morphology that is unique in teleosts. Such morphological innovations include a flat and thin body plan that facilitates embedding into substrates^3^, an asymmetrical body axis, mostly represented by one eye migrating to the contralateral side of the skull for gaining binocular vision, which ensures improved success of preying^4^, and modified median and paired fins that coordinate together to enable flexible over-substrate ‘fin-feet’ walking^5,6^. The body plan exhibited by flatfishes reflects morphological trade-offs to facilitate embedding, predation and maneuvering behaviors adapted to their over-substrate dwelling lifestyle^4,6^. However, the genetic basis of such morphological adaptations in flatfishes has remained largely unknown since the time of Darwin^7,8^.

Some progress has been made concerning the evolutionary origin and the morphological adaptations of flatfishes in recent years. Current views support the origin of flatfishes among basally diverging percoids^9–11^. Despite this progress, there is still disagreement regarding when and how flatfishes diverged from their ancestors. An unanswered question is whether the flatfishes (particularly Pleuronectoidei and Psettodoidei, the only two suborders of Pleuronectiformes) share a monophyletic origin^9,12–14^. This has been difficult to address due to limitations in providing a solid evolutionary framework for understanding the genetic basis of the morphological adaptations of flatfishes; largely because the supposed polyphyletic origin may predict differed genetic mechanisms for their morphological adaptations. The early exploration of the genetic origin of the specialized morphology of flatfishes was started by Inui et al.^15^, and continued by Hashimoto et al.^16,17^ and Suzuki et al.^18^, but the results varied from either a NODAL or thyroid hormone (TH) regulation of their asymmetrical body plan. Shao et al.^19^ were the first to elaborate on this topic by applying a genomic framework and providing evidence for retinoic acid (RA) and TH involvement in the regulation of body plan asymmetry of flatfishes. However, all these studies mainly focused on the asymmetric body plan in flatfishes under the framework of only one or two flatfish species, while the genetic basis of a wider spectrum of morphological adaptations (for example, body-plan flatness, body and eye asymmetry and fin modification) in the whole flatfish group remains to be explored from a systematic evolutionary perspective.

In the present study, we assembled genomes of eight species de novo (*Trinectes maculatus*, *Chascanopsetta lugubris*, *Brachirus orientalis*, *Paraplagusia blochii*, *Colistium nudipinnis*, *Pseudorhombus dupliocellatus*, *Platichthys stellatus* and *Psettodes erumei*) representing most of the major extant clades (8 of 14 families, including the sole family in Psettodoidei and 7 families in Pleuronectoidei) of Pleuronectiformes and two closely related species of Perciformes with regular body plans (*Toxotes chatareus* and *Polydactylus sextarius*). Combined with three previously published genomes of flatfish species, which added one more family, and 80 transcriptomes (including 72 from three tissues of *Paralichthys olivaceus*; 4 from two tissues of *Platichthys stellatus*; 2 from two tissues of *Toxotes chatareus* and 2 from two tissues of *Polydactylus sextarius*) that we generated, we systematically studied: (1) the phylogeny of flatfishes, which provides an evolutionary framework for better understanding the genetic adaptation of flatfishes; and (2) genes that experienced significant alterations, to gain insights into the genetic basis underlying the unusual body plan of flatfishes.

## Results

### Genome assembly and annotation

**Fig. 1:**
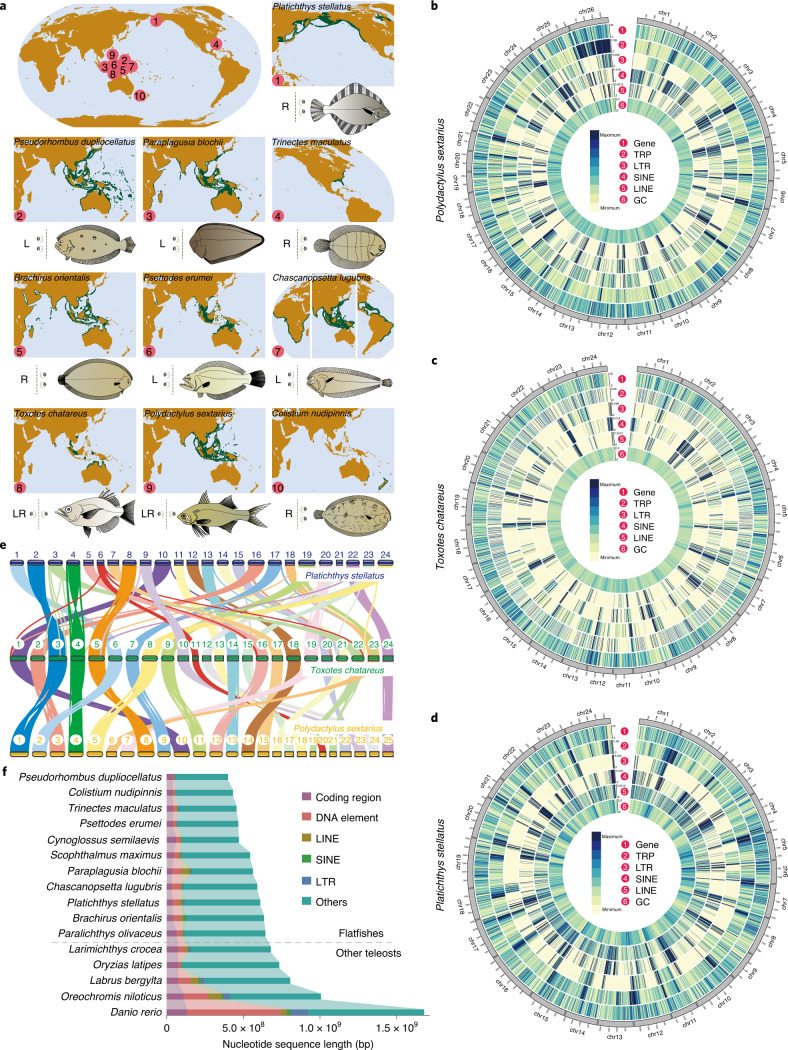
Genome assembly and gene annotation of ten species. **a**, Geographical distribution of the species sequenced in this study. The dark green areas on the maps represent the global distribution regions of each species, which were acquired from the open source FishBase database (www.fishbase.se). The L, R and LR represent sequenced species that have left-side eyes, right-side eyes or one eye on each side of the body, respectively. **b**–**d**, Circos plot of distribution of the genomic elements in the species *Polydactylus sextarius* (**b**), *Toxotes chatareus* (**c**) and *Platichthys stellatus* (**d**). From outer to inner ring are the distributions of protein-coding genes, tandem repeats (TRP), long terminal repeats (LTR), short interspersed nuclear elements (SINE), long interspersed nuclear elements (LINE) and GC content, respectively. **e**, Genomic colinearity of three chromosome-level genomes. The number in the circles represents the chromosome identity for each species. **f**, Genome size statistics of these species. Diagram indicates the size of each type of element, including the coding regions, DNA elements, LINE, SINE, LTR and other genomic regions, in each species.

**Extended Data Fig. 1:**
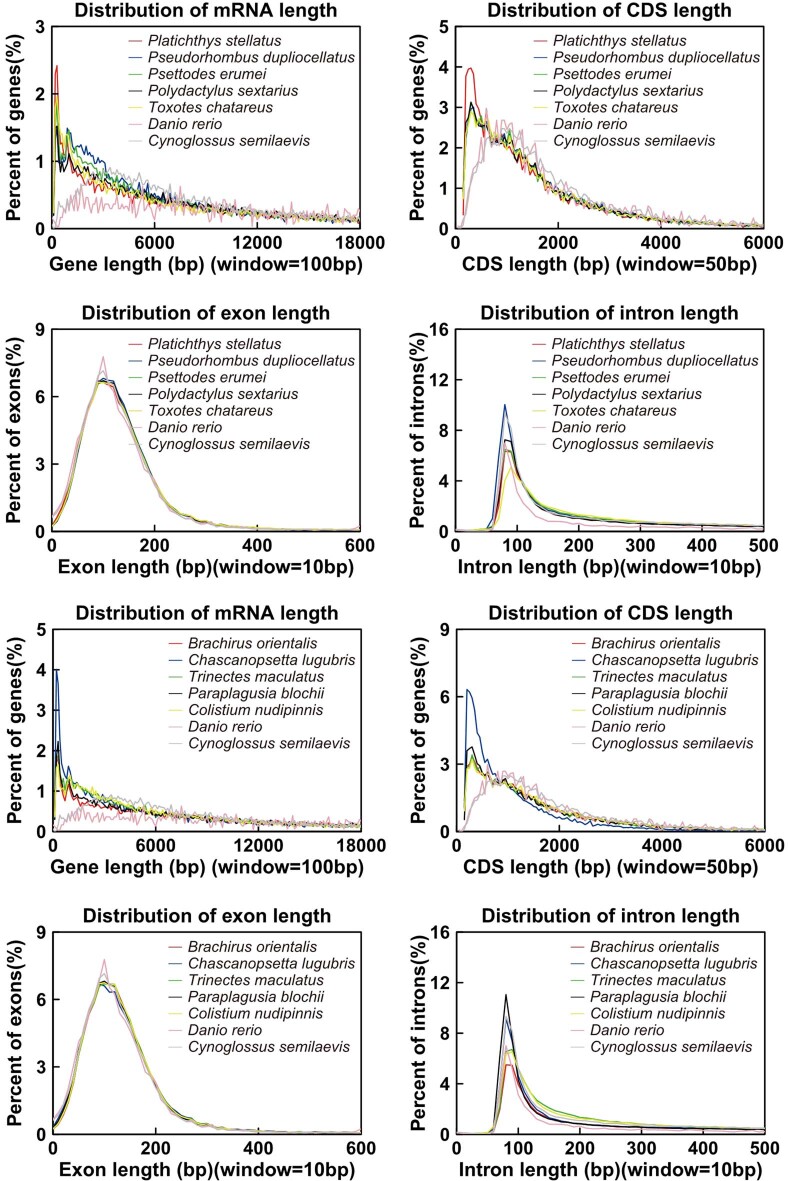
Comparison of the annotated coding genes in the species analyzed in this study with those annotated in previously published species. The x-axis shows the length distribution of the mRNA, CDS, exon, and intron sequences in each species, and the y-axis shows the corresponding ratios of each type of sequences in genome for a particular length.

Using whole-genome sequencing strategies, we generated more than two terabytes (Tb) of sequencing data (Supplementary Tables 1–13 and Supplementary Notes 1–5) and de novo-assembled genomes of the ten species indicated above (Fig. 1a, Supplementary Tables 14–23 and Supplementary Notes 6 and 7). Among them, three species with controversial phylogenetic status, including *Psettodes erumei* (Pleuronectiformes), *Toxotes chatareus* (Perciformes) and *Polydactylus sextarius* (Perciformes), were sequenced using a Nanopore platform (Supplementary Tables 1 and 12 and Supplementary Notes 1, 3 and 4). Hi-C data analysis supported the generation of chromosome-level genome assemblies for three species: *Platichthys stellatus*, *Toxotes chatareus* and *Polydactylus sextarius* (Fig. 1b–d, Supplementary Tables 24–29, Supplementary Figs. 1–3 and Supplementary Note 8). All ten assembled genomes possess high continuity and accuracy as indicated by the N50 length (64.40 kilobases (kb)–25.10 megabases (Mb); Supplementary Tables 14–26 and Supplementary Notes 6–8), genome integrity (Supplementary Table 30, Supplementary Fig. 4 and Supplementary Note 9), BUSCO scores (93.6–99.1%; Supplementary Tables 31–40 and Supplementary Note 10), read mapping ratios (94.64–99.87%; Supplementary Table 41 and Supplementary Note 10) and transcript mapping ratios (95.06–99.32%; Supplementary Tables 42–47 and Supplementary Note 10). The quality of the chromosome-level assemblies was also demonstrated by the good genome synteny (Fig. 1e, Supplementary Figs. 5–9 and Supplementary Note 11). The assembled genome sizes range from 399.64 Mb (*Pseudorhombus dupliocellatus*) to 643.91 Mb (*Paralichthys olivaceus*) (Fig. 1f, Supplementary Tables 14–30 and Supplementary Notes 6–9). After masking repetitive sequences (Supplementary Tables 48–68, Supplementary Fig. 10 and Supplementary Notes 12 and 13), these genomes were predicted to contain ~20,000 protein-coding genes (Supplementary Tables 69–79 and Supplementary Notes 14 and 15), which share similar gene structures to the published genomes (Extended Data Fig. 1 and Supplementary Note 14).

### Polyphyletic origin of flatfishes

**Fig. 2:**
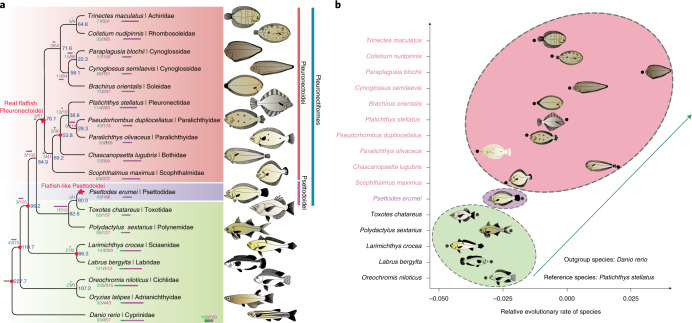
Polyphyletic origin and fast genome evolution of flatfishes. **a**, Phylogenetic relationship and divergence time among the flatfish species. The number in each node represents the divergence time among species and the red circle indicates the fossil record used for calibration in the node. The numbers with slashes, as well as the bars, represent the expanded and contracted gene families in this node, respectively. The silhouette image of each species was drawn according to their morphology using Adobe Illustrator software. **b**, Relative evolutionary rates of the flatfish species. Zebrafish was used as the outgroup and *Platichthys stellatus* as the reference species. The arrow represents the trend of the relative evolutionary rates. The colored ovals represent different fish groups that showed contrasted relative evolutionary rates.

By combining our ten de novo-assembled genomes with eight published genome sequences from teleost species of *Cynoglossus semilaevis*, *Paralichthys* *olivaceus*, *Scophthalmus maximus*, *Larimichthys crocea*, *Labrus bergylta*, *Oreochromis niloticus*, *Oryzias latipes* and *Danio rerio* (see Supplementary Note 16), we reconstructed the phylogeny of flatfishes using concatenated sequences of coding sequence (CDS) (codon1 + 2 + 3, GTRGAMMA model; codon1 + 2, GTRGAMMA model) and 4dTV (fourfold degenerate synonymous site, GTRGAMMA model) derived from 1,693 single-copy genes (Supplementary Figs. 11–15 and Supplementary Notes 16 and 17). We further constructed the species tree under the coalescent model^20,21^. Our results consistently show that *Psettodes erumei* of suborder Psettodoidei forms one clade with the two Perciformes species with regular body plan, *Toxotes chatareus* and *Polydactylus sextarius*, and species of suborder Pleuronectoidei form its sister clade (Fig. 2a, Supplementary Figs. 16 and 17 and Supplementary Note 17). The observation that, in both gene trees and species trees, *Psettodes erumei* is clustered with nonflatfish Perciformes rather than with Pleuronectoidei species provides strong support for the independent origins of Pleuronectoidei and Psettodoidei. Alternatively, it is also possible that they had a monophyletic origin but secondarily lost their traits independently, in *Toxotes chatareus* and *Polydactylus sextarius*. However, considering that *Psettodes erumei* has also been observed to show affinity to *Sphyraena argentea*, *Centropomus armatus*, *Coryphaena hippurus*, *Nematistius pectoralis* and many other perciforme species rather than Pleuronectoidei in multiple previous phylogenetic studies^22,23^, this scenario is less likely, because multiple independent losses in many species along a lineage are less likely according to the parsimony principle of evolution^24^. We also analyzed mutations in body-plan-related genes, and conclude that many reverse mutations would need to have arisen if we assume secondary losses in *Toxotes chatareus* and *Polydactylus sextarius* (Supplementary Table 80 and Supplementary Note 17), which is less likely in molecular evolution^25^. Furthermore, reconstruction of ancestral chromosomes for the Pleuronectoidei and Psettodoidei lineages also shows that *Psettodes erumei* shares specific chromosome rearrangements with *Toxotes chatareus* and *Polydactylus sextarius*, rather than with Pleuronectoidei species, further supporting a polyphyletic origin for these two lineages (see Supplementary Note 17). Indeed, the morphological resemblance between Psettodoidei and percoids has long been noticed by several ichthyologists^26–28^, and Psettodoidei were even once regarded as ‘simply an asymmetric percoid’^9,26^. The morphological differentiation of Psettodoidei from Pleuronectoidei includes: (1) lack of skin folds around the eyes^29^; (2) posterior insertion of the dorsal fin^30^; (3) less extensive cranial asymmetry^14^; (4) presence of spinous rays in fins^31^; and (5) larger mouths with specialized teeth^31^. These phenotypical observations, combined with our results, provide strong support for a polyphyletic origin of flatfishes, with Psettodoidei and Pleuronectoidei, respectively, arising from two independent evolutionary events. To capture real evolutionary signals, we therefore split the previously known Pleuronectiformes into ‘real flatfish Pleuronectoidei’ (RFP) and ‘flatfish-like Psettodoidei’ (FLP) lineages in the following analyses.

### Fast evolution in flatfishes

With fossil calibration, we estimated the emergence of RFP and FLP to be approximately 76.1 and 80.0 million years ago (Ma), respectively, in the late Cretaceous (Fig. 2a). Our time estimates are consistent with the calibration in a previous study using multiple nuclear loci^9^, which is earlier than other estimations using mitochondrial or few nuclear loci^12,32^. The late Mesozoic to early Cenozoic period, which includes the Cretaceous, is known as the ‘second age of fishes’, marking the onset of major diversifications and morphological diversification of teleosts^33,34^. Guinot and Cavin^35^ attributed such radiations to the combined effects of exceptionally high seawater temperatures, increasing sea levels and widespread epicontinental seas during the period^36,37^. The period of 80–75 Ma during the late Cretaceous experienced a peak of such global change^37^. We hypothesize that such fast seafloor spreading and the resulting explosion of epicontinental habitat may have facilitated the seafloor colonization and eventual origin of flatfishes. Such a scenario also predicts selection pressure and faster evolutionary rate in RFP and FLP, since they experienced radical habitat transition from water column to seafloor. To test this hypothesis, we calculated the relative evolutionary rates in RFP, FLP and closely related Perciformes species using single-copy orthologous genes. Our results revealed a much higher relative evolutionary rate in RFP than in Perciformes species (Fig. 2b, Supplementary Tables 81 and 82 and Supplementary Note 18). The relative evolutionary rate of FLP is also slightly higher than that for Perciformes species (Fig. 2b and Supplementary Tables 81 and 82), which may explain why they exhibit a ‘simply an asymmetric percoid’ phenotype compared to RFP. The higher relative evolutionary rates in both RFP and FLP indicate the possible selection pressure that they experienced, although other factors, such as limited population size and rapid drift, could not be excluded^38^.

### Genes undergoing significant alterations in flatfishes

**Extended Data Fig. 2:**
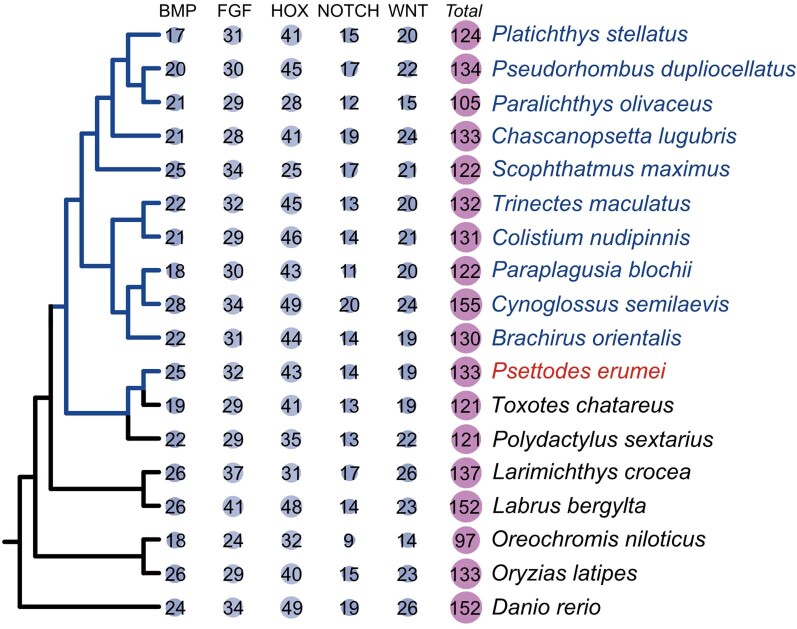
Statistics of gene number in some gene families known to mediate body plan development in each species. The gene number of each specific gene family is shown in blue circle and total gene number known to mediate body plan development is shown in purple circle. The circle sizes are equivalent to the gene number that was observed.

**Extended Data Fig. 3:**
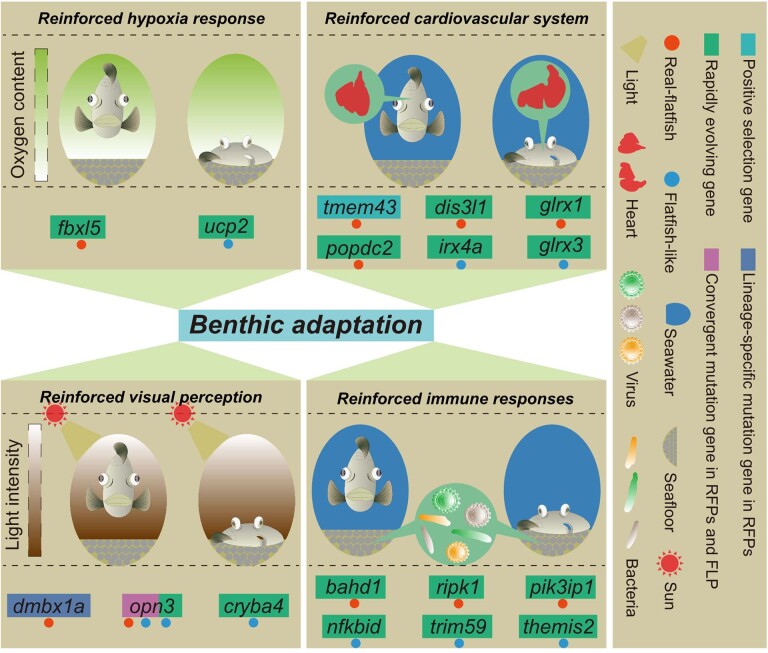
Genes associated with seafloor colonization of flatfishes. The genes associated with certain seafloor colonization adaptation are shown below in the panel. Genes under positive selection, fast evolution, lineage specific mutation or possessing lineage-specific conserved non-coding elements are marked in different colors. The red and blue dots represent real flatfish Pleuronectoidei and flatfish-like Psettodoidei lineages, respectively. The enlarged red diagrams in the panel of ‘reinforced cardiovascular system’ represent heart tissues. The enlarged green diagrams in the panel of ‘reinforced immune responses’ represent bacteria and viruses.

The fast evolution in both RFP and FLP may predict marked changes in their genomes, which had facilitated evolution of their new body plan after seafloor colonization. To test this hypothesis, we performed comprehensive comparisons on genomic elements among RFP, FLP and other nonflatfish outgroup species (*Larimichthys crocea*, *Labrus bergylta*, *Oreochromis niloticus*, *Oryzias latipes* and *Danio rerio*). We first analyzed gene families that changed rapidly in gene number during the evolution process (see Supplementary Note 19), and identified some expanded and contracted gene families (*P* < 0.05) in RFP and FLP, respectively (Supplementary Tables 82–89 and Supplementary Note 19). However, none of these gene families includes those currently known to mediate body plan development (Extended Data Fig. 2 and Supplementary Note 19), such as WNT, RA, BMP, FGF, NOTCH and HOX^39,40^, suggesting involvement of other molecular mechanisms for the unique body plan formation in RFP and FLP. Therefore, we further identified genes undergoing positive selection (PSGs) or rapid evolution (REGs) or containing lineage-specific mutations (LSGs) (Supplementary Figs. 18–23 and Supplementary Notes 20 and 21) or lineage-specific conserved noncoding elements (SCNEs) in RFP and FLP (see Supplementary Note 22), respectively. The enrichment categories of top candidate genes under significant alteration in both RFP and FLP are associated with visual perception (*dmbx1a* (ref. ^41^) and *opn3* (ref. ^42^) in RFP versus *cryba4* (ref. ^43^) and *opn3* (ref. ^42^) in FLP), immune response (*bahd1* (ref. ^44^), *ripk1* (ref. ^45^) and *pik3ip1* (ref. ^46^) in RFP versus *nfkbid* (ref. ^47^), *trim59* (ref. ^48^) and *themis2* (ref. ^49^) in FLP), hypoxia tolerance (*fbxl5* (ref. ^50^) in RFP versus *ucp2* (ref. ^51^) in FLP) and cardiac function (*tmem43* (ref. ^52^), *dis3l1* (ref. ^53^), *popdc2* (ref. ^54^) and *glrx1* (ref. ^55^) in RFP versus *irx4a* (ref. ^56^) and *glrx*3 *(ref.* ^57^) in FLP) (Supplementary Tables 90–97, Extended Data Fig. 3 and Supplementary Notes 20 and 21), possibly suggesting a similar remodeling of their visual, immune, respiratory and circulatory systems in benthic adaptation to seafloor colonization (Extended Data Fig. 3 and Supplementary Note 21). Among them, cardiovascular adaptation is a surprising association with rapid sequence evolution during transition from the water column to benthic colonization. Our results revealed that this process may involve not only a cardiac morphological reorganization resulting from selective pressure on cardiac morphogenesis genes (*popdc2* and *irx4a*)^54,56^, but also cardiac functional remodeling resulting from selective pressure on genes associated with cardiac conducting efficiency (*tmem43* and *popdc2*)^52,54^ and antioxidant capacities (*glrx1* and *glrx3*)^55,57^ (Extended Data Fig. 3, Supplementary Tables 90–97 and Supplementary Notes 20, 21 and 23). Such structural and functional alterations of the cardiovascular system in both RFP and FLP might have contributed to their reinforced cardiac output, which is the highest known among teleosts^58,59^, and the enhanced antioxidant capacity to cope with hypoxia readily encountered during burrowing into the substrate^58^. Enriched categories of the remaining top candidate genes under significant alterations in RFP and FLP were associated with axial patterning, neural patterning, musculoskeletal restructuring, lipid deposition and fin cartilage reorganization (Supplementary Tables 90–99 and Supplementary Notes 20–22), suggesting their roles in new body plan evolution and adaptation after seafloor colonization.

### Genetic changes correlated with the flat body in flatfishes

**Fig. 3:**
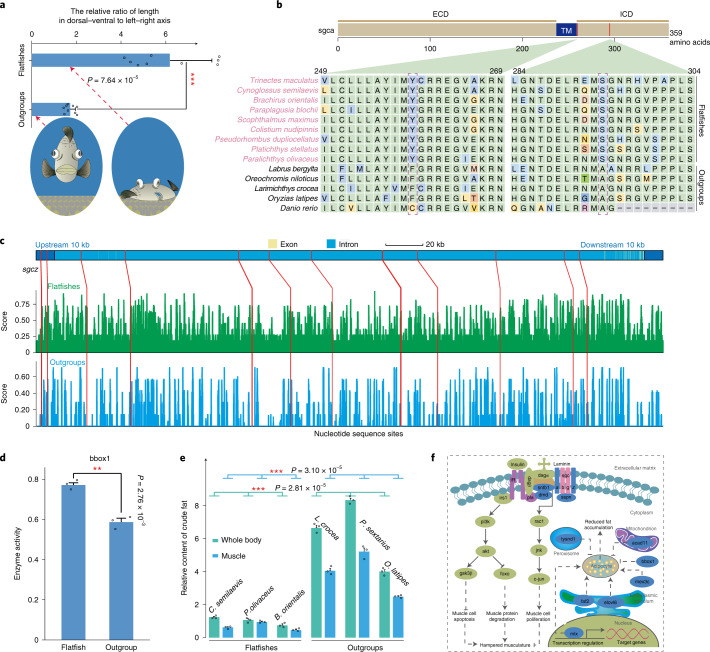
Genetic changes correlated with the flat body plan in flatfishes. **a**, The relative ratio of length in dorsal–ventral to left–right axis. The relative ratio of maximum height of dorsal–ventral axis/maximum length of left–right axis here was used to indicate the degree of body plan flatness of fishes. The ratio was measured in three individuals for each species, and the data are presented as mean values ±s.d. The statistical difference between groups was calculated using Student’s *t*-test (two tails) with *** representing a statistical *P* value <0.001. **b**, Mutations in RFP *sgca* compared to outgroups. ECD, ICD and TM represent the extracellular, intracellular and transmembrane domain, respectively. The fixed substitutions between RFP and outgroups are marked with a dashed box. **c**, SCNEs nearby *sgcz* gene. The *x* axis represents the nucleotide sequence sites across the *sgcz* gene and the *y* axis represents sequence similarity scores. The green and blue columns represent the average sequence similarity score within RFP and outgroups for each site, respectively; the red lines represent the physical locations of the SCNEs across the gene. **d**, The relative catalyzing activity of bbox1 in RFP and outgroups. The experiment was carried out three times and the data are presented as indicated above. **e**, Relative fat content in whole body and muscle tissues in flatfishes and outgroups. The fat content was measured in three individuals for each species, and the data are presented as indicated above. **f**, Hypothetical signaling pathway that may correlate with the body-plan flatness of flatfishes. Proteins marked blue are those encoded by genes that have undergone genetic alterations in RFP. Source data

**Extended Data Fig. 4:**
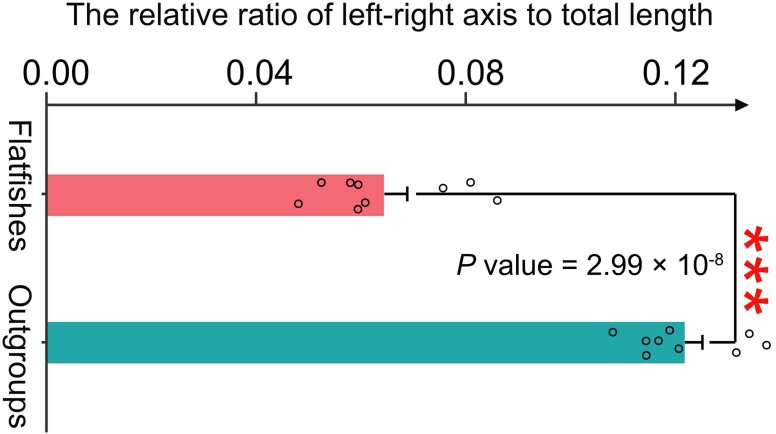
The relative ratio of left-right axis to total length. The relative ratio of maximum length of left-right axis to the total length was used here to indicate the degree of body pan flatness of fishes. The ratio was measured in three individuals for each species and the data are presented as mean values ± SD. The statistical difference between groups was calculated using Student’s *t*-test (two tails) with ‘***’ representing a statistical significance of *P* value < 0.001. Source data

The observed enrichment of genes associated with musculoskeletal restructuring and lipid deposition may reflect their roles in the evolution of body plan flatness after metamorphosis in flatfishes (Fig. 3a, Extended Data Fig. 4, Supplementary Fig. 24 and Supplementary Note 24). Such a phenotype possibly confers a selective advantage on the seafloor, where flatfishes usually hide from their enemies by embedding themselves into a thin layer of substrate, with only the eyes exposed^3,60^. Our comparative genomic analyses, using *Larimichthys crocea*, *Labrus bergylta*, *Oreochromis niloticus*, *Oryzias latipes* and *Danio rerio* as the outgroups, revealed that four genes associated with musculature development have undergone marked alteration in RFP, including the sarcolemma gene *sspn* (PSGs, *P* = 8.43 × 10^−3^), the sarcoglycan genes *sgca* (PSGs, *P* = 3.30 × 10^−3^) and *sgcz* (SCNEs), and the dystrophin gene *dmd* (SCNEs) (Fig. 3b,c, Supplementary Tables 90, 91, 98 and 99 and Supplementary Notes 20 and 22). Unexpectedly, all four of these genes are the core components of the dystrophin–glycoprotein complex (DGC), critical in both mechanical stabilization^61^ and signal-dependent-activated development of muscular tissues^62–64^. Mutations or abnormal expression of these four genes could cause severe muscular dystrophy or substantial reduction in muscle size in vertebrates^65–68^, including zebrafish^69,70^. Among these genes, *sgca* has been the most frequently reported locus that causes the majority of sarcoglycanopathies (one of the severe muscular dystrophies) in humans^71^. Our analysis revealed two RFP-specific missense substitutions in *sgca*, compared to nonflatfish outgroups (Fig. 3b). Both mutations locate within a conserved C-terminal intracellular domain (Fig. 3b), which is critical in the signal-dependent-activated development of muscular tissues. Mutations of this domain frequently cause hampered musculature development and severe muscular dystrophy, such as limb-girdle muscular dystrophy syndrome in humans^72,73^. Such alterations in *sgca* may change the signal-dependent-activation process of muscular development in RFP and thus may have implications in their thinner musculature and flat phenotype.

In addition to musculature, three genes related to lipid metabolism, including *bbox1* (REGs, *P* = 5.01 × 10^−4^), *mex3c* (REGs, *P* = 4.20 × 10^−2^) and *mlx* (REGs, *P* = 2.13 × 10^−2^) also underwent marked changes in RFP (Supplementary Tables 90 and 91 and Supplementary Note 20). These three genes encode either enzymes^74^ or metabolic signals^75,76^ essential for adipogenesis and fat accumulation in vertebrates. Mutations or abnormal expressions of *mex3c* and *mlx* can result in reduced adiposity and lean phenotype in both mouse^75^ and fruit fly^76^. Genetic disruption of *bbox1* leads to a modified serum carnitine level and less fat accumulation in mice^74^. Our in vitro enzyme catalytic activity assay further shows that the RFP-specific bbox1 has significantly higher (*P* = 2.76 × 10^−3^) catalytic activity transforming γ-butyrobetaine into l-carnitine (Fig. 3d, Supplementary Fig. 18 and Supplementary Note 25), a molecule critical for fat oxidization and hence fat accumulation^74^. The increased catalytic activity of bbox1 in RFP indicates fast lipid oxidization and decreased fat accumulation in RFP and thus may correlate with the flat phenotype, as observed in other teleosts^77,78^. This hypothesis was further supported by the lower whole body and muscular fat content in RFP compared with other nonflatfish teleosts, as observed in our analysis and in multiple other studies^79–81^, since a significantly lower fat content (*P* < 0.001) was observed in RFP than in nonflatfish outgroups in both whole body (6.22-fold low) and muscular (5.76-fold low) tissues (Fig. 3e; see Supplementary Note 24). Similar to what was observed in RFP, we also found the DGC component gene (*sntb1*)^82^ and lipogenesis-related genes (*bbox1*, *mex3c*, *faf2*, *acad11*, *elovl6* and *tysnd1*)^83–86^ to be rapidly evolving in FLP, particularly in *bbox1* (REGs, *P* = 8.75 × 10^−6^) and *mex3c* (REGs, *P* = 1.20 × 10^−5^) (Supplementary Table 91 and Supplementary Note 20). These observations suggest that a similar mechanism might have been involved in the evolution of flat body plan in both RFP and FLP. Taken together, our analyses provide evidence that marked changes in musculature development and lipid accumulation genes have occurred in flatfishes, and thus may correlate with the evolutionary origin of their body flatness (Fig. 3f).

### Genetic changes correlate with asymmetric body plan in flatfishes

**Fig. 4:**
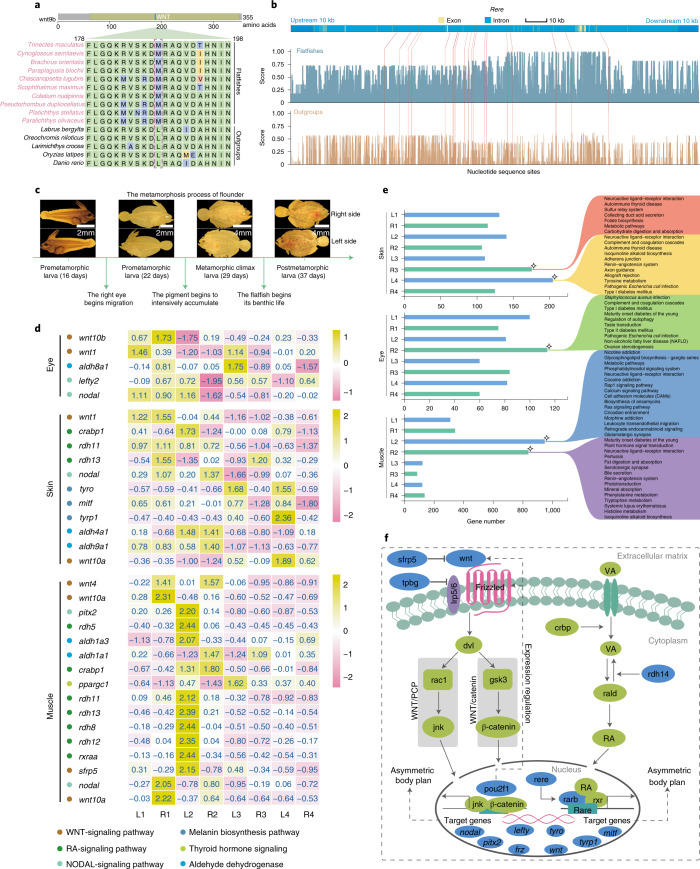
Genetic changes associated with asymmetric body plan in flatfishes. **a**, Mutations of *wnt9b* in RFP compared with outgroups. The diagram of protein structure is shown on the top of the graph, and the site that showed variation is marked with a dashed box. **b**, SCNEs nearby the *rere* gene. The *x* axis represents the nucleotide sequence sites across *rere*; the *y* axis represents sequence similarity scores. The blue and brown columns represent the average sequence similarity score within RFP and outgroups for each site, respectively; the red lines represent the physical locations of the SCNEs across the gene. **c**, Metamorphic process of flounder. Images of flatfish in premetamorphic stage, prometamorphic stage, metamorphic climax stage and postmetamorphic stage are shown. **d**, Left–right asymmetrical expression of genes in eye, skin and muscle tissues of metamorphic larvae of flounder. The numbers 1–4 on the *x* axis represent the four metamorphic stages of flounder. L and R on the *x* axis represent tissues on the left side and right side of the larva, respectively. **e**, Number of the specifically highly expressed genes during metamorphosis of flounder. The right diagram represents the biological processes in which these genes were involved. The rhombus symbols mark the stages during which the number of the genes began to show greatest expression changes. **f**, Hypothetical signaling pathway that may correlate to the body plan asymmetry in flatfishes. Proteins marked blue are those encoded by genes that experienced genetic alteration in RFP. Genes marked blue and italicized are regulating targets of these proteins and also exhibited abnormal expression (left–right asymmetry) during metamorphosis in RFP.

**Extended Data Fig. 5:**
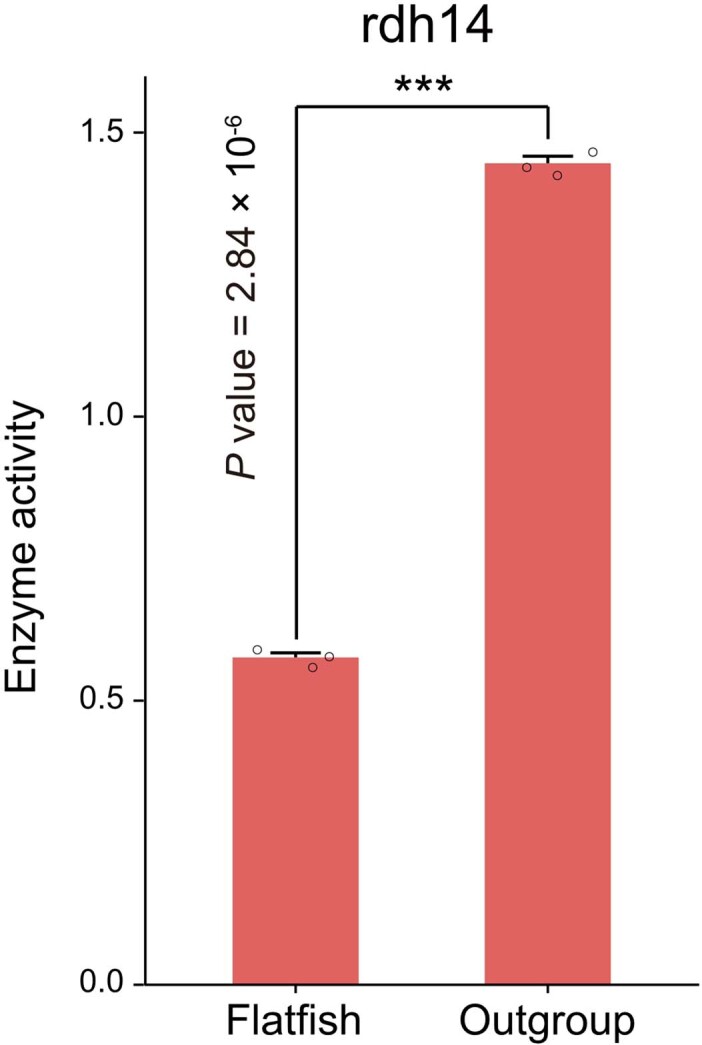
The catalyzing efficiency of enzyme rdh14 in RFP compared to that of the outgroups. The x-axis represents the RFP specific rdh14 and that of the outgroups. The y-axis represents the measured relative catalyzing efficiency of rdh14. Each experiment was performed with three reaction replicates to determine the mean values ± SD. The distinction of enzyme catalytic activity of RFP compared to non-flatfish teleosts was tested using Student’s *t*-test (two tails) with ‘***’ represents a statistical significance of *P* value < 0.001. Source data

The body asymmetry is another striking feature of flatfishes, yet its genetic basis remains largely unknown since the time of Darwin^8^. Recently, advances have been made in understanding the genetic regulation of body asymmetry in animals. Such regulation involves several gene families and signal pathways, such as RA, WNT and NODAL^19,87,88^. Both NODAL and RA signals have also been implicated in the body plan asymmetry of flatfishes^16–19^. Our comparative genomic analyses showed that multiple genes from WNT and RA signal pathways have undergone remarkable genetic alterations in RFP (see Supplementary Notes 20–22), suggesting their roles in the evolution of asymmetric body plan. These WNT-signaling genes include *wnt9b* (LSGs, L188M), *sfrp5* (LSGs, K236R), *tpbg* (PSGs, *P* = 8.02 × 10^−4^), *pou2f1* (REGs, *P* = 2.94 × 10^−3^) (Fig. 4a, Supplementary Tables 90 and 96 and Supplementary Notes 20 and 21), which encode either ligands (for example, wnt9b) or direct modulators (for example, pou2f1 (ref. ^89^), tpbg (ref. ^90^) and sfrp5 (ref. ^91^)) of the WNT-signaling pathway. Defects or expression disruptions of *wnt9b*, *tpbg*, *sfrp5* and *pou2f1* genes lead to deficiency in the WNT signal pathway, and bilateral craniofacial asymmetry and skull malformation in vertebrates^92,93^, including zebrafish^94^. Our analyses also revealed substantial changes in physicochemical properties and three-dimensional structure of these WNT components in RFP (for example, T212P and P428S cause polarity changes of amino acids in pou2f1; T169K caused charge changes in tpbg; K236R caused protein structure changes in sfrp5) (Supplementary Figs. 19–22). The alterations of so many axial-patterning WNT-signaling pathway genes may indicate their role in the body plan asymmetry of RFP. Similarly, three RA-signaling pathway genes have also undergone significant alteration in RFP, that is, *rdh14* (REGs, *P* = 3.69 × 10^−3^), *rere* (SCNEs) and *rarb* (SCNEs) (Fig. 4b, Supplementary Tables 90, 98 and 99 and Supplementary Notes 20 and 22). These genes encode core components in the RA signal pathway^95–97^ and defects in *rdh*, *rere* or *rar* genes were observed to cause RA-signaling alteration, which results in multiple congenital abnormalities, including bilateral asymmetry of eyes, craniums or somites in vertebrates^97,98^. Our enzyme catalytic activity assay further lends support for such functional alterations in these RA-signaling genes. Compared with the outgroups, RFP-specific rdh14 has much lower (2.51-fold low; *P* = 2.84 × 10^−6^) activity catalyzing retinaldehyde into retinol (Extended Data Fig. 5 and Supplementary Note 25), implying more retinaldehyde (substrate for RA synthesis) accumulation and thus RA signal alterations in RFP. These RFP-specific mutations in the RA-signaling genes might have played roles in the asymmetric body plan of RFP, although their actual role still awaits further verification. Interestingly, we noted only two WNT signal pathway genes, *wnt4a* (REGs, *P* = 0.00) and *tpbg* (REGs, *P* = 4.68 × 10^−2^), undergoing rapid evolution in FLP (Supplementary Table 91 and Supplementary Note 20). It remains to be elucidated whether such a distinction is related to the less extensive cranial asymmetry usually observed in FLP compared to typical RFP^14^. However, such a distinction between RFP and FLP provides further evidence for the polyphyletic origin of flatfishes.

**Extended Data Fig. 6:**
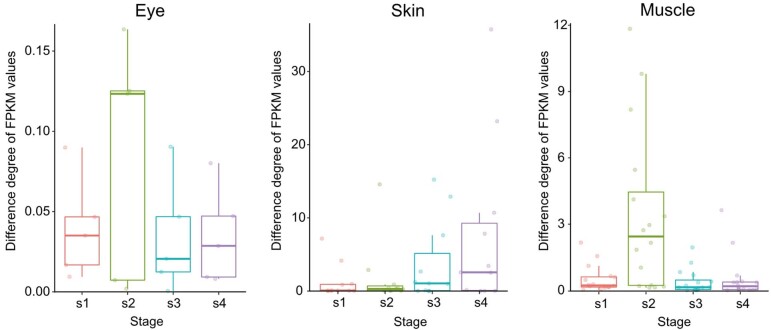
Asymmetric gene expression in metamorphic flounders. The symbol s1-s4 in the x-axis represents the four developmental stages of flounder, including the pre-metamorphic stage, the pro-metamorphic stage, the metamorphic climax stage, and the post-metamorphic stage, respectively. The y-axis represents the gene expression difference across the left-right axis of flounder, as indicated by FPKM values. The minima, maxima, centre, and the upper and lower bounds of box represent the maximum, minimum, median value, upper and lower quartile, respectively. Each colored dot represents one gene in WNT, RA, or NODAL signal pathway, or gene that associated with pigmentation that are equivalent to those indicated in Fig. 4d in the main text.

**Extended Data Fig. 7:**
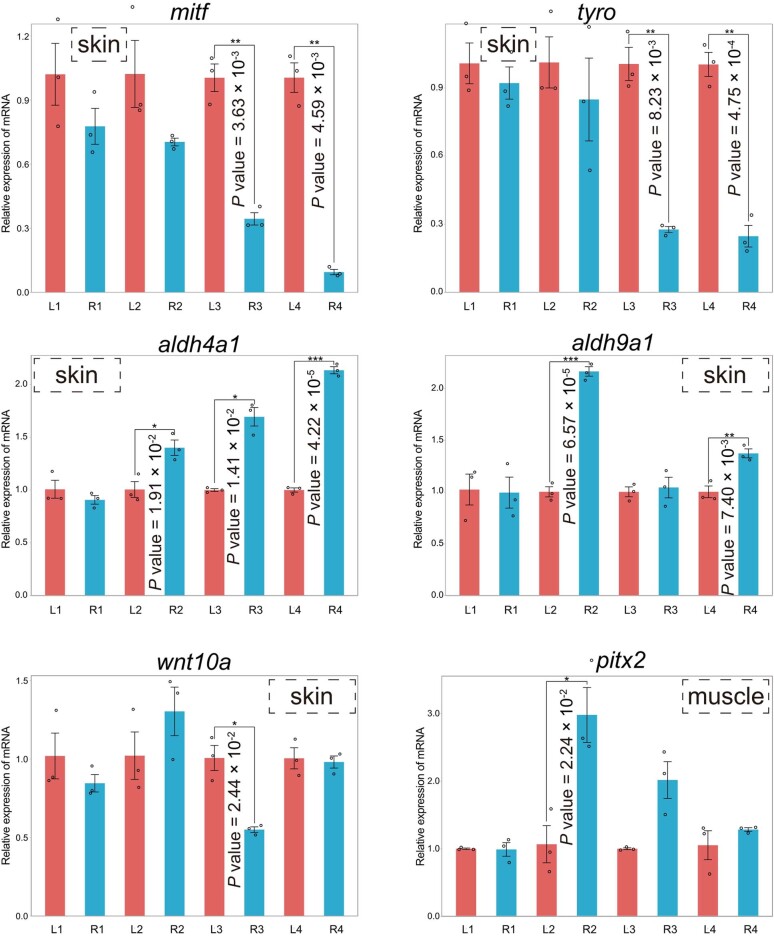
Asymmetric gene expression confirmed by Real-time quantitative PCR. For each metamorphic time window, three biological replicates were sampled, with each replicate containing tissues from at least 30 individuals, and were used for the RNAs extraction and q-PCR analysis. The numbers 1-4 in the x-axis represent the four developmental stages of flounder, including the pre-metamorphic stage, the pro-metamorphic stage, the metamorphic climax stage, and the post-metamorphic stage. L and R in the x-axis represent tissues on left-side and right-side of the larva, respectively. The y-axis represents the relative expression level of genes compared with internal control of beta-actin and the data are presented as mean values ± SD. The left-right distinction of gene expression profiles in each metamorphic time window was tested using Student’s *t*-test (two tails), and ‘*’, ‘**’, and ‘***’ represents a statistical significance of *P* value < 0.05, 0.01, and 0.001, respectively. Source data

Our transcriptomic analyses lend further support to the involvement of WNT and RA signaling in the body plan asymmetry of flatfishes. Using *Paralichthys olivaceus* as a representative example, we show that multiple genes in both RA- (*aldh1*, *aldh8*, *rdh5*, *rdh7*, *rdh8*, *rdh11*, *rdh12*, *rdh13*) and WNT- (*wnt1*, *wnt4*, *wnt10*) signaling pathways exhibited obvious transient expression fluctuations in all three examined flounder tissues (eye, muscle, skin) during metamorphosis, with marked left–right asymmetrical expression (both in gene expression level and in specific highly expressed gene number) initiating from the premetamorphic stage, climbing to an asymmetrical climax during the prometamorphic and metamorphic climax stage and then recovering to symmetry in the postmetamorphic stage (Fig. 4c–e, Extended Data Fig. 6, Supplementary Figs. 25 and 26, Supplementary Tables 100–124 and Supplementary Notes 26 and 27). Such gene expression asymmetry and fluctuations during metamorphosis were further confirmed by our real-time quantitative PCR analysis (Extended Data Fig. 7 and Supplementary Note 28). The conspicuous asymmetrical expression of these genes observed during metamorphosis (Fig. 4d,e, Extended Data Figs. 6 and 7 and Supplementary Notes 27 and 28) indicating gradients of WNT and RA signals across the left–right axis, may be related to eye migration, cranium deformation and lopsided pigmentation during metamorphosis. This is again supported by the evidence that the left deviation of expression of pigmentation genes, such as *tyro*^99^, *mitf*^99^ and *tyrp1* (ref. ^99^), usually occurs after the asymmetrical expression of RA and WNT signals in the skin of metamorphosing flounder larvae (Fig. 4c,d, Extended Data Fig. 7 and Supplementary Notes 27 and 28). Left–right asymmetric expression of NODAL-signaling genes (including *nodal*, *lefty* and *pitx2*) was also observed in the tissues of metamorphic flounder larvae (for example, muscles and eyes) (Fig. 4d). Such obvious reactivation of NODAL signaling in metamorphosis, which is not usually observed in teleosts with a regular body plan^18^, is believed to have initiated the left–right asymmetry of flatfishes^16–18^. Yet it remains an open question as to whether such reactivation of NODAL signals can also be attributed to the asymmetrical RA and WNT signals, although cross-talk between them has long been documented in diverse taxa^100,101^. Taken together, our analyses provide gene evolution and expression evidence for the possible involvement of WNT combined with RA-signaling pathways in shaping the asymmetric body plan in flatfishes (Fig. 4f), although the exact role of these RA and WNT genes in the body plan asymmetry still awaits further investigation.

### Genetic changes associated with the modified fins and over-substrate maneuvering of flatfishes

**Extended Data Fig. 8:**
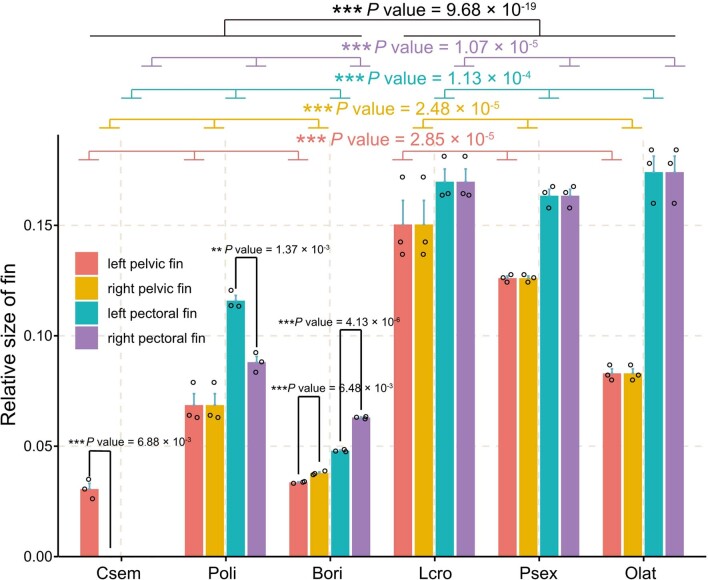
Relative size of pelvic and pectoral fins in flatfishes compared to non-flatfish species. The x-axis in the panel shows the species with pectoral and pelvic fins measured, and the y-axis shows the relative sizes of fins represented by the ratios of length of pectoral and pelvic fins to the total length of the fish. All the parameters were measured in three individuals for each species and the data are presented as mean values ± SD. The distinction of fin morphology of flatfishes compared to non-flatfish teleosts was tested using Student’s *t*-test (two tails) with ‘**’ and ‘***’ representing a statistical significance of *P* value < 0.01 and *P* value < 0.001 respectively. Csem (*Cynoglossus semilaevis*); Poli (*Paralichthys olivaceus*); Bori (*Brachirus orientalis*); Lcro (*Larimichthys crocea*); Psex (*Polydactylus sextarius*); Olat (*Oryzias latipes*). Source data

**Extended Data Fig. 9:**
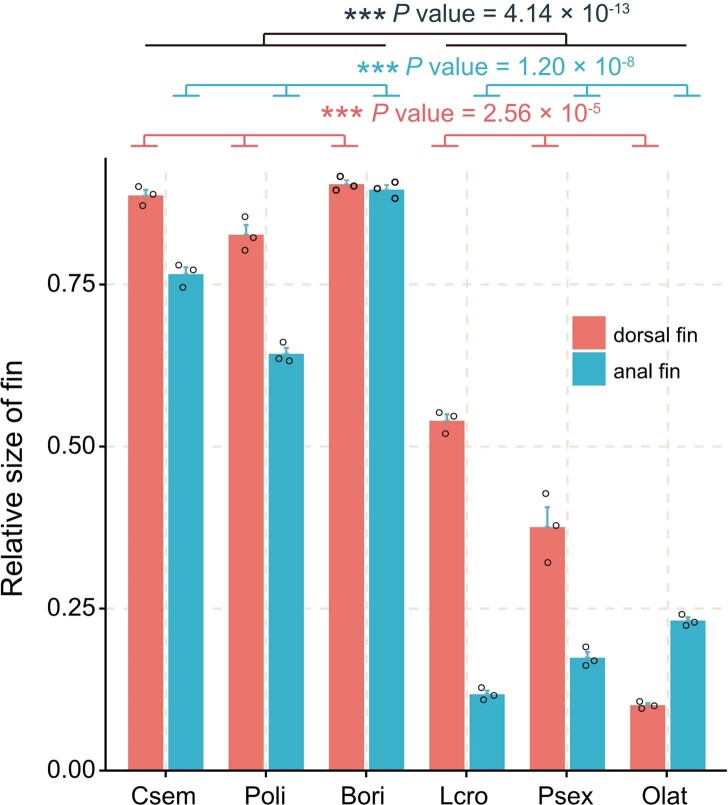
Relative sizes of dorsal and anal fins in flatfishes compared to non-flatfish species. The x-axis in the diagram shows the species with dorsal and anal fins measured, and the y- axis in the diagram shows the relative sizes of fins represented by the ratios of length of dorsal and anal fins to the total length of the fish. All the parameters were measured in three individuals for each species and the data are presented as mean values ± SD. The distinction of fin morphology of flatfishes compared to non-flatfish teleosts was tested using Student’s *t*-test (two tails) with ‘***’ representing a statistical significance of *P* value < 0.001. Csem (*Cynoglossus semilaevis*); Poli (*Paralichthys olivaceus*); Bori (*Brachirus orientalis*); Lcro (*Larimichthys crocea*); Psex (*Polydactylus sextarius*); Olat (*Oryzias latipes*). Source data

**Extended Data Fig. 10:**
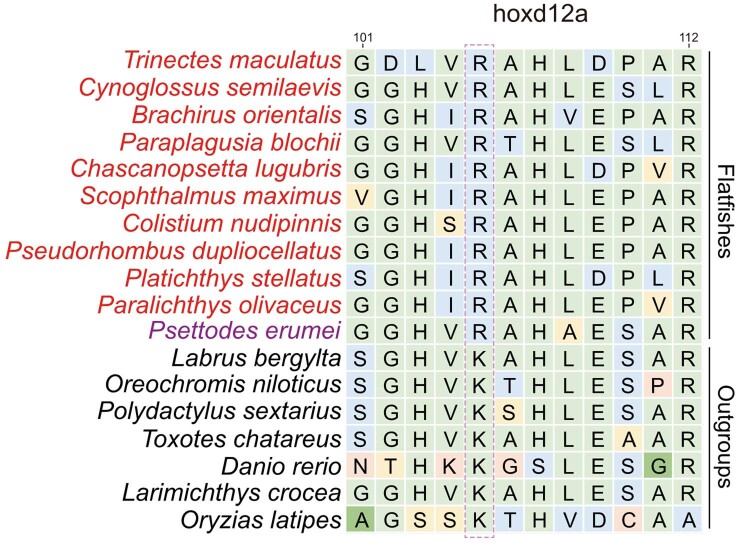
The lineage specific mutated gene of *hoxd12a* in real flatfish species. The sites that showed variation between species are marked in different colors. The fixed variation site between real flatfish Pleuronectoidei species and outgroups are marked with a dashed box.

To fit their specialized body plan, flatfishes have also evolved a new vertebrate gait of ‘fin-feet’ walking, which enables their flexible horizontal maneuvering over substrates^5,6^ while keeping both of their eyes alert from above^60^. Such fin-feet walking was attributed to their largely elongated median fins^11^ and their largely reduced paired fins (for example, pectoral fins^11^) (Extended Data Figs. 8 and 9, Supplementary Fig. 27 and Supplementary Note 29), since these specialized fins enable a repeated generation of the ‘fin-feet’ (mainly by dorsal and anal fins) pushing down against the substrate to produce constant forward movement while keeping an accurate maneuvering orientation (mainly by pectoral fins)^5,6^. However, the genetic basis of such fin phenotypes in flatfishes is unknown. Our comparative genomic analyses revealed two genes, including *hoxd12a* (K105R) and *bhlha9* (PSGs, *P* = 9.38 × 10^−3^), underwent considerable changes in RFP (Extended Data Fig. 10, Supplementary Tables 90 and 97 and Supplementary Notes 20 and 21). Among them, *hoxd12a* is closely associated with fin patterning and morphogenesis in teleosts^102^, since it encodes a DNA-binding transcription factor essential for regulation of the anterior–posterior pattern of fins^102^. The alterations in *hoxd12a* were believed to account for the forelimb (homologs of teleost fins) reorganization in cetaceans^103^ and paired fin degeneration in lungfishes^104^. Furthermore, *hoxd12a* has also been implicated in dorsal fin development in flounders^105^. The observed mutations in *hoxd12a* may have implications for the morphological changes of median and paired fins in RFP (Supplementary Table 97 and Supplementary Note 21), although the causative effect of these mutations still awaits further verification. Similarly, *bhlha9* encodes a transcription factor closely related to fin morphogenesis. Knockdown of *bhlha9* in zebrafish usually results in a size reduction of pectoral fins^106^. The observed positive selection in *bhlha9* gene of RFP may also indicate its possible role in fin modification (Supplementary Table 90 and Supplementary Note 20). The *bhlha9* gene is also rapidly evolving (*P* = 2.03 × 10^−4^) in FLP (Supplementary Table 91 and Supplementary Note 20), and *hoxd12a* experienced convergent variation between FLP and RFP (Supplementary Table 97 and Supplementary Note 21), indicating a possible role of these two genes in shaping the specialized fin morphologies of RFP and FLP.

## Discussion

Our study demonstrates the strengths of combining phylogenomics and comparative genomics to shed light on the evolutionary history and mechanisms of a nonmodel taxon with complex adaptive traits, such as flatfishes. Using large-scale genomic data, we revealed a polyphyletic origin of flatfishes, with real flatfish Pleuronectoidei and flatfish-like Psettodoidei independently evolving from their different percoid ancestors. However, Pleuronectoidei and Psettodoidei also share convergent alterations in genes related to muscular development, lipid accumulation, body axis determination and fin pattern regulation. Meanwhile, Psettodoidei also exhibited unique mutations that may contribute to their less asymmetric body plan compared to Pleuronectoidei. The results obtained in this study have substantially clarified the long-standing controversies over the phylogeny of flatfishes, while the genes highlighted in this study lay a blueprint for future functional characterization of the molecular mechanisms underlying the unusual body plan of flatfishes. The genetic basis of such complex traits in flatfishes will not only enrich our knowledge on how the symmetric body plan that dominates the animal kingdom has evolved, been retained and modified, but also potentially help to unveil congenital causes of similar human pathological disorders, such as muscular atrophy and craniofacial malformations.

## Methods

### DNA and RNA extraction

Genomic DNA was isolated from muscle tissues using the classic phenol–chloroform method. Total RNA was extracted using a Trizol kit (Life Technologies). The quality and quantity of extracted DNA/RNA were assessed using an Agilent 2100 bioanalyzer (Agilent Technologies), and their integrity was further evaluated on agarose gel stained with ethidium bromide. The extracted DNA/RNA samples were stored at −80 °C until subsequent library construction and genome/transcriptome sequencing. All tissue sampling and DNA and RNA extraction processes complied with all relevant ethical regulations provided by the Institutional Animals Care and Use Committee of Zhejiang Ocean University and by the Experimental Animal Management and Ethics Committee of South China Sea Institute of Oceanography, Chinese Academy of Sciences.

### Library construction and sequencing

For genome sequencing of the seven species of *Trinectes maculatus*, *Chascanopsetta lugubris*, *Brachirus orientalis*, *Paraplagusia blochii*, *Colistium nudipinnis*, *Pseudorhombus dupliocellatus* and *Platichthys stellatus*, both the short-insert (350–700 bp) and long-insert (>1 kb) paired-end libraries of each species were constructed from the extracted genomic DNA of each species using the Illumina library construction kit (NEBNext Ultra DNA Library Prep Kit from Illumina, catalog no. E7370S) and sequenced on the Illumina HiSeq 4000 platform. For genome sequencing of the three species of *Psettodes erumei*, *Toxotes chatareus* and *Polydactylus sextarius*, the extracted genomic DNA was size-selected using PippinHT (Sage Science). Then, the Nanopore libraries were constructed and sequenced on PromethION DNA sequencer (Oxford Nanopore Technologies). Genomes of the three species of *Platichthys stellatus*, *Toxotes chatareus* and *Polydactylus sextarius* were further sequenced on the Hi-C platform to obtain chromosome-level genome assemblies. For Hi-C library construction, DNA extracted from each species was fragmented and purified using magnetic beads. Hi-C libraries were sequenced on the Illumina HiSeq 4000 platform with 150-bp paired-end reads. For RNA sequencing of the four species *Platichthys stellatus*, *Toxotes chatareus*, *Polydactylus sextarius* and *Paralichthys olivaceus*, the complementary DNA libraries were constructed from RNA extracted from various tissues, such as eye, liver, muscle and skin, as indicated in Supplementary Table 2 for different analysis purposes according to the manufacturer’s instructions (NEBNext Ultra RNA Library Prep Kit from Illumina, catalog no. E7530S) and sequenced on the Illumina HiSeq 4000 platform.

### Quality control of sequencing data

For Illumina sequencing reads, all low-quality reads, duplicated reads and adapter sequences were removed using Perl scripts. For Nanopore long reads, mean quality for each read was calculated and only reads longer than 1 kb with mean quality ≥7 were retained. For Hi-C sequencing data, the low-quality reads were further filtered using Hi-C-Pro software (v.3.2)^107^ after prefiltering with Perl scripts.

### Genome size estimation

Genome size of each species was estimated using the short-insert library reads by the *k*-mer method. The 17-mer was chosen for *k*-mer analysis in this study, and the genome size (*G*) was estimated with the following formula: *G* = *K*_num_/*K*_depth_, where *K*_num_ and *K*_depth_ represent the total number of 17-mers and the peak of depth of the 17-mer, respectively.

### Genome assembly and chromosome construction

For the genome assembly, seven species (*Trinectes maculatus*, *Chascanopsetta lugubris*, *Brachirus orientalis*, *Paraplagusia blochii*, *Colistium nudipinnis*, *Pseudorhombus dupliocellatus* and *Platichthys stellatus*) were assembled with Illumina short reads using the Platanus software (v.1.2.4)^108^, and all the cleaned short reads were used to fill the gaps of the genome using Gapcloser (v.1.10). Three species (*Psettodes erumei*, *Toxotes chatareus* and *Polydactylus sextarius*) were assembled with Nanopore long reads using WTDBG software (v.1.2.8)^109^, and all the cleaned Illumina short-insert reads were aligned to the assembled contigs to conduct error correction. For chromosome construction of three species (*Platichthys stellatus*, *Toxotes chatareus* and *Polydactylus sextarius*), the filtered Hi-C reads were aligned to the assembled genome and then anchored to chromosomes using three-dimensional de novo assembly software (v.170123)^110^.

### Ancestral chromosome reconstruction

First, the chromosome-level genomic data of *Platichthys stellatus* and *Cynoglossus semilaevis*, in the real flatfish Pleuronectoidei lineage, and *Toxotes chatareus* and *Polydactylus sextarius* (sequenced in this study), leading to the flatfish-like Psettodoidei lineage, were aligned and the genome synteny was analyzed using LAST^111^ with the parameters of --k 1 -m 10 --E 0.05. Then, the chromosome variation events within and between lineages were compared using ANGES (v.1.01)^112^ to detect the lineage-specific chromosome variation. Finally, contig sequences obtained from Nanopore reads of *Psettodes erumei* were used to check for these lineage-specific chromosome fusion and fission events to further test if flatfish-like Psettodoidei lineage (including *Psettodes erumei*) has different ancestral chromosomes from that of real flatfish Pleuronectoidei.

### Genome annotation

Repetitive sequences were identified using different software programs. Transposable elements (TEs) were annotated on both protein and DNA levels. On the protein level, the RepeatProteinMask (RM-BLASTX) was used to search TEs in its protein database. On the DNA level, RepeatModeler software (v.1.0.8) was used to build de novo repeat library and RepeatMasker (v.4.0.6)^113^ was then run against the de novo library and repbase (RepBase v.16.02) separately to identify homologous repeats. Protein-coding genes were annotated using three combined approaches, including de novo prediction, homology-based annotation and/or transcripts-based annotation from the repeats-masked genome. For de novo prediction, Augustus (v.3.2.1)^114^ and GENSCAN (v.1.0)^115^ were used. For homology-based annotation, protein sequences of seven species (*Mus musculus*, *Gallus gallus*, *Callorhinchus milii*, *Takifugu rubripes*, *Lepisosteus oculatus*, *Cynoglossus semilaevis* and *Paralichthys olivaceus*) were downloaded from NCBI and protein sequences of one species (*Danio rerio*) were downloaded from Ensembl. The longest transcript of each gene was selected and any genes with early termination sites were removed. All remaining genes were aligned to the repeat-masked genome for homology-based annotation using tblastn with *e*-value less than 1 × 10^−5^. Genewise software (v.2.2.0)^116^ was used to identify the longest coding regions and/or highest score in each gene locus to support the presence of a homologous gene. For transcript-based annotation, cleaned RNA-seq reads were assembled into transcripts, and then were aligned against the assembled genome to link spliced alignments. EvidenceModeler (v.1.1.1)^117^ was used to integrate the results derived from these methods into the final gene set. Functions of these predicted genes were analyzed using the public protein databases. InterProScan (v.4.8) was used to screen proteins against databases (Pfam, v.27.0; prints, v.42.0; prosite, v.20.97; ProDom, v.2006.1; smart, v.6.2). In addition, the Kyoto Encyclopedia of Genes and Genomes (KEGG), NR, SwissProt (v.2011.6) and TrEMBL (v.2011.6) databases were also searched for homology-based function assignments using BLAST software (v.2.6.0) with *e*-value of 1 × 10^−5^.

### Identification of orthologous genes

Orthologs were identified in the assembled genomes of ten sequenced species, along with the species with published genome sequences (*Cynoglossus semilaevis*, *Paralichthys olivaceus*, *Scophthatmus maximus*, *Danio rerio*, *Larimichthys crocea*, *Labrus bergylta*, *Oreochromis niloticus* and *Oryzias latipes*) using the OrthoMCL pipeline (v.2.0.9)^118^. Briefly, all the protein-coding genes of the published species were downloaded from the NCBI database, except for *Scophthatmus maximus*, which was downloaded from its own website (http://denovo.cnag.cat/genomes/turbot). To improve the accuracy of the analysis, genes that encode shorter than 30 amino acids or have early stop codons in the coding regions were removed. All the remaining genes were aligned and reciprocally compared, and the reciprocal best similarity pairs among species were considered as putative orthologs after further evaluation using MCscan software (v.0.9.13)^119^.

### Phylogenetic tree construction and divergence time evaluation

All the 1,693 single-copy homologous genes identified among species (*Trinectes maculatus*, *Chascanopsetta lugubris*, *Brachirus orientalis*, *Paraplagusia blochii*, *Colistium nudipinnis*, *Pseudorhombus dupliocellatus*, *Platichthys stellatus*, *Psettodes erumei*, *Polydactylus sextarius*, *Toxotes chatareus*, *Cynoglossus semilaevis*, *Paralichthys olivaceus*, *Scophthatmus maximus*, *Danio rerio*, *Larimichthys crocea*, *Labrus bergylta*, *Oreochromis niloticus* and *Oryzias latipes*) were aligned and concatenated into supergenes for phylogenetic relationship analyses. Maximum likelihood-based phylogenetic analysis was conducted using RAxML (v.8.2.9)^120^. Meanwhile, species trees were also constructed using MPEST (v.2.0)^20^ and OrthoFinder (v.2.3.5)^21^. Divergence times of these species were then estimated on the basis of the 4dTV sequences via Bayesian relaxed molecular clock approach using MCMCtree program in the PAML package (v.4.8)^121^. Fossil records downloaded from the TIMETREE website (http://www.timetree.org) were used for calibrating our calculated divergence time.

### Estimation of relative evolutionary rates

The relative evolutionary rates of species were calculated using two-cluster analysis and Tajima’s relative rate test. Two-cluster analysis was performed to test molecular evolution of multiple sequences in the phylogenetic context. A faster or slower evolutionary rate in a particular taxon was analyzed using *Z*-statistics and tpcv module in the LINTRE program. For Tajima’s relative rate test, a higher number of lineage-specific substitutions indicates a much faster evolutionary rate using the chi-squared test. All the single-copy genes were used in these two analyses with zebrafish as the outgroup species.

### Estimation of gene family expansion and contraction

Expansion and contraction of gene clusters was determined using the CAFE software (v.3.1)^122^. The phylogenetic tree and divergence time analyzed in the previous steps were used in CAFE to infer changes in gene family sizes using a probabilistic model.

### Detection of positive selection

All one-to-one orthologous genes extracted from flatfish species and outgroup species (*Larimichthys crocea*, *Labrus bergylta*, *Oreochromis niloticus*, *Oryzias latipes* and *Danio rerio*) were used to identify positively selected or rapidly evolving genes. The multiple sequence alignments were generated and used to estimate three types of *ω* (the ratio of the rate of nonsynonymous substitutions to the rate of synonymous substitutions) using branch model in the codeml program of the PAML package (v.4.8)^121^. Branch model (model = 2, NSsites = 0) was used to detect *ω* of appointed branch to test (*ω*0) and average *ω* of all the other branches (*ω*1) and the mean of whole branches (*ω*2). Then *χ*^2^ test was used to check whether *ω*0 was significantly higher than *ω*1 and *ω*2 under the threshold *P* value <0.05, which hinted that these genes would be under positive selection or fast evolution.

### Identification of genes with lineage-specific mutation

The high-quality alignments were also used to identify the lineage-specific mutated genes. In this analysis, all single-copy genes among species were checked and any genes with the same variation across all particular taxa, compared with outgroup species, were identified as LSGs. Candidate LSGs were further double-checked using original Illumina reads to avoid assembly and sequencing errors. In addition, Bayesian ancestral state inference conducted using the codeml program in PAML software (v.4.8)^121^ was further used to validate the candidate LSGs. In the Bayesian framework, the ancestral state was inferred by the state with the highest posterior probability. In our case, only the ancestral state of Pleuronectoidei was different from the ancestor of all the Pleuronectiformes species, *Toxotes chatareus* and *Polydactylus sextarius*; the potential LSGs were therefore recognized as the true Pleuronectoidei LSGs.

### Identification of conserved noncoding elements

Using the *Platichthys stellatus* genome as the reference, the genomes of flatfish and outgroup species were aligned to the reference genome using LAST software (v.802)^111^ with the following parameters: -P 5 -m 100 -E 0.05.

The generated alignments were checked locus by locus, and the loci that were present in more than eight Pleuronectoidei species, but absent in any nonflatfish species, were recognized as the potential Pleuronectoidei-specific conserved noncoding elements. Any SCNE sequences less than 20 bp were removed to ensure the accuracy of identification.

### Gene expression profile analysis

RNA extracted from eye, skin and muscle tissues across the left–right axis in different metamorphic time windows (premetamorphic larva, prometamorphic larva, metamorphic climax larva and postmetamorphic larva) of *Paralichthys olivaceus* was sequenced on the Illumina sequencing platform. For each metamorphic time window, three biological replicates were sampled, with each replicate containing tissues from at least 30 individuals because of the small size of the larvae, and was used for the RNA extraction and sequencing. Raw reads were filtered and remaining high-quality reads were aligned to the assembled genome using Tophat2 (v.2.1.1)^123^. The transcripts were assembled and gene expression values were analyzed using the cufflinks software (v.2.2.1)^124^.

### Real-time quantitative PCR assay

Real-time quantitative PCR (qPCR) was used to verify the differentially expressed genes across the left–right body axis of *Paralichthys olivaceus*. Samples were collected as indicated above and extracted RNA was used for obtaining cDNA using the PrimeScript RT reagent kit with gDNA Eraser (Perfect Real Time) (TaKaRa, catalog no. RR047A). The qPCR analysis was performed using the TaKaRa TB Green Premix Ex TaqII (Tli RNaseH Plus) reagents (TaKaRa, catalog no. RR820A). The β-actin gene was used as the internal control of the qPCR experiment. Each experiment was performed with three reaction replicates and calculated relative expression value of genes using the detected threshold cycle (Ct) value.

### Catalytic activity assay of enzymes

In vitro enzymatic activity assay was used to test the functional consequence of RFP-specific mutation in bbox1 and rdh14 proteins. RFP-specific genes and those of the outgroups were codon-optimized according to the *Escherichia coli* preference and then synthesized and cloned into the vector pET-28a by Wuhan Gene Create Biological Engineering. The plasmid was transformed into DH5α competent cells for amplification, and then the plasmids were extracted for verification. Finally, the correct plasmids were transformed into BL21 (DE3) to be expressed. The expressed proteins were further extracted, purified and the enzyme activity was measured according to Rattner et al.^125^ and Cao et al.^126^, respectively. Each experiment was performed with three reaction replicates to determine the mean ± s.d. of the catalytic activity value of the enzymes.

### Statistical analysis

Significant differences between the groups were assessed with Student’s *t*-test (two tails). The chi-squared test or Fisher’s exact test were used in the significant analysis of gene ontology enrichment according to the data feature, and the hypergeometric test was used in KEGG. Multiple comparisons were corrected for false discovery rate. The symbols *, ** and *** represent a statistical significance of *P* values <0.05, 0.01 and 0.001, respectively.

### Reporting Summary

Further information on research design is available in the Nature Research Reporting Summary linked to this article.

## Online content

Any methods, additional references, Nature Research reporting summaries, source data, extended data, supplementary information, acknowledgements, peer review information; details of author contributions and competing interests; and statements of data and code availability are available at 10.1038/s41588-021-00836-9.

### Supplementary information

Supplementary Information Supplementary Notes 1–29 and Figs. 1–27

Reporting Summary

Peer Review Information

Supplementary Tables Supplementary Tables 1–124

### Source data

Source Data Fig. 3a Body flatness of flatfishes relative to nonflatfish species.

Source Data Fig. 3d The catalyzing efficiency of RFP-specific bbox1 and that of the outgroups revealed by in vitro enzyme activity assay.

Source Data Fig. 3e The crude fat content in flatfishes compared with nonflatfish species.

Source Data Extended Data Fig. 4 Body flatness of flatfishes relative to nonflatfish species.

Source Data Extended Data Fig. 5 The catalyzing efficiency of RFP-specific rdh14 and that of the outgroups revealed by in vitro enzyme activity assay.

Source Data Extended Data Fig. 7 Asymmetrical expression of genes confirmed by RT–PCR assay in flounder.

Source Data Extended Data Fig. 8 Relative size of pelvic and pectoral fins in flatfishes compared with nonflatfish species.

Source Data Extended Data Fig. 9 Relative size of dorsal and anal fins in flatfishes compared with nonflatfish species.

## Data Availability

All the sequencing data were deposited at the NCBI (*Platichthys stellatus*: PRJNA592732; *Trinectes maculatus*: PRJNA592733; *Brachirus orientalis*: PRJNA592734; *Paraplagusia blochii*: PRJNA592738; *Chascanopsetta lugubris*: PRJNA592739; *Colistium nudipinnis*: PRJNA592742; *Pseudorhombus dupliocellatus*: PRJNA592743; *Polydactylus sextarius*: PRJNA592744; *Toxotes chatareus*: PRJNA592745; *Psettodes erumei*: PRJNA592748; *Paralichthys olivaceus*: PRJNA632737). Besides, the source data of the fish photos were deposited at the Figshare database (10.6084/m9.figshare.13664201.v1). Source data are provided with this paper.

## Code availability

All public software used in this study is provided in the accompanying Nature Research Reporting Summary.
