# Supplementary material for: Large-scale sequencing of flatfish genomes provides insights into the polyphyletic origin of their specialized body plan
Source: Nat Genet. 2021 Apr 19;53(5):742–51. doi: 10.1038/s41588-021-00836-9 (PMC8110480; doi:10.1038/s41588-021-00836-9)
Supplement: Supplementary file 1 — Supplementary Notes 1–29 and Figs. 1–27 [file 41588_2021_836_MOESM1_ESM.pdf]

nature portfolio

https://doi.org/10.1038/s41588-021-00836-9

Supplementary information

Large-scale sequencing of flatfish genomes provides insights into the polyphyletic origin of their specialized body plan

In the format provided by the authors and unedited

Supplementary Materials

Contents

Supplementary Note 1. Sample collection and preparation

Supplementary Note 2. DNA/RNA extraction

Supplementary Note 3. Library construction and sequencing

Supplementary Note 4. Quality control of raw sequencing reads

Supplementary Note 5. Error correction for short-insert library sequencing reads

Supplementary Note 6. Draft genome assembly for Illumina sequenced species

Supplementary Note 7. Draft genome assembly for Nanopore sequenced species

Supplementary Note 8. Chromosome-level assemblies

Supplementary Note 9. Genome size estimation

Supplementary Note 10. Evaluation of assembly quality

Supplementary Note 11. Genome synteny analysis

Supplementary Note 12. Repetitive sequence annotation

Supplementary Note 13. Evolution of genome size

Supplementary Note 14. Protein-coding gene annotation

Supplementary Note 15. Functional annotation of protein-coding genes

Supplementary Note 16. Gene family analysis

Supplementary Note 17. Phylogenetic tree and ancestral chromosome construction

Supplementary Note 18. Relative evolutionary rate analysis

Supplementary Note 19. Expansion and contraction of gene families

Supplementary Note 20. Identification of positively selected and rapidly evolving genes

Supplementary Note 21. Identification of lineage-specific mutation

Supplementary Note 22. Identification of conserved non-coding elements

Supplementary Note 23. Genes associated with benthic adaptation and body plan creation

Supplementary Note 24. Measurement of body morphology and fat content

Supplementary Note 25. Catalytic activity assay of enzymes

Supplementary Note 26. Sample collection in metamorphic flounder

Supplementary Note 27. Gene expression profiles in metamorphic flounder

Supplementary Note 28. Real-time quantitative PCR

Supplementary Note 29. Measurement of the fin length

References

Supplementary Note 1. Sample collection and preparation

Tissue samples of species used in this study were collected from various sea areas. *Trinectes maculatus*, *Toxotes chatareus*, and *Colistium nudipinnis* were collected from America, Thailand, and Australia, respectively, through laboratory to laboratory sample exchange programme, and all the other samples were collected from China. All the tissue samples were transported at low temperature from sampling sites to the laboratory and stored in -80℃. Detailed information on all the samples used in genome sequencing analysis in this study is summarized in **Supplementary Table 1**. Detailed information on all the samples used in transcriptome analyses for *Platichthys stellatus*, *Toxotes chatareus*, *Polydactylus sextarius*, and *Paralichthys olivaceus* in this study is shown in **Supplementary Table 2**.

Supplementary Note 2. DNA/RNA extraction

DNA was extracted from muscle tissues of each species using classic phenol-chloroform protocol[1]. For RNA extractions in *Platichthys stellatus*, *Toxotes chatareus*, *Polydactylus sextarius*, and *Paralichthys olivaceus* (**Supplementary Table 2**), tissues from samples were ground into proper particles in a precooled mortar, and then lysed with 150 ul Trizol on an oscillator for 10 min. RNA was extracted using chloroform followed by isopropanol precipitation. After washed with 75% ethanol twice and evaporated dry in a ventilation, RNA precipitation was dissolved in 30 ul RNase-free double distilled water. The quality and quantity of extracted DNA/RNA were assessed using Agilent 2100 bioanalyzer (Agilent Technologies), and their integrity was further evaluated on agarose gel stained with ethidium bromide. The acquired DNA/RNA samples were stored at -80℃ and used for the subsequent library construction and genome/transcriptome sequencing.

Supplementary Note 3. Library construction and sequencing

The extracted DNA/RNA samples were used for library construction and sequencing. In this study, three types of sequencing libraries (Illumina, Nanopore, and Hi-C) were constructed and sequenced. The detailed information is described below:

1) Library construction and sequencing on the Illumina platform

For genome sequencing, four different short-insert libraries (350-700 bp) and 2-5 long-insert libraries (> 1 kb) were constructed for the seven species of *Trinectes maculatus*, *Chascanopsetta lugubris*, *Brachirus orientalis*, *Paraplagusia blochii*, *Colistium nudipinnis*, *Pseudorhombus dupliocellatus*, and *Platichthys stellatus*, and one short-insert library was constructed for the three species of *Psettodes erumei*, *Toxotes chatareus*, and *Polydactylus sextarius* (see **Supplementary Table 1**). The libraries were constructed following the protocol provided by Library Preparation Kit (NEBNext® Ultra™ DNA Library Prep Kit for Illumina®, # E7370S). These constructed libraries were sequenced on Illumina HiSeq 4000 platform. For RNA sequencing, total mRNA (*Platichthys stellatus*, *Toxotes chatareus*, *Polydactylus sextarius*, and *Paralichthys olivaceus*, see **Supplementary Table 2**) were used for library construction and subsequent sequencing. The detailed procedure for the library preparation followed the protocol provided by Library Prep Kit (NEBNext® Ultra™ RNA Library Prep Kit for Illumina®, #E7530S). The quality of constructed libraries was examined on Agilent 2100 bioanalyzer. The libraries were then sequenced on the Illumina HiSeq 4000 platform.

2) Library construction and sequencing on the Nanopore platform

Genomes of three species, including *Psettodes erumei*, *Toxotes chatareus*, and *Polydactylus sextarius*, were further sequenced on the Nanopore platform (see **Supplementary Table 1**). Briefly, the high molecular weight genomic DNA was size-selected using the PippinHT (Sage Science, Beverly, MA, USA). The resulting DNA fragments were repaired using the NEBNext FFPE Repair Mix (M6630). After further end-repaired and appended with dATP through the NEBNext Ultra II End repair/dA-tailing Module (E7546), the DNA fragments were ligated to the Oxford Nanopore sequencing adapters using the library prepared kit SQK-LSK109 (Oxford Nanopore Technologies) following the manufacture’s instruction. Finally, the prepared libraries were sequenced on the PromethION DNA sequencer (Oxford Nanopore Technologies) platform.

3) Library construction and sequencing on the Hi-C platform

Genomes of three species, including *Platichthys stellatus*, *Toxotes chatareus*, and *Polydactylus sextarius*, were further sequenced using the Hi-C (High-throughput/resolution chromosome conformation capture) platform and assembled into the chromosome level (see **Supplementary Table 1**). Briefly, the muscle tissues were ground and cross-linked using 37% formaldehyde (in a final concentration of 1%). After stopping the cross-linking reaction by glycine, the restriction enzymes MboI, MboI, and DpnII, were used for *Platichthys stellatus*, *Toxotes chatareus*, and *Polydactylus sextarius*, respectively to digest the genomic DNA. The resulting DNA was incubated with biotin-labeled dATP and ligation enzyme was used to ligate adjacent DNA fragments. Proteinase K was then used to digest the protein followed by pre-incubation at 68℃ for 45 minutes. The extracted fragmented DNA (approximately 350 bp) was purified using the magnetic beads, and then connected to sequencing adapters. The resulting Hi-C libraries were sequenced on the Illumina sequencing platform (Illumina, San Diego, CA, USA).

Supplementary Note 4. Quality control of raw sequencing reads

Sequencing errors can create difficulties for short-read assembly algorithm. To ensure the accuracy of genome assembly, low quality reads need to be removed before assembly. The sequencing data sets produced from different platforms (Illumina, Nanopore, and Hi-C) were filtered as described below.

1) Checking and filtering for Illumina sequencing data

The quality of Illumina sequencing data was checked using Perl scripts. Reads that met the following criteria were removed: 1) Read pairs containing more than 30% of low quality bases; 2) Read pairs containing more than 10 percent “N”; 3) PCR duplicates. The statistics of cleaned sequencing data are shown in **Supplementary Tables 3-11**.

2) Checking and filtering for Nanopore sequencing data

The quality of sequencing data produced from the PromethION (Oxford Nanopore Technologies) platform was directly evaluated by the mean Q-score, which displayed in the quality value file for each read (*.txt* file). Reads with the mean Q-score < 7 or length shorter than 1,000 bp were removed, and the remaining reads were used for the subsequent genome

assembly and other analyses (**Supplementary Table 12**).

3) Checking and filtering for Hi-C sequencing data

The quality of sequencing data produced from the Hi-C platform was checked and filtered by the HiC-Pro software (v3.2)[2] with default parameters, following the criteria described above for the Illumina sequencing reads. Statistics of the cleaned Hi-C sequencing data are summarized in **Supplementary Table 13**.

Supplementary Note 5. Error correction for short-insert library sequencing reads

Errors in reads produced from sequencing can be corrected based on kmer frequency information. Usually, sites with sequencing errors occur at much lower frequency than normal reads (which occur at high frequency) in the kmer distribution. We corrected sequencing errors by replacing the low-frequency reads with the high-frequency reads. For example, if there was a base error ('A') in a read in which the 17-mer frequency was ≤ 12X, the base was replaced with 'T', 'G' and 'C'. If one of the replacements resulted in a 17-mer frequency > 12X, it would be used as a replacement. If more than one alternative pattern of the replacements satisfied the condition of > 12X, we used the pattern occurring at the highest frequency. In the present study, a parameter of 17-mer was selected for sequencing error corrections using SOAPec software (v2.03), and all the Illumina short-insert reads were scanned for corrections.

Supplementary Note 6. Draft genome assembly for Illumina sequenced species

Genomes of all species sequenced on the Illumina platform, including *Trinectes maculatus*, *Chascanopsetta lugubris*, *Brachirus orientalis*, *Paraplagusia blochii*, *Colistium nudipinnis*, *Pseudorhombus dupliocellatus*, and *Platichthys stellatus* were assembled using short-insert and long-insert library sequencing reads through Platanus software (v1.2.4)[3], which was specifically developed to assemble the highly heterozygous diploid genomes using shotgun sequencing data. GapCloser software (v1.10) was used to fill the gaps between scaffolds in the assembled genome. Detailed procedures for the genome assembly are described as below:

1) Contig construction: The filtered and error-corrected sequencing reads produced from

short-insert libraries (< 1 kb) were split into kmers and used to generate the *de Bruijn* graph using the Platanus software with default settings except for the "k" and "u" parameters. Short branches and bubbles were easily recognized during graph construction. The short branches produced by errors were removed by the "tip removal", and bubbles were also removed by the Platanus software. The final subgraphs that had no junctions represent the contigs.

2) Scaffold construction: All the cleaned Illumina reads were aligned to the contig sequences. Any two contigs that could be aligned by the same one paired-end read were then connected into the same scaffold. Bubbles produced in the assembly process were removed by the "bubble removal" or "branch cut" step in the Platanus software.

3) Gap filling: GapCloser software was finally used to fill the gaps between scaffolds with default parameters, and all the cleaned Illumina reads were used in this step. Paired end reads with one end uniquely mapped to a contig and the other end located in the gap region were used for gap filling.

The statistical results of the genome assemblies are summarized in the **Supplementary Tables 14-20**.

Supplementary Note 7. Draft genome assembly for Nanopore sequenced species

Genomes of three species, including *Psettodes erumei*, *Toxotes chatareus*, and *Polydactylus sextarius*, were sequenced on the Nanopore platform and assembled using the methods described below:

1) Contig assembly: All the cleaned Nanopore long reads were used for genome assembly using WTDBG software (v1.2.8)[4], and all parameters were used as default values except for the "-k, -p, -S, -c".

2) Pilon correction: All the cleaned Illumina short-insert reads were aligned to the assembled contigs using BWA software (BWA-MEM module) (v0.7.12)[5]. The *sam* output file was ranked by the "sort" procedure and the genome index was constructed by the "index" procedure in the SAMtools software (v1.3.1)[6]. Then the contigs were further corrected with two iterations by the short reads using Pilon software (v1.21)[7]. The statistical results of the genome assembly are summarized in **Supplementary Tables 21-23**.

Supplementary Note 8. Chromosome-level assemblies

Genomes of three species, including *Platichthys stellatus*, *Toxotes chatareus*, and *Polydactylus sextarius*, were further assembled into chromosomal level by incorporating Hi-C sequencing data. Briefly, raw sequencing data from Hi-C libraries were filtered using the Hic-Pro software (v3.2)[2]. All remaining paired-end reads that independently and uniquely mapped to the genomes were aligned to the previously assembled contig/scaffold-level genomes using the Juicer software (v1.5)[8], and anchored into chromosomes using the 3D *de novo* assembly (3D-DNA) (v170123) software[9]. Our chromosome construction analysis successfully anchored 24, 24, and 26 chromosomes for *Platichthys stellatus*, *Toxotes chatareus*, and *Polydactylus sextarius*, respectively. The sequence interaction matrices in the three species are shown in **Supplementary Figs. 1-3**, and the statistical results of the chromosome assemblies are summarized in **Supplementary Tables 24-29**.

Supplementary Note 9. Genome size estimation

The genome size was estimated for each species using the kmer method with 17-mer used in this study. The genome size was calculated by the formula: $G = K_{num}/K_{depth}$, where K_{num} represents the total number of kmer, and K_{depth} represents the kmer depth. The genome sizes estimated among species are shown in **Supplementary Fig. 4** and **Supplementary Table 30**.

Supplementary Note 10. Evaluation of assembly quality

1) Assembly quality assessed by BUSCO analysis

We used the Benchmarking Universal Single-Copy Orthologs (BUSCO) software[10] to evaluate the quality of genome assemblies, with both the eukaryota and metazoan databases used in this study. The BUSCO scores for each assembled genome are shown in **Supplementary Tables 31-40**.

2) Assembly quality assessed by the mapping ratio of Illumina reads

The integrity of genome assemblies was further evaluated by the mapping ratio of the sequencing reads to the genome. We aligned the sequencing reads from the short-insert libraries to the assembled genomes using the BWA software (v0.7.12), and the mapped read

numbers and the mapping ratios were calculated respectively for each species. The results showed that over 94% of Illumina reads were successfully mapped to the assembled genome of each species, suggesting good integrity of the genome assemblies in this study (**Supplementary Table 41**).

3) Assembly quality assessed by the mapping ratio of transcripts

The integrity of the assembled genomes was further evaluated by the mapping ratio of the transcripts to the genome. Briefly, all the cleaned RNA-seq reads were assembled into transcripts by Bridger software (r2014-12-01)[11]. These transcripts were further clustered based on pairwise sequence similarity using TGICL software (tgicl_linux)[12], and then assembled by individual clusters to produce longer, more complete consensus sequences. The statistics of transcript assemblies are shown in **Supplementary Tables 42-44**. Transcripts were aligned to the genomes using the BLAT software (v34)[13] with default parameters, and the results of transcript mapping are shown in **Supplementary Tables 45-47**. The results showed that more than 95% transcripts were successfully mapped to the genomes of *Platichthys stellatus*, *Toxotes chatareus*, and *Polydactylus sextarius*, further indicating the fine quality of genome assemblies in this study.

Supplementary Note 11. Genome synteny analysis

Genome synteny analysis was also used to evaluate the genome assembly quality in the three species of *Polydactylus sextarius*, *Toxotes chatareus*, and *Platichthys stellatus*, whose genomes were assembled into the chromosomal level, using the LAST software (v802)[14]. Briefly, genome sequences of the tongue sole (*Cynoglossus semilaevis*, GCF_000523025.1) and the Japanese flounder (*Paralichthys olivaceus*, GCF_001970005.1) were download and used as the reference genomes. "Lastal" command in LAST software[14] with the following parameters: -P 5 -m 100 -E 0.05 were used to align the assembled genomes to the reference genomes to obtain alignment files in the *maf* format. The "maf-swap" command was then used to rank the orders of the alignment results and the best one-to-one aligned blocks were obtained. The synteny relationships between these paired genomes are displayed in circos plots. Our results consistently showed a good genome synteny between the assembled

genomes and reference genomes (**Supplementary Figs. 5-9)**, indicating good quality of the genome assemblies in this study.

Supplementary Note 12. Repetitive sequence annotation

Repetitive sequences in the genomes were identified using different software combinations. Tandem repeats were annotated using the Tandem Repeat Finder software (v4.04)[15] with default parameters. Non-interspersed repeats were searched using the RepeatMasker software (v4.0.6)[16] with default parameters except for "-noint". The transposable elements (TEs) were annotated on both protein and DNA levels. On the protein level, the RepeatProteinMask (RM-BLASTX) was used to search TEs in its protein database. On the DNA level, the RepeatModeler software (v1.0.8, http://www.repeatmasker.org/ RepeatModeler) and the RepeatScout (v1.0.5) software[17] were used to build *de novo* repeat library, and RepeatMasker (v4.0.6) was run against the *de novo* library and repbase (RepBase16.02) separately to identify homologous repeats. The information on annotated repetitive sequences and TEs is summarized in **Supplementary Tables 48-67**.

Supplementary Note 13. Evolution of genome size

Genome sizes varied among flatfish species, as revealed by the genome size estimation. Usually, genome size is largely influenced by the length of repeat sequences in the genome. Therefore, in this study, the detailed compositions of repetitive sequences were analyzed for each assembled genome. In addition, the composition of TEs in each genome was further analyzed to capture the signal of repetitive sequence explosion in the genomes of these species. The results indicated that the repetitive sequences made up a considerable proportion of the flatfish genomes (**Supplementary Table 68**), and that the varied genome sizes among the flatfishes were possibly attributed to the expansion of repetitive sequences (especially for TEs) in the genomes after the divergence of flatfishes (**Supplementary Fig. 10**).

Supplementary Note 14. Protein-coding gene annotation

Protein-coding genes in the genomes were annotated using different annotation strategies: *de novo* prediction, homology-based annotation, and/or transcripts-based annotation. For *de novo*

prediction, the Augustus (v3.2.1)[18] and GENSCAN (v1.0)[19] softwares were used to predict protein-coding genes in the genomes. For homology-based annotation, proteins of seven species (*Mus musculus*, *Gallus gallus*, *Callorhinchus milii*, *Takifugu rubripes*, *Lepisosteus oculatus*, *Cynoglossus semilaevis*, and *Paralichthys olivaceus*) from NCBI and one species (*Danio rerio*) from Ensembl were downloaded. The longest transcript of each gene was selected and any gene with early termination sites was removed. All remaining genes were aligned to the repeat-masked genome for homology-based annotations using *tblastn* with e-value less than 1e-5. The Genewise software (v2.2.0)[20] was used to identify the longest coding regions and/or the highest score in each gene locus to support the presence of a homologous gene. For RNAseq-based annotation, cleaned RNA-seq reads of *Platichthys stellatus*, *Toxotes chatareus*, and *Polydactylus sextarius* were assembled into transcripts using Bridger and aligned against the assembled genomes using BLAT (v34, identity > 90%, coverage > 90%)[13], and PASA (v2.1.0)[21] was then used to link spliced alignments. EvidenceModeler (v1.1.1)[22] was used to integrate the results derived from the different methods into the final protein-coding gene sets. The annotation results are summarized in **Supplementary Table 69**, and the statistics for the annotations are shown in **Extended Data Fig. 1**.

Supplementary Note 15. Functional annotation of protein-coding genes

The functions of these predicted genes were analyzed using the public protein databases. InterProScan (v4.8) was used to screen proteins against databases (Pfam, release 27.0; prints, release 42.0; prosite, release 20.97; ProDom, release 2006.1; smart, release 6.2). In addition, the Kyoto Encyclopedia of Genes and Genomes (KEGG), NR, SwissProt (Release 2011.6), and TrEMBL (Release 2011.6) databases were also searched for homology-based functional assignments using the BLAST software (v2.6.0) with e-value of 1e-5. Results of the annotations are summarized in **Supplementary Tables 70-79**.

Supplementary Note 16. Gene family analysis

The ortholog/paralog genes were identified in the assembled genomes of *Trinectes maculatus*, *Chascanopsetta lugubris*, *Brachirus orientalis*, *Paraplagusia blochii*, *Colistium nudipinnis*,

Pseudorhombus dupliocellatus, *Platichthys stellatus*, *Psettodes erumei*, *Polydactylus sextarius*, and *Toxotes chatareus*, along with the species (*Cynoglossus semilaevis*, *Paralichthys olivaceus*, *Scophthatmus maximus*, *Danio rerio*, *Larimichthys crocea*, *Labrus bergylta*, *Oreochromis niloticus*, and *Oryzias latipes*) with published genome using the OrthoMCL pipeline (v2.0.9)[23]. Briefly, all the protein-coding genes of the published species were downloaded from the NCBI database except for *Scophthatmus maximus*, which was downloaded from its own website (http://denovo.cnag.cat/genomes/turbot). To improve the accuracy of the analysis, genes that have shorter than 30 amino acids or have early stop codons in the coding regions were removed. All the remaining genes were used for the identification of orthologs/paralogs using the OrthoMCL software. A total of 1,693 single copy genes were identified among all the species analyzed, and the results are shown in **Supplementary Fig. 11**.

Supplementary Note 17. Phylogenetic tree and ancestral chromosome construction

The single-copy genes identified among the species (*Trinectes maculatus*, *Chascanopsetta lugubris*, *Brachirus orientalis*, *Paraplagusia blochii*, *Colistium nudipinnis*, *Pseudorhombus dupliocellatus*, *Platichthys stellatus*, *Psettodes erumei*, *Polydactylus sextarius*, *Toxotes chatareus*, *Cynoglossus semilaevis*, *Paralichthys olivaceus*, *Scophthatmus maximus*, *Danio rerio*, *Larimichthys crocea*, *Labrus bergylta*, *Oreochromis niloticus*, and *Oryzias latipes*) were aligned using the MUSCLE software (v3.8.31)[24] and concatenated into super-genes for phylogenetic relationship analyses. The maximum likelihood-based phylogenetic analysis was conducted through RAxML (v8.2.9)[25] using several datasets, including concatenated sequences of CDS (Codon1+2+3, GTRGAMMA model), CDS (Codon1+2, GTRGAMMA model) and 4dTV (fourfold degenerate synonymous site, GTRGAMMA model) with zebrafish (*Danio rerio*) as the outgroup. Additionally, the simulation tree was constructed by DensiTree (v2.21)[26] using the single-copy genes derived from the gene family analysis. Moreover, the species tree of these species was also constructed using MPEST (v2.0)[27] and OrthoFinder (v2.3.5)[28]. The divergence time of species were then estimated based on the 4dTV sequences through the Bayesian relaxed molecular clock approach using the MCMCtree program in PAML package (v4.8)[29]. The fossil records downloaded from

TIMETREE website (http://www.timetree.org) were used for calibrating the divergence times. Our results consistently revealed a non-monophyletic origin of flatfishes, with *Psettodes erumei* of suborder Psettodoidei forming one clade with the Perciformes species of *Toxotes chatareus* and *Polydactylus sextarius*, and the species of suborder Pleuronectoidei (including *Trinectes maculatus*, *Chascanopsetta lugubris*, *Brachirus orientalis*, *Paraplagusia blochii*, *Colistium nudipinnis*, *Pseudorhombus dupliocellatus*, *Platichthys stellatus*, *Cynoglossus semilaevis*, *Paralichthys olivaceus*, and *Scophthatmus maximus*) forming the sister clade in the phylogenetic tree (**Supplementary Figs. 12-17**). With the fossil calibration, we deduced the divergence times and found that the first emergence of the Pleuronectoidei and Psettodoidei could retrospect to about 76.1 and 80.0 million years ago (Ma), respectively at late Cretaceous (**Fig. 2a in main text**).

To provide more evidence for the non-monophyly of flatfishes, We further looked at some denoted body plan genes in the lineage of *Toxotes chatareus*, *Polydactylus sextarius* and *Psettodes erumei*, we observed majority of body plan genes in T*oxotes chatareus* and *Polydactylus sextarius* have the same mutations with perciformes instead of real flatfishes (Pleuronectoidei) (**Supplementary Table 80**), further supporting the non-monophyletic origin of flatfishes. In addition, we further reconstructed the ancestral chromosomes for the Pleuronectoidei and Psettodoidei lineages to confirm the non-monophyletic origin of flatfishes. Briefly, the chromosome level genomic data of *Platichthys stellatus*, *Cynoglossus semilaevis* in real flatfish Pleuronectoidei lineage, and *Toxotes chatareus* and *Polydactylus sextarius* leading to the flatfish-like Psettodoidei lineage, were aligned and the genome synteny were analyzed using LAST[14] with the parameters of –k 1 -m 10 –E 0.05. Then the chromosome variation events within and between lineages were compared using ANGES (v1.01)[30] to detect the lineage specific chromosome variation. The results shown that there is no shared fission or fusion events were identified in these two groups in our analysis. Then the contig sequences obtained from Nanopore reads of *Psettodes erumei* was used to check for these lineage specific chromosome fusion and fission events to further test if flatfish-like Psettodoidei lineage (including *Psettodes erumei*) has different ancestral chromosomes from that of real flatfish Pleuronectoidei. We identified one contig sequence read through a fission event in real flatfish Pleuronectoidei lineage, further suggesting the independent origin of real

flatfish Pleuronectoidei lineage and flatfish-like Psettodoidei lineage. In order to capture real evolutionary signals, we therefore split the previously known Pleuronectiformes into "real flatfish Pleuronectoidei (RFP)" and "flatfish-like Psettodoidei (FLP)" lineages in the following analyses.

Supplementary Note 18. Relative evolutionary rate analysis

The relative evolutionary rates of each branch were calculated using two-cluster analysis and Tajima's relative rate test in the LINTRE[31] and MEGA softwares[32], respectively. (a) Two-cluster analysis: two-cluster analysis tests molecular evolution of multiple sequences in a phylogenetic context. A faster or slower evolutionary rate of particular taxa was analyzed using Z-statistics and *tpcv* module in the LINTRE program. (b) Tajima's relative rate test: a higher number of lineage-specific substitutions indicate a much faster evolutionary rate based on Chi-square test. All the single-copy genes were used in these two analyses with the zebrafish as the outgroup species. Our results revealed much faster evolutionary rates among Pleuronectoidei species (*Trinectes maculatus*, *Chascanopsetta lugubris*, *Brachirus orientalis*, *Paraplagusia blochii*, *Colistium nudipinnis*, *Pseudorhombus dupliocellatus*, *Platichthys stellatus*, *Cynoglossus semilaevis*, *Paralichthys olivaceus*, and *Scophthatmus maximus*) than Perciformes species (*Polydactylus sextarius*, *Toxotes chatareus*, *Larimichthys crocea*, *Labrus bergylta*, and *Oreochromis niloticus*) (**Supplementary Tables 81 and 82**). The evolutionary rate in flatfish-like *Psettodes erumei* was only slightly faster than the Perciformes species (**Supplementary Tables 81 and 82**), which may explain why they exhibits a "simply an asymmetric percoid" phenotype.

Supplementary Note 19. Expansion and contraction of gene families

To identify the gene families that have experienced expansion or contraction in flatfishes, data of the annotated gene families and evaluated divergence time among flatfishes and other closely related species were used and analyzed using the CAFE software (v3.1)[33] to infer changes in gene family size using a probabilistic model. The potential expansion and contraction of gene families in the ancestral node of real flatfish Pleuronectoidei and flatfish-like Psettodoidei node were separately analyzed. Our results revealed 2 expanded and

51 contracted gene families (P value < 0.05) in the ancestor node of real flatfish Pleuronectoidei, and 43 expanded and 168 contracted gene families (P value < 0.05) in the node of flatfish-like Psettodoidei. The observed gene number in some gene families, such as BMP, FGF, HOX, NOTCH, and WNT, were counted and shown in **Extended Data Fig. 2**. GO and KEGG enrichment analyses of these expanded and contracted gene families in both real flatfish Pleuronectoidei species and flatfish-like *Psettodes erumei* were performed using EnrichGO and Rscript[34], and the results are shown in **Supplementary Tables 83-89**.

Supplementary Note 20. Identification of positively selected and rapidly evolving genes

All the one-to-one orthologous genes were extracted from species and used to identify positively selected or rapidly evolving genes. Multiple sequence alignments were generated using the MUSCLE software (v3.8.31) with default parameters. These high quality alignments were used to estimate three types of ω (the ratio of the rate of non-synonymous substitutions to the rate of synonymous substitutions) using branch and branch-site models in the *codeml* program of the PAML package (v4.8)[29]. Branch model (model=2, NSsites=0) was used to detect the ω of appointed branch to test (ω0) and average ω of all the other branches (ω1) and branch-site models (model=0, NSsites=0) were used to estimate the average of whole branches (ω2). Then $\chi 2$-test was used to check whether ω2 was significantly higher than ω1 and ω0 with the threshold P value < 0.05, which hinted that these genes could be under positive selection or fast evolution. In order to capture real evolutionary signals, the positively selected or rapidly evolving genes in real flatfish Pleuronectoidei species and flatfish-like *Psettodes erumei* were identified separately, using *Danio rerio*, *Larimichthys crocea*, *Labrus bergylta*, *Oreochromis niloticus*, and *Oryzias latipes* as the outgroups. Our results revealed 48 genes in real flatfish Pleuronectoidei species, such as *bbox1* (**Supplementary Fig. 18**), *pou2f1* (**Supplementary Fig. 19**), *tpbg* (**Supplementary Fig. 20**), and 303 genes in flatfish-like *Psettodes erumei* that were positively selected or rapidly evolving, compared to the outgroup species (**Supplementary Tables 90 and 91**). In addition, GO enrichment analysis of these positively selected and rapidly evolving genes in both real flatfish Pleuronectoidei species and flatfish-like *Psettodes erumei* was performed using EnrichGO, and KEGG enrichment analysis was performed using Rscripts. Results of GO and

KEGG enrichment analyses are shown in **Supplementary Tables 92-95**.

Supplementary Note 21. Identification of lineage-specific mutations

The high quality alignments of amino acid (aa) generated from the MUSCLE software were used to identify lineage-specific mutated genes. All the single copy genes among species were manually checked and any genes that had the same variation in any particular taxon, compared with outgroup species, were identified as the candidate lineage-specific mutated genes (LSGs). Candidate LSGs were further double-checked using original Illumina reads to avoid the assembly and sequencing errors. In addition, Bayesian ancestral state inference was further conducted to validate the potential LSGs using the *codeml* program in PAML software (v4.8)[29]. In the Bayesian framework, the ancestral state was inferred by the state with the highest posterior probability. In our case, only the ancestral state of real flatfish Pleuronectoidei species was different from the ancestor of all the Pleuronectiformes species, *Toxotes chatareus*, and *Polydactylus sextarius*, the candidate LSGs were recognized as the true Pleuronectoidei lineage-specific mutated genes. Our results revealed 6 genes, such as *sfrp5* (**Supplementary Figs. 21 and 22**), that underwent true lineage-specific mutation in real flatfish Pleuronectoidei species (**Supplementary Table 96**), whereas 15 genes underwent the same mutation in all flatfishes compared with the outgroup species (**Supplementary Table 97**).

Supplementary Note 22. Identification of conserved non-coding elements

Using the *Platichthys stellatus* genome as the reference, genomes of the other species were aligned to the reference genome using the LAST software (v802)[14] with parameters: -P 5 -m 100 -E 0.05. The generated alignments were checked loci by loci, and loci that were present in more than eight real flatfish Pleuronectoidei species but not present in any non-flatfish species were recognized as the candidate real flatfish Pleuronectoidei-specific conserved non-coding elements (SCNEs). Any SCNEs sequence less than 20 bp were removed to ensure the accuracy of identification. Since gene regulatory elements usually locate in the conserved non-coding regions, we downloaded the transcription factor binding sites (TFBS, Human) from the UCSC database that is organized by the ENCODE project. A total of 2,750,490

TFBS terms were downloaded and SCNEs sequences were then aligned to these TFBS data with BLAST (e=1e-5). Altogether, 98.33% of the CNEs sequences could be aligned to the TFBS data with 349,335 bp SCNEs were located in intron regions, 100,756 bp SCNEs in up-stream regions, 37,343 bp SCNEs in the down-stream regions and 74,041 bp SCNEs in intergenic regions. The top genes with SCNEs were then used for functional enrichment analyses, and the results are shown in **Supplementary Fig. 23** and **Supplementary Tables 98 and 99**.

Supplementary Note 23. Genes associated with benthic adaptation and body plan creation

Genes undergoing significant alterations in flatfishes revealed by our comparative genomic analysis may suggest their possible roles in evolution of specialized body plan of flatfishes. Through extensive bioinformatic analyses, we observed that the enrichment categories of top candidate genes under significant alteration in both real flatfish Pleuronectoidei (RFP) and flatfish like Psettodoidei (FLP) are associated with visual perception (*dmbx1a* and *opn3* in RFP versus *cryba4* and *opn3* in FLP), immune response (*bahd1*, *ripk1*, and *pik3ip1* in RFP versus *nfkbid*, *trim59*, and *themis2* in FLP), hypoxia tolerance (*fbxl5* in RFP versus *ucp2* in FLP), and cardiac function (*tmem43*, *dis3l1*, *popdc2*, and *glrx1* in RFP versus *irx4a* and *glrx*3 in FLP), possibly suggesting a similar remodeling of their visual, immune, respiratory and circulatory systems in benthic adaptation to seafloor colonization (**Extended Data Fig. 3**). Enriched categories of the remaining top candidate genes under significant alterations in RFP and FLP were associated with axial patterning, neural patterning, musculoskeletal restructuring, lipid deposition, and fin cartilage reorganization, suggesting their roles in new body plan evolution and adaptation after seafloor colonization.

Supplementary Note 24. Measurement of body morphology and fat content

Three flatfish species, *Cynoglossus semilaevis*, *Paralichthys olivaceus* and *Brachirus orientalis*, and three non-flatfish teleosts, *Polydactylus sextarius*, *Larimichthys crocea* and *Oryzias latipes*, were collected in the wild and used to characterize phenotypical features and fat content of flatfishes relative to non-flatfish species. Briefly, three individuals (sex was not

determined) in adult for each species were collected in December of 2018 to avoid breeding season when the morphs and fat content would be affected. The body flatness was measured and represented by the relative ratio of maximum height of dorsal-ventral axis to maximum length of left-right axis, and by the ratio of maximum length of left-right axis to the total length of fish. The fat content in both the muscle tissues and the whole fish were also determined in flatfishes relative to non-flatfish species in order to test whether their flat body plan arises from reduced lipid accumulation. The fat content was analyzed in three individuals for each species using the methods described by AOAC (Association of Official Analytical Chemists International)[35]. Briefly, before lipid extraction, all tissues were dried and weighted to the nearest 0.1 μg on a XP6 analytical balance (Metler-Toledo, Viroflay, France). Lipids were extracted in a Soxtec system (Soxtec System HT6, Tecator, Haganas, Sweden) with diethyl ether as extraction liquid. The extracts were dried in an oven (Selecta, Spain) at 104 °C, until the weight became constant. The distinction of body morphology and fat content of flatfishes compared to non-flatfish teleosts was tested using Student's t test with two tails, which was implemented in SPSS19.01. Our results revealed that the body plan flatness could be readily observed in flatfishes compared to non-flatfishes species (**Supplementary Fig. 24**). The relative length ratios of the maximum dorsal-ventral axis to maximum left-right axis in flatfishes were much significantly higher than non-flatfishes species (P value = 7.64×10^{-5}) (**Fig. 3a in main text**). Whereas, the relative length ratios of the maximum left-right axis to the body length in flatfishes were also much significantly lower than non-flatfishes species (P value = 2.99×10^{-8}, **Extended Data Fig. 4**). In addition, average of 6.22 and 5.76 folds significantly lower fat content (P value = 2.81×10^{-5}; P value = 3.10×10^{-5}) were respectively observed in the whole body and muscular tissues of flatfishes compared to other non-flatfishes species (*Polydactylus sextarius*, *Larimichthys crocea*, and *Oryzias latipes*) (**Fig. 3e in main text**), indicating a "lean" phenotype possibly have implications in evolution of their body plan flatness.

Supplementary Note 25. Catalytic activity assay of enzymes

In vitro enzyme activity assay was used to test the functional consequence of RFP-specific mutation in bbox1 and rdh14 proteins. RFP specific genes and that of the outgroups were

codon-optimized according to the *Escherichia coli* preference and then synthesized and cloned into the vector pET-28a by Wuhan GeneCreate Biological Engineering Co., Ltd. (Wuhan, China). Recombinant *E. coli* BL21 cells were grown in 2YT-broth (yeast extract 10 g/L, tryptone 16 g/L, and NaCl 5 g/L) containing 50 mg/L kanamycin at 37°C under good aeration. Induction of 1a-hydroxylase cDNA transcription was initiated by addition of isopropylthio-β-D-thiogalactoside (IPTG) and the protein was then extracted using a constant cell disruption system, and was purified using Ni^{2+} affinity columns and stored until use. The bbox1 activity was then analyzed following the procedure described by Cao et al[36] and Ling[37], and rdh14 activity assay was measured according to protocol described by Rattner et al[38]. Each experiment was performed with three reaction replicates to determine the mean ± SD of the catalytic activity value of the enzymes and the distinction of enzyme catalytic activity of flatfishes compared to non-flatfish teleosts was tested using Student's *t-test* with two tails. The results showed that compared with the outgroups, RFP-specific rdh14 has significantly lower (2.51-fold low; P value = 2.84×10^{-6}) activity catalyzing retinaldehyde into retinol, whereas RFP-specific bbox1 has significantly higher (1.3-fold high; P value = 2.76×10^{-3}) catalytic activity transforming gamma-butyrobetaine into L-carnitine, suggesting differed enzyme catalyzing efficiency in rdh14 and bbox1 between the two groups. These results indicate that RFP-specific mutations in both bbox1 and rdh14 genes have expected functional effects. The result was shown in **Fig. 3d** of the main text and in **Extended Data Fig. 5**.

Supplementary Note 26. Sample collection in metamorphic flounder

We collected tissues from pre-metamorphic larvae (16 days after fertilization), pro-metamorphic larvae (22 days after fertilization), metamorphic climax larvae (29 days after fertilization), and post-metamorphic larvae (37 days after fertilization) of Japanese flounders (*Paralichthys olivaceus*) (**Supplementary Fig. 25**) reared under artificial environments. The eye, skin, and muscle tissues were sampled in three biological replicates from both sides of the metamorphosing larvae in each metamorphic time windows. Since the metamorphic flounder larva is too small and tissue samples from one individual is far from enough for a regular transcriptome analysis, we actually dissected muscle, eyes and skin from both sides of at least 30 individual larvae and then respectively pooled each type of tissues

together to conduct RNA-seq. We repeated three times from different batch of larvae for the sample collection as three biological replicates. All the samples were stored in liquid nitrogen until use in laboratory (**Supplementary Table 100**).

Supplementary Note 27. Gene expression profiles in metamorphic flounders

RNA extracted from the eyes, skins and muscle tissues across the left-right axis in different metamorphic time windows (pre-metamorphic larva, pro-metamorphic larva, metamorphic climax larva, and post-metamorphic larva) of *Paralichthys olivaceus* were sequenced on the Illumina sequencing platform. The cleaned RNA-seq reads were then aligned to the reference genome using Tophat2 (v2.1.1)[39], and transcriptome assembly was performed using the cufflinks software (v2.2.1)[40]. Gene expression profiles in each tissue were measured in FPKM (fragments per kilo-base of exons per million reads mapped) values using the Tophat-cufflinks pipeline. PCA analysis was further performed to validate the sample collection based on gene expression profiles, which indicate a proper sample collection in our study (**Supplementary Fig. 26**). Further, highly expressed genes were identified using *Tau* method[41], and the identified genes were further used for functional enrichment analyses. Results of the functional enrichment analyses are shown in **Supplementary Tables 101-124**. The results showed that multiple genes in both RA (*aldh1*, *aldh8*, *rdh5*, *rdh7*, *rdh8*, *rdh11*, *rdh12*, *rdh13*) and WNT (*wnt1*, *wnt4*, and *wnt10*) signal pathways exhibited obvious transient expression fluctuations in all three examined flounder tissues (eyes, muscles, and skins) during metamorphosis, with marked left-right asymmetrical expression (both in gene expression level and in specific highly expressed gene number) initiating from pre-metamorphic stage, climbing to asymmetrical climax during pro-metamorphic and metamorphic climax stage, and then recovering to symmetry in post-metamorphic stage (**Figs. 5c-e, Extended Data Fig. 6)**. The asymmetrical expression of these genes observed during metamorphosis may indicate their roles in the eye migration, cranium deformation, and lopsided pigmentation during metamorphosis.

Supplementary Note 28. Real-time quantitative PCR

The beta-actin gene was selected and used as an internal control of the q-PCR experiment. Each experiment was performed at least three biological replicates and the comparative cycle threshold was used to present a fold change for each assay. The left-right distinction of gene expression profiles in each metamorphic time window was tested using Student's *t-test* with two tails. We randomly chose 6 genes showing asymmetrical expression for this experiment. Considering their expression patterns and our sample availability, five of them (*wnt10a*, *aldh4a1*, *aldh9a1*, *mitf*, and *tyro*) were tested in skin, and one (*pitx2*) was tested in muscle. The results show that the four WNT, RA and NODAL signaling genes (*wnt10a*, *aldh4a1*, *aldh9a1*, and *pitx2*) exhibit obvious transient expression fluctuations in corresponding tissues, with marked left-right asymmetrical expression initiating from pre-metamorphic stage, climbing to asymmetrical climax during pro-metamorphic and metamorphic climax stage, and then recovering to symmetry in post-metamorphic stage, which is consistent with what was observed in our transcriptome analysis. In addition, genes associated with pigmentation (*mitf* and *tyro*) showed maximal asymmetrical expression a little bit later after the asymmetrical expression of RA and WNT signals in the skin, showing potential links between RA, WNT signaling and asymmetrical pigmentation in flatfishes. The results were shown in the **Extended Data Fig. 7**.

Supplementary Note 29. Measurement of the fin length

Three flatfish species, *Cynoglossus semilaevis*, *Paralichthys olivaceus* and *Brachirus orientalis*, and three non-flatfish teleosts, *Polydactylus sextarius*, *Larimichthys crocea* and *Oryzias latipes*, were collected in the wild and measured for the relative lengths of dorsal, anal, pectoral and pelvic fins in flatfishes compared to non-flatfish species. Three individuals of each species were measured and the relative fin lengths were represented as the ratios of dorsal, anal, pectoral and pelvic fins length to the total length of the fish. The distinction of fin morphology of flatfishes compared to non-flatfish teleosts was tested using Student's *t-test* (two tails). The results showed that the dorsal and anal fins were significantly elongated in flatfishes compared to non-flatfish species, whereas the pectoral and pelvic fins were significantly reduced in flatfishes compared to non-flatfish species (P value < 0.001; **Extended Data Figs. 8 and 9; Supplementary Fig. 27)**.

References

1. Sambrook, J., Fritsch, E.F. & Maniatis, T. *Molecular Cloning: a Laboratory Manual, second ed*. (Cold Spring Harbor Laboratory Press, Cold Spring Harbor, NY, America, 1989).
2. Servant, N. *et al.* HiC-Pro: an optimized and flexible pipeline for Hi-C data processing. *Genome Biology* **16**: 259 (2015).
3. Kajitani, R. *et al.* Efficient de novo assembly of highly heterozygous genomes from whole-genome shotgun short reads. *Genome Res*. 24, 1384-1395 (2014).
4. Ruan, J. & Li, H. Fast and accurate long-read assembly with wtdbg2. *Nat. Methods*. 17, 155-158 (2019).
5. Li, H. & Richard, D.J.B. Fast and accurate short read alignment with Burrows–Wheeler transform. Bioinformatics 25, 1754-1760 (2009).
6. Li, H. *et al.* The Sequence Alignment/Map format and SAMtools. *Bioinformatics* **25**, 2078-2079 (2009).
7. Walker, B.J. *et al.* Pilon: An integrated tool for comprehensive microbial variant detection and genome assembly improvement. *Plos One* **9**: e112963 (2014).
8. Durand, N.C. *et al.* Juicer provides a one-click system for analyzing loop- resolution Hi-C experiments. *Cell Syst.* **3**, 95-98 (2016).
9. Dudchenko, O. *et al.* De novo assembly of the Aedes aegypti genome using Hi-C yields chromosome-length scaffolds. *Science* **356**, 92-95 (2017).
10. Simão, F.A., Waterhouse, R.M., Panagiotis, I., Kriventseva, E.V. & Zdobnov, E.M. BUSCO: assessing genome assembly and annotation completeness with single-copy orthologs. *Bioinformatics* **31**, 3210-3212 (2015).
11. Chang, Z. *et al.* Bridger: a new framework for de novo transcriptome assembly using RNA-seq data. *Genome Biol.* **16**: 30 (2015).
12. Pertea, G. *et al.* TIGR Gene Indices clustering tools (TGICL): a software system for fast clustering of large EST datasets. *Bioinformatics* **19**, 651-652 (2003).
13. Kent, W.J. BLAT-The BLAST-like alignment tool. *Genome Res.* **12**, 656-664 (2002).
14. Kielbasa, S.M., Wan, R., Sato, K., Horton, P. & Frith, M.C. Adaptive seeds tame genomic sequence comparison. *Genome Res.* **21**, 487-493 (2011).
15. Benson, G. Tandem repeats finder: a program to analyze DNA sequences. *Nucleic Acids Res.* **27**, 573-580 (1999).
16. Bedell, J.A., Korf, I. & Gish, W. MaskerAid: a performance enhancement to RepeatMasker. *Bioinformatics* **16**, 1040-1041 (2000).
17. Price, A.L., Jones, N.C. & Pevzner, P.A. De novo identification of repeat families in large genomes. *Bioinformatics* **21**, 351-358 (2005).
18. Stanke, M. & Waack, S. Gene prediction with a hidden Markov model and a new intron submodel. *Bioinformatics* **19**, 215-225 (2003).
19. Burge, C. & Karlin, S. Prediction of complete gene structures in human genomic DNA. *J. Mol. Biol.* **268**, 78-94 (1997).
20. Birney, E., Clamp, M. & Durbin, R. GeneWise and genomewise. *Genome Res.* **14**, 988-995 (2004).
21. Haas, B.J. *et al.* Improving the Arabidopsis genome annotation using maximal

transcript alignment assemblies. *Nucleic Acids Res.* **31**, 5654-5666 (2003).
22. Haas, B.J. *et al.* Automated eukaryotic gene structure annotation using EVidenceModeler and the program to assemble spliced alignments. *Genome Biol.* **9**: 7 (2008).
23. Li, L., Stoeckert, C.J. & Roos, D.S. OrthoMCL: Identification of ortholog groups for eukaryotic genomes. *Genome Res.* **13**, 2178-2189 (2003).
24. Edgar, R.C.MUSCLE: multiple sequence alignment with high accuracy and high throughput. *Nucleic Acids Res.* **32**, 1792-1797 (2004).
25. Stamatakis, A. RAxML version 8: a tool for phylogenetic analysis and post-analysis of large phylogenies. *Bioinformatics* **30**, 1312-1313 (2014).
26. Bouckaert, R.R. DensiTree: making sense of sets of phylogenetic trees. *Bioinformatics* **26**, 1372-1373 (2010).
27. Liu, L.A., Yu, L.L. & Edwards, S.V. A maximum pseudo-likelihood approach for estimating species trees under the coalescent model. *Bmc Evol. Biol.* **10** (2010)..
28. Emms, D.M. & Kelly, S. OrthoFinder: phylogenetic orthology inference for comparative genomics. *Genome Biol.* **20**: 38 (2019).
29. Yang, Z.H. PAML 4: Phylogenetic analysis by maximum likelihood. *Mol. Biol. Evol.* **24**, 1586-1591 (2007).
30. Jones, B.R., Rajaraman, A., annier, E., and Chauve, T.C. ANGES: reconstructing ANcestral GEnomeS maps. *Bioinformatics* **28**, 2388-2390 (2012).
31. Takezaki, N., Rzhetsky, A. & Nei, M. Phylogenetic Test of the Molecular Clock and Linearized Trees. *Mol.Biol.Evol.* **12**, 823-833 (1995).
32. Kumar, S., Stecher, G. & Tamura, K. MEGA7: Molecular Evolutionary Genetics Analysis Version 7.0 for Bigger Datasets. *Mol. Biol. Evol.* **33**, 1870-1874 (2016).
33. De Bie, T., Cristianini, N., Demuth, J.P. & Hahn, M.W. CAFE: a computational tool for the study of gene family evolution. *Bioinformatics* **22**, 1269-1271 (2006).
34. Huang, D.W., Sherman, B.T. & Lempicki, R.A. Bioinformatics enrichment tools: Paths toward the comprehensive functional analysis of large gene lists. *Nucleic Acids Res.* **37**, 1-13 (2009).
35. Cunniff, P.A. *Official Methods of Analysis of AOAC International.16th edition,* (AOAC International, Gaithersburg, USA, 1997).
36. Cao, Q., Ren, S., Park, M., Choi, Y. & Lee, B. Determination of highly soluble L-carnitine in biological samples by reverse phase high performance liquid chromatography with fluorescent derivatization. *Arch. Pharm. Res.* **30**, 1041-1046 (2007).
37. Ling, B., Aziz, C. & Alcorn, J. Systematic Evaluation of Key L-Carnitine Homeostasis Mechanisms during Postnatal Development in Rat. *Nutrition & Metabolism* **9**, 1-10 (2012).
38. Rattner, A., Smallwood, P. & Nathans, J. Identification and characterization of all-trans-retinol dehydrogenase from photoreceptor outer segments, the visual cycle enzyme that reduces all-trans-retinal to all-trans-retinol. *J. Biol. Chem.* **275**, 11034-11043 (2000).
39. Kim, D. *et al.* TopHat2: accurate alignment of transcriptomes in the presence of insertions, deletions and gene fusions. *Genome Biol.* **14**: 36 (2013).

40. Trapnell, C. *et al.* Transcript assembly and quantification by RNA-Seq reveals unannotated transcripts and isoform switching during cell differentiation. *Nat. Biotechnol.* **28**, 511-515 (2010).
41. Kryuchkova-Mostacci, N. & Robinson-Rechavi, M. A benchmark of gene expression tissue-specificity metrics. *Brief. Bioinform*. **18**, 205-214 (2017).

Supplementary Figures

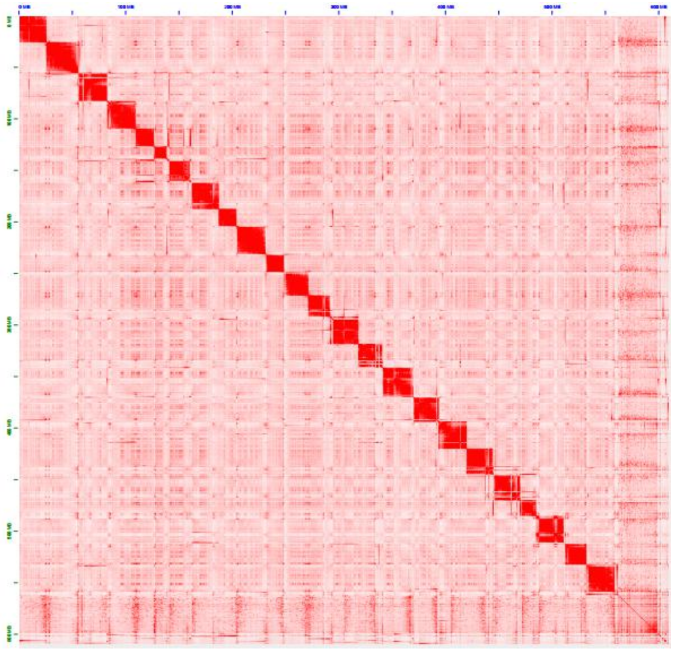

Supplementary Fig. 1. Chromosomal interactions in *Platichthys stellatus*.

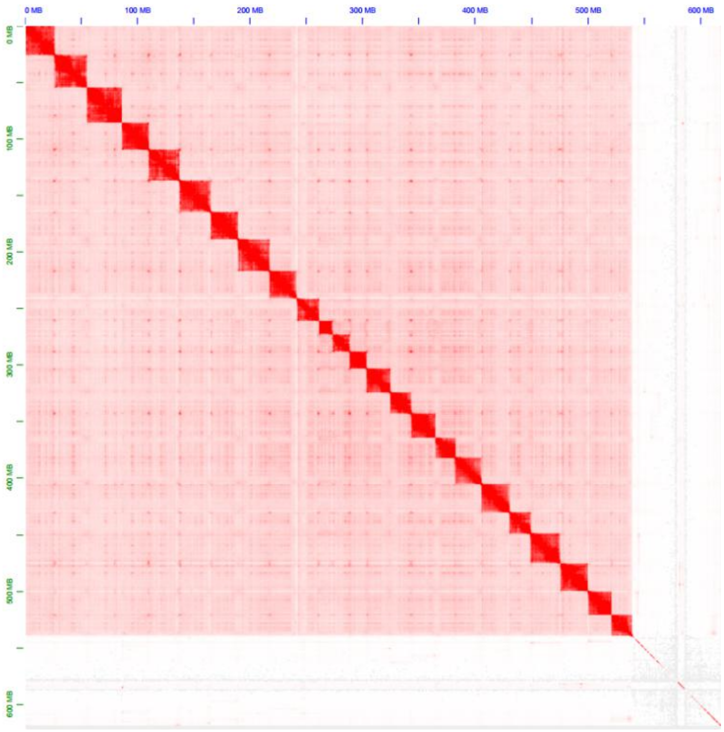

Supplementary Fig. 2. Chromosomal interaction in *Toxotes chatareus*.

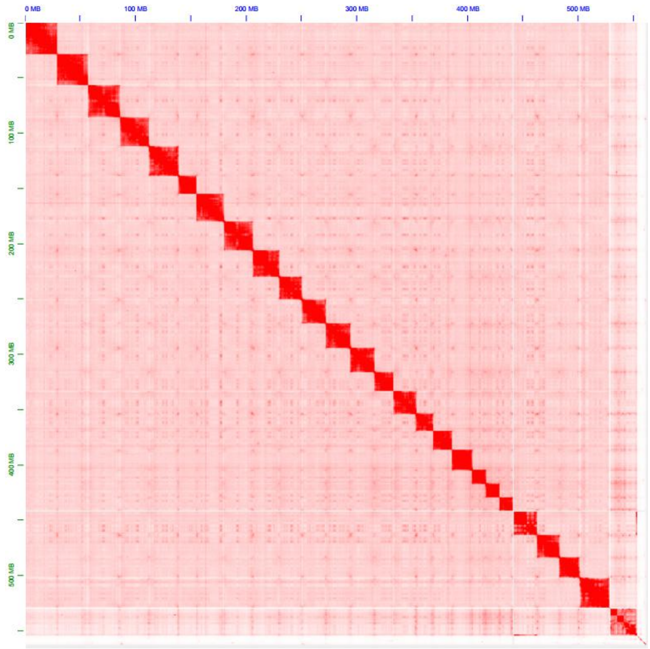

Supplementary Fig. 3. Chromosomal interaction in *Polydactylus sextarius*.

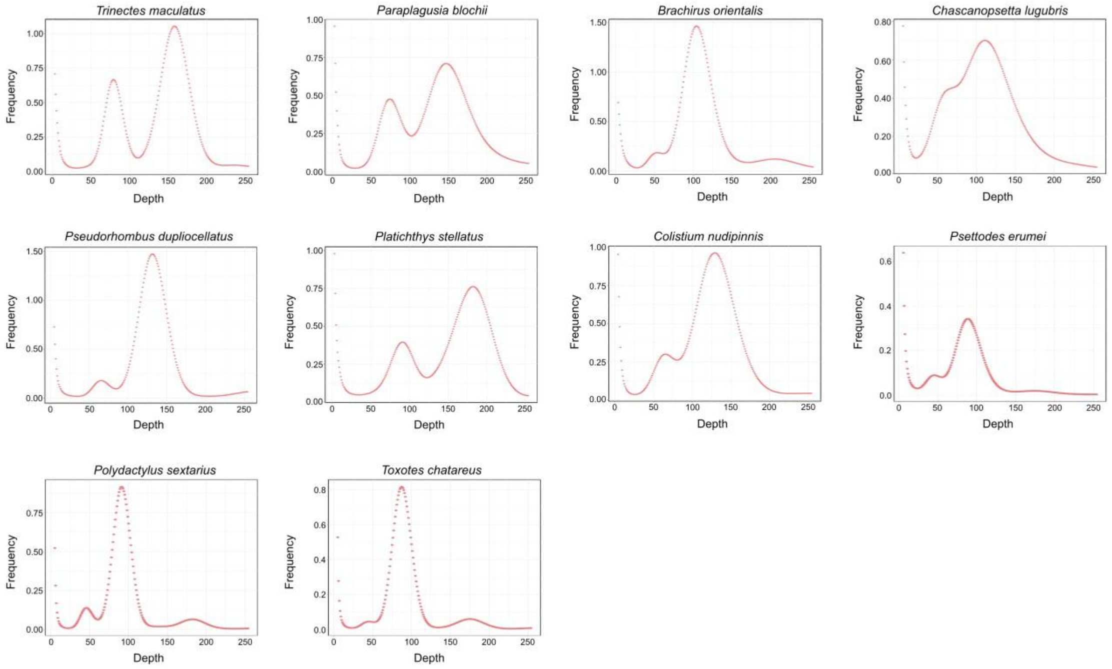

Supplementary Fig. 4. Kmer distribution for each species. In each diagram, the x-axis represents the depth of kmer, and the y-axis represents the frequency of the kmer. The main peak was shown and used for the estimation of genome size of each species, and the small peak near the main was the heterozygous peak.

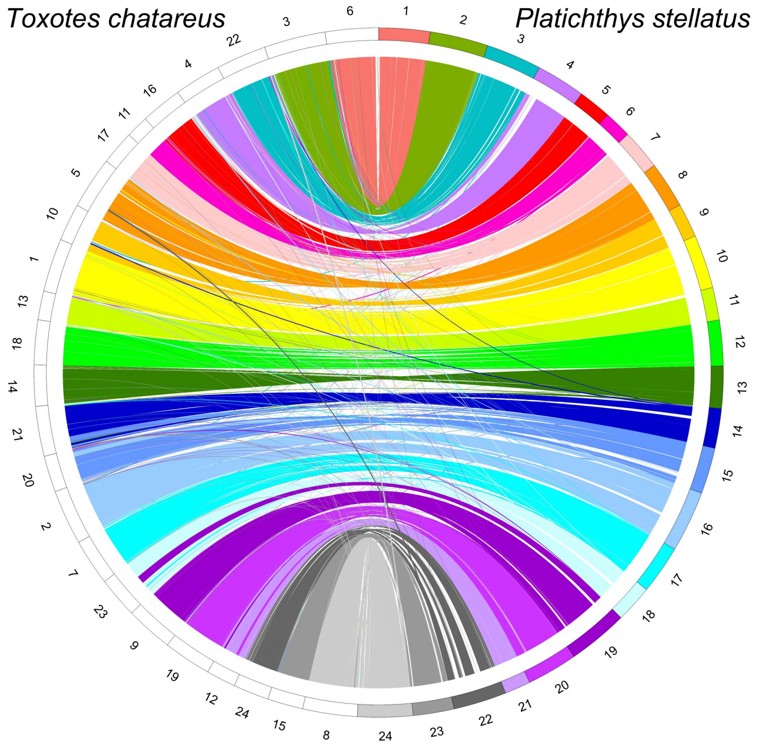

Supplementary Fig. 5. Synteny between the *Platichthys stellatus* and *Toxotes chatareus* genomes.

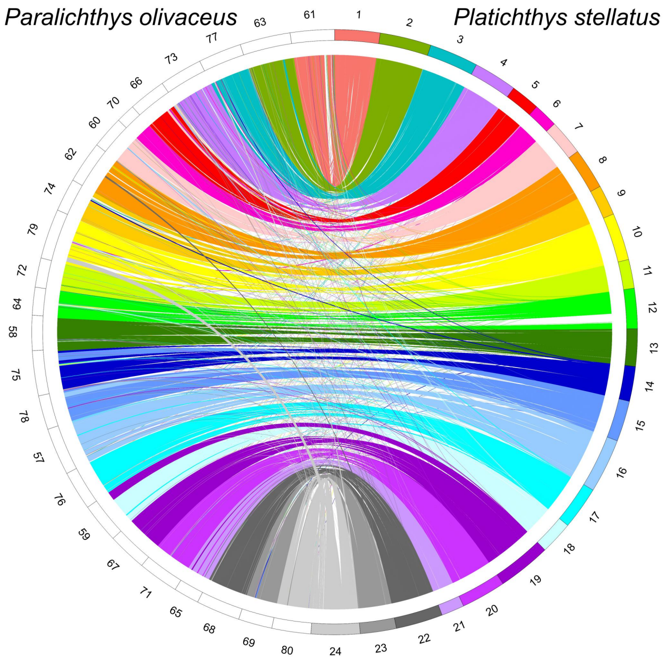

Supplementary Fig. 6. Synteny between the *Platichthys stellatus* and *Paralichthys olivaceus* genomes.

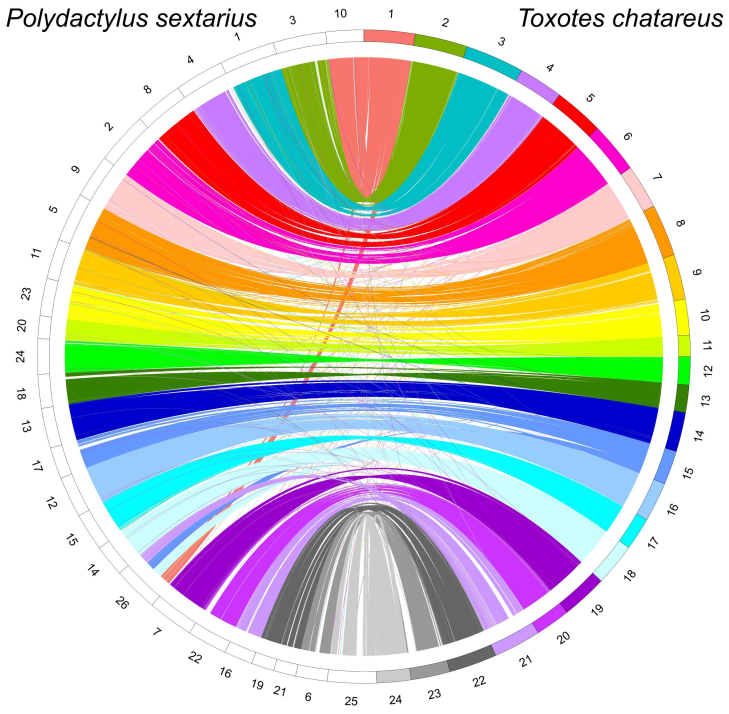

Supplementary Fig. 7. Synteny between the *Polydactylus sextarius* and *Toxotes chatareus* genomes.

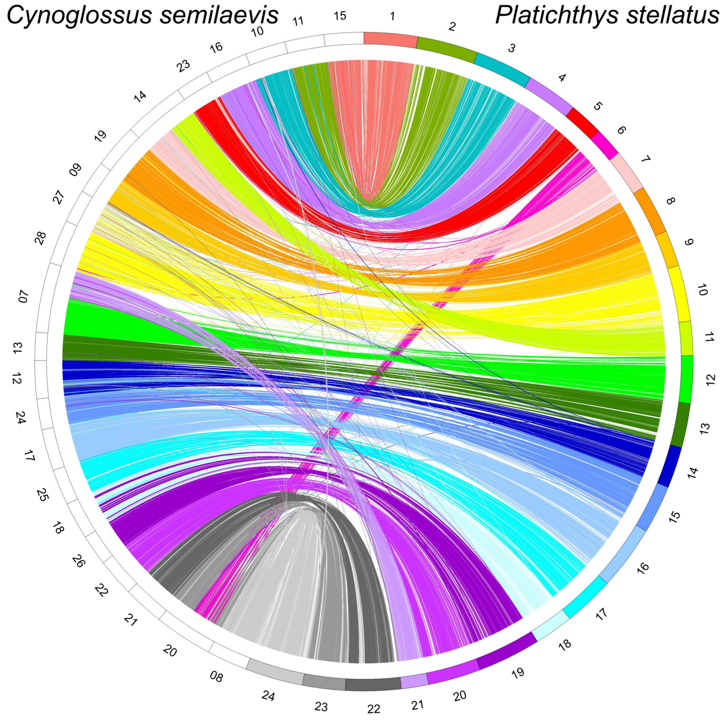

Supplementary Fig. 8. Synteny between the *Platichthys stellatus* and *Cynoglossus semilaevis* genomes.

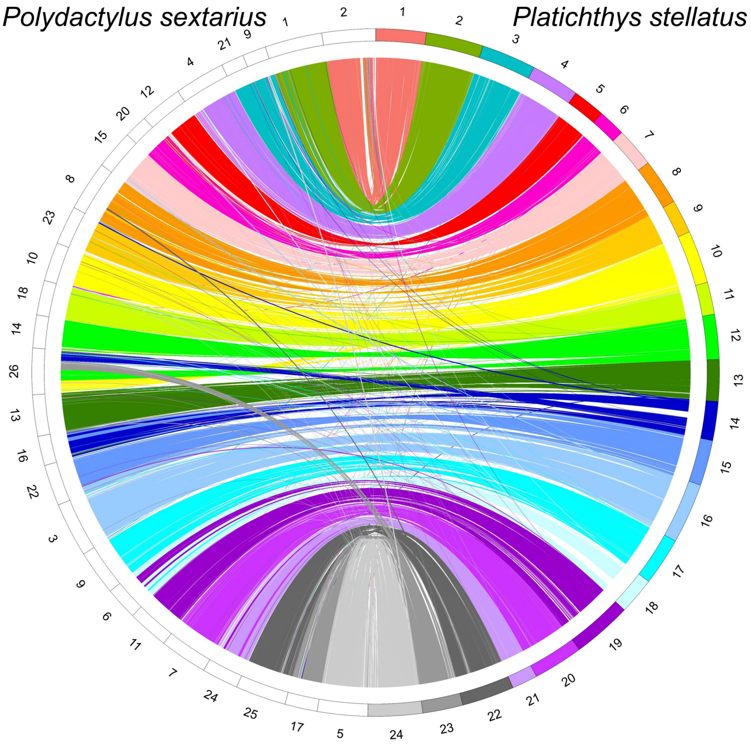

Supplementary Fig. 9. Synteny between the *Platichthys stellatus* and *Polydactylus sextarius* genomes.

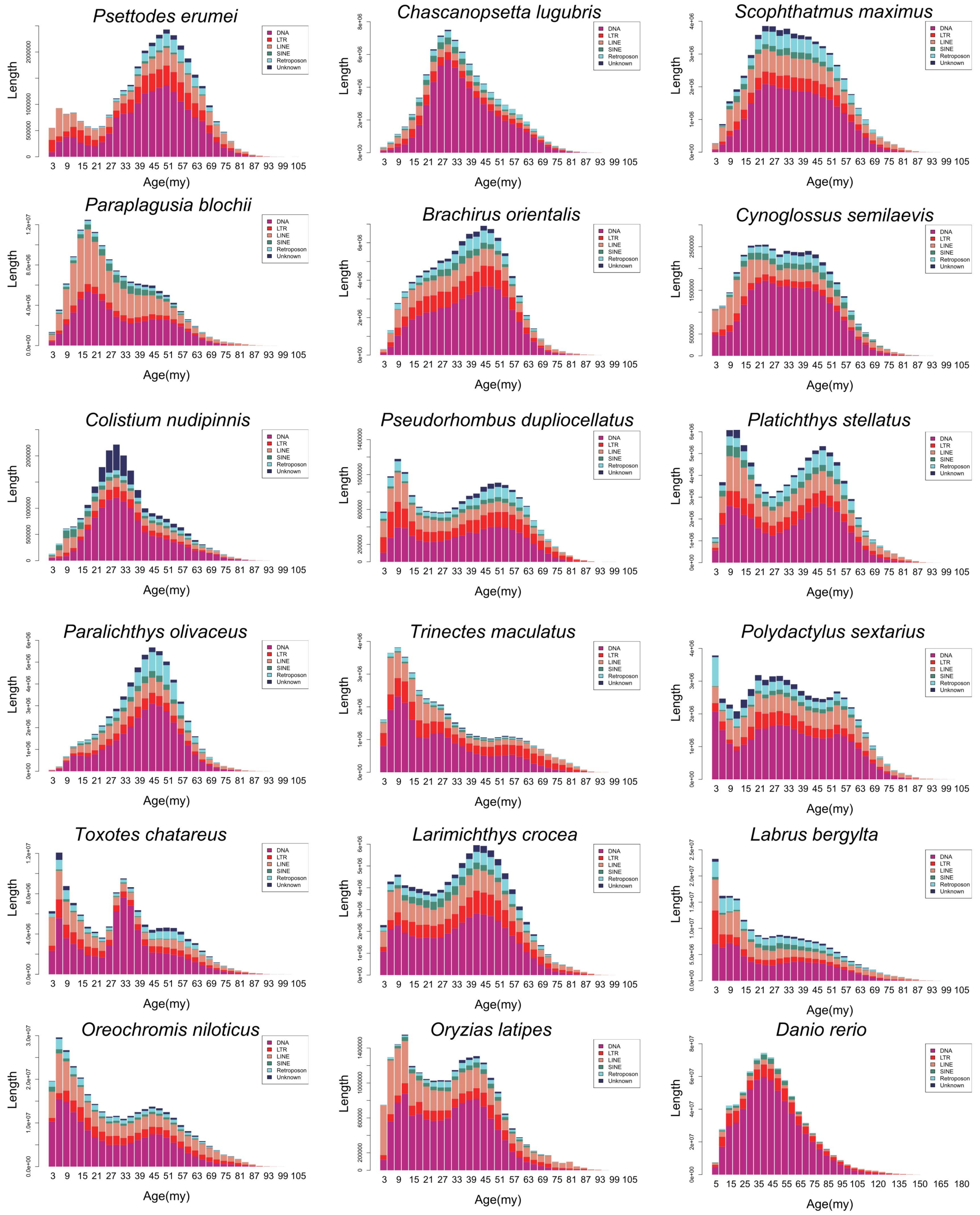

Supplementary Fig. 10. TEs explosion in species. The x-axis in each diagram represents the time (expressed as million years ago, my) when historical insertions of TEs into the genomes took place. The y-axis in each diagram represents the length of the insertions at each given time.

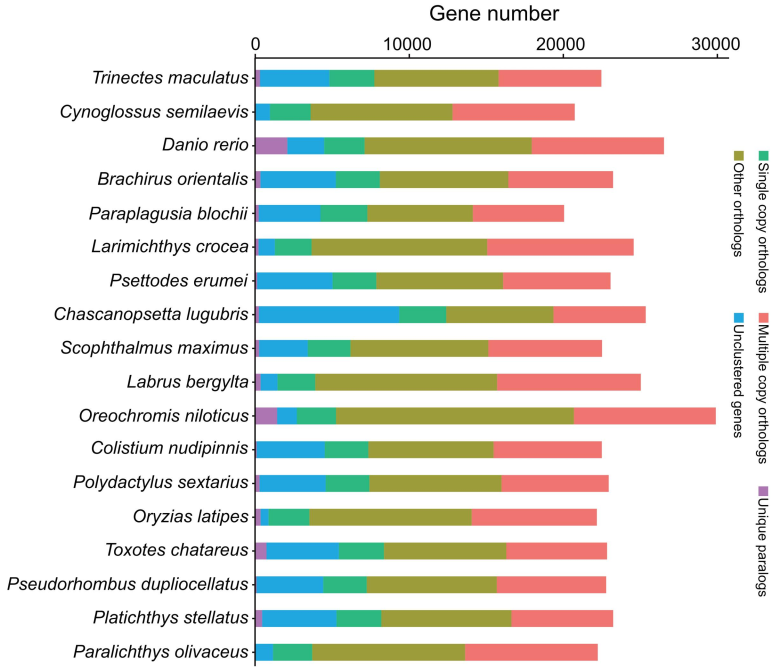

Supplementary Fig. 11. Statistics of the ortholog/paralog numbers among species. The y-axis in the diagram shows the name of the species, and the x-axis shows the numbers of ortholog/paralog identified in each species.

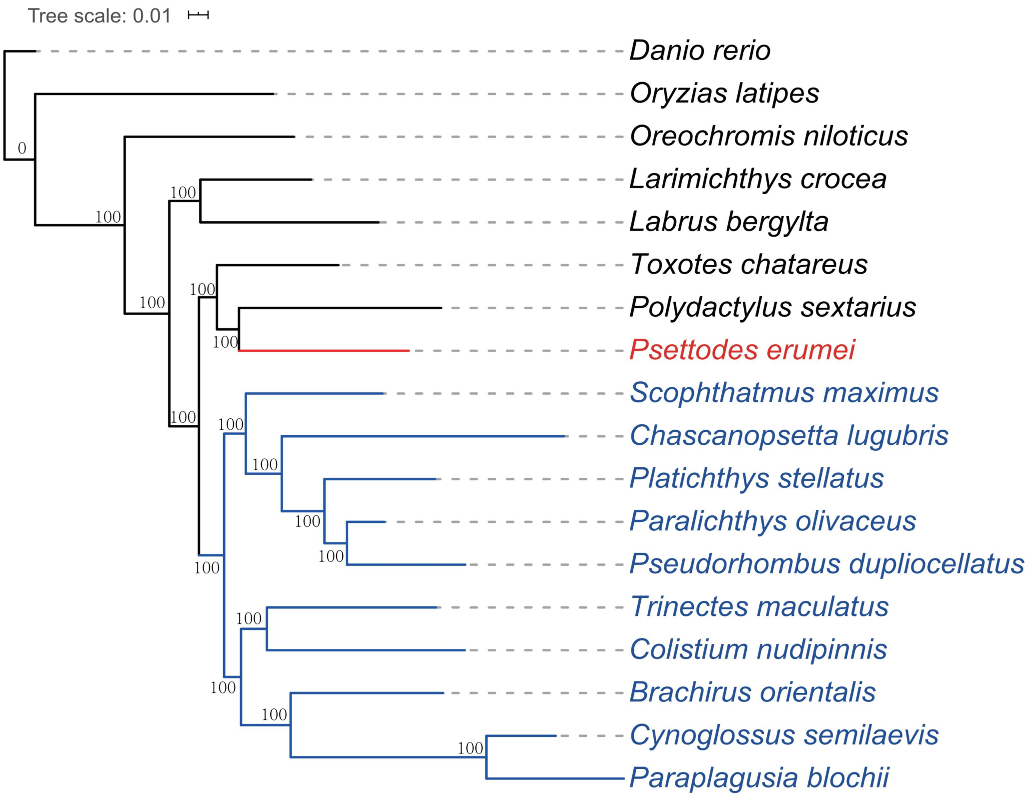

Supplementary Fig. 12. Phylogenetic relationship among flatfishes and other teleost

species analyzed using the concatenated single-copy genes (codon1+2+3).

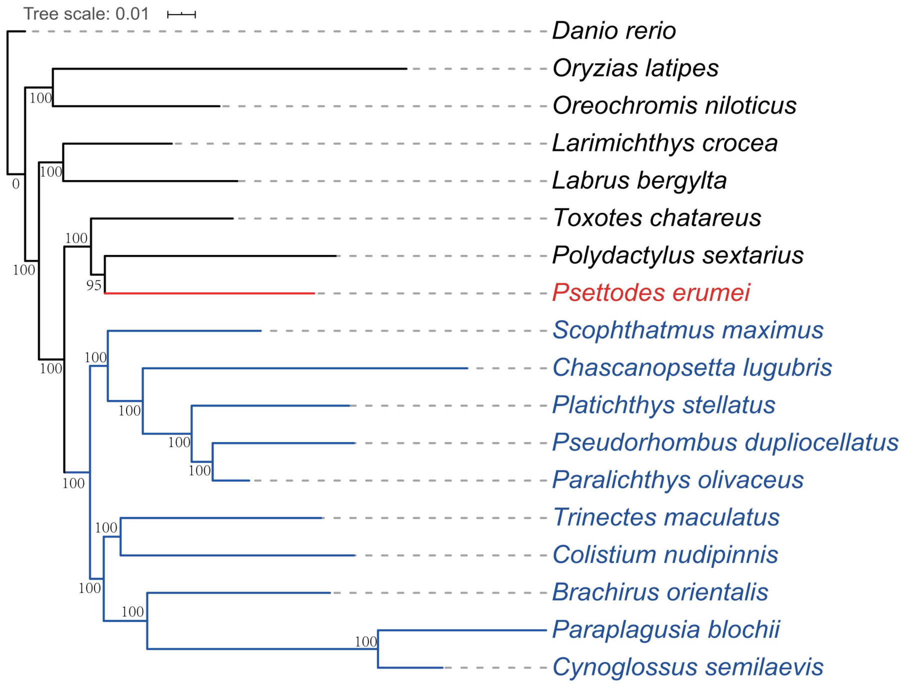

Supplementary Fig. 13. Phylogenetic relationship among flatfishes and other teleost species analyzed using the concatenated single-copy genes (codon1+2).

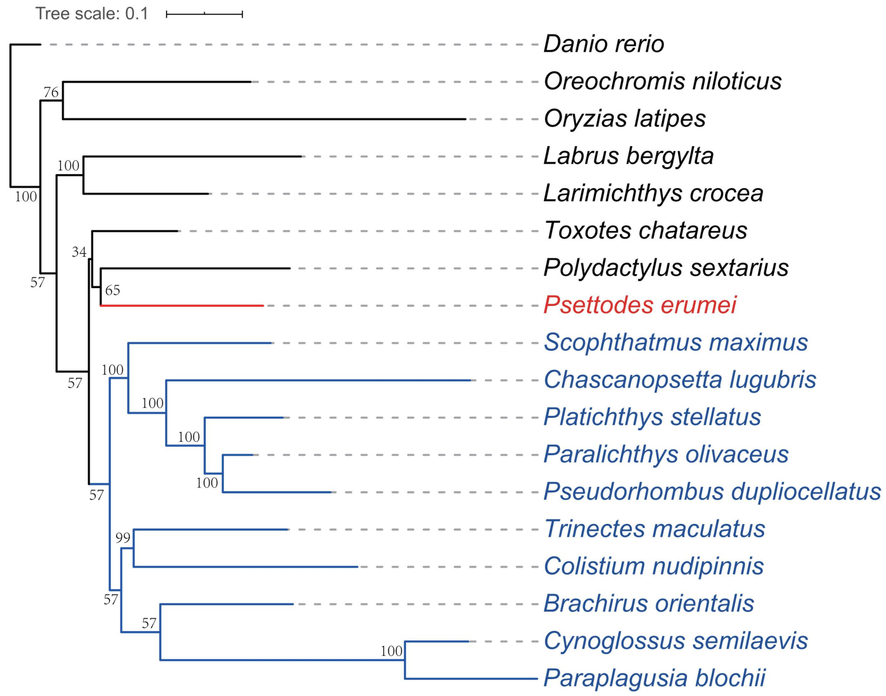

Supplementary Fig. 14. Phylogenetic relationship among flatfishes and other teleost species analyzed using the concatenated single-copy genes (4dTV).

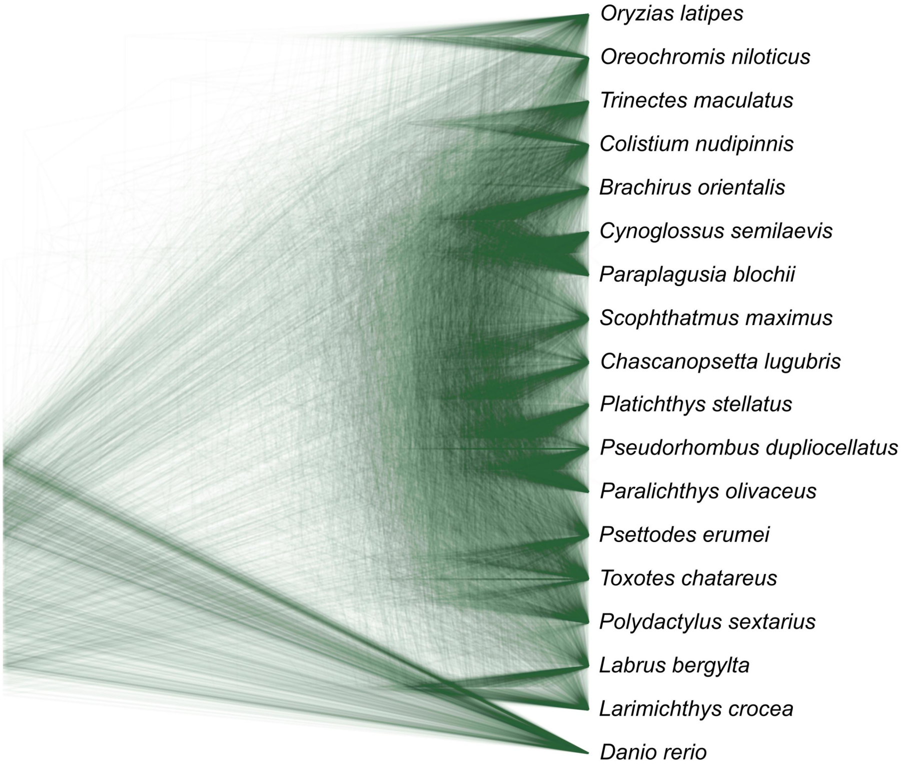

Supplementary Fig. 15. Simulation tree of flatfishes and other teleost species analyzed using the single-copy genes.

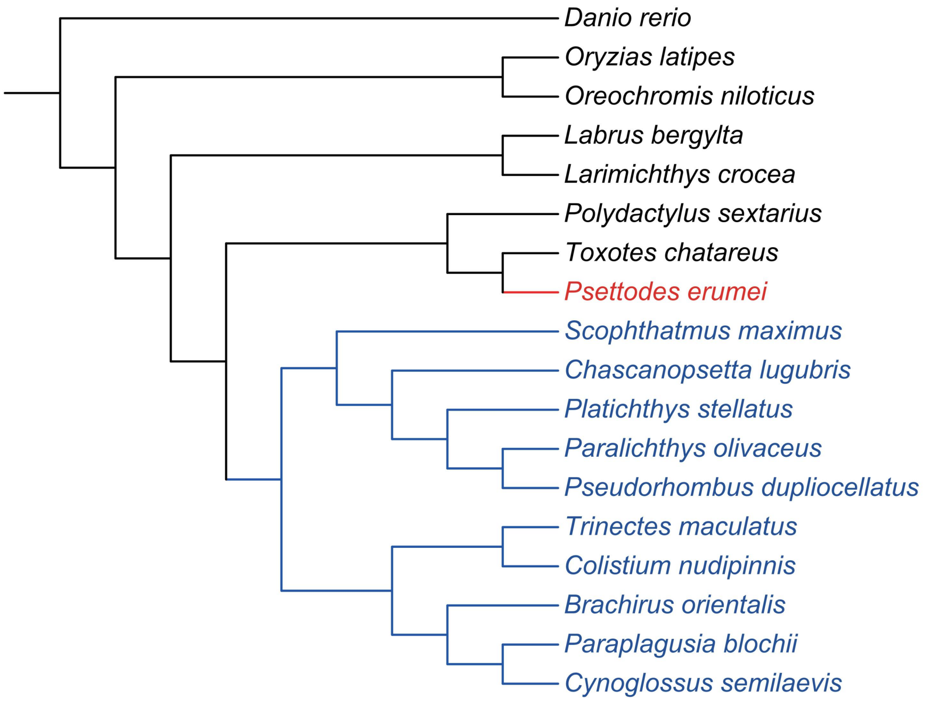

Supplementary Fig. 16. Species tree of flatfishes and other teleost species analyzed using

MP-EST.

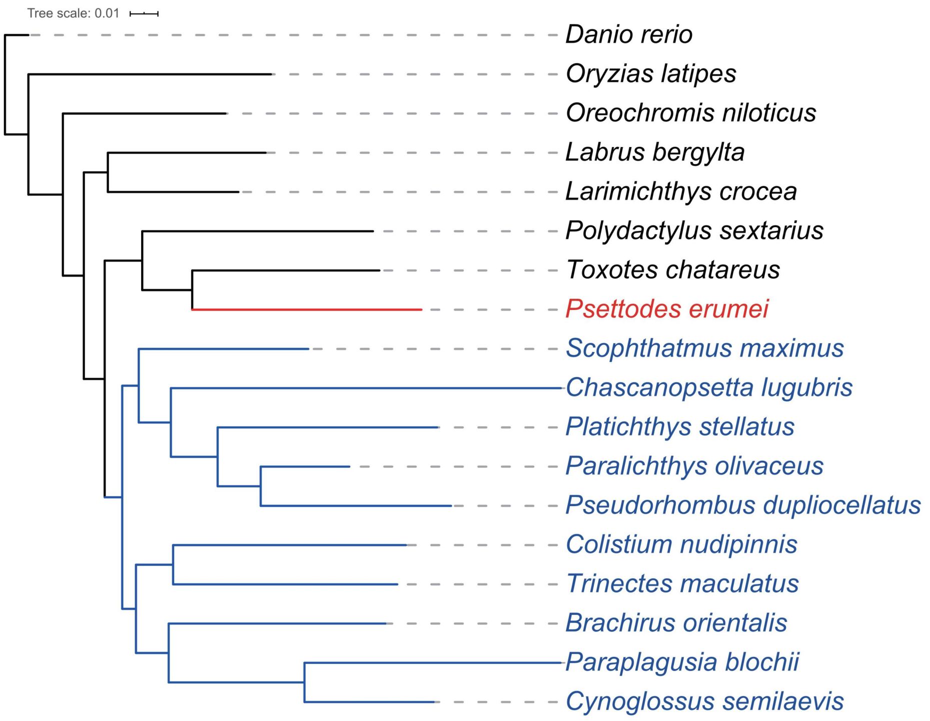

Supplementary Fig. 17. Species tree of flatfishes and other teleost species analyzed using OrthoFinder.

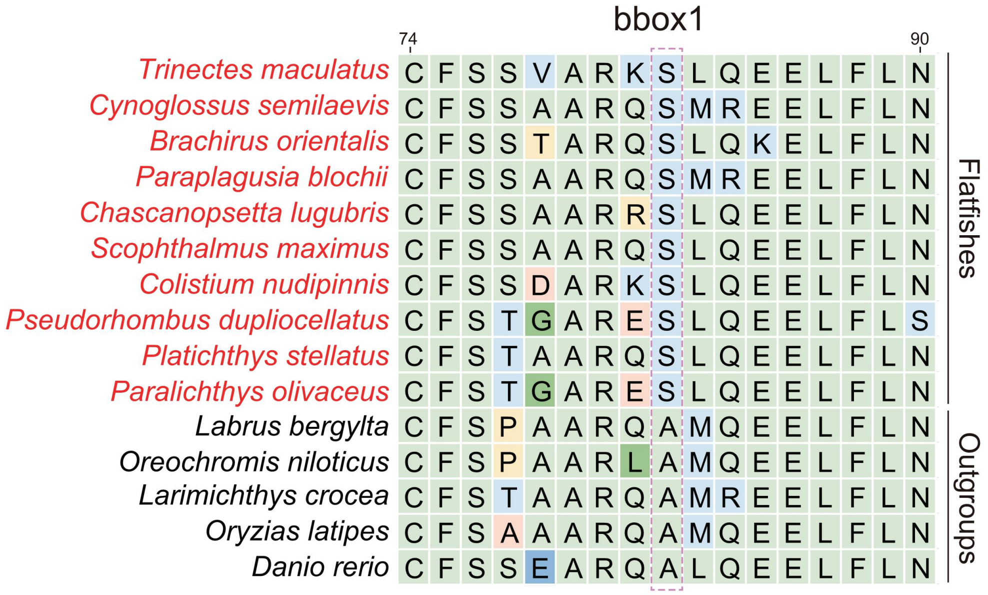

Supplementary Fig. 18. The rapidly evolving gene of *bbox1* in real flatfish Pleuronectoidei species. The sites that showed variation between species are marked in different colors. The fixed variation site between real flatfish Pleuronectoidei species and

outgroups are marked with a dashed box.

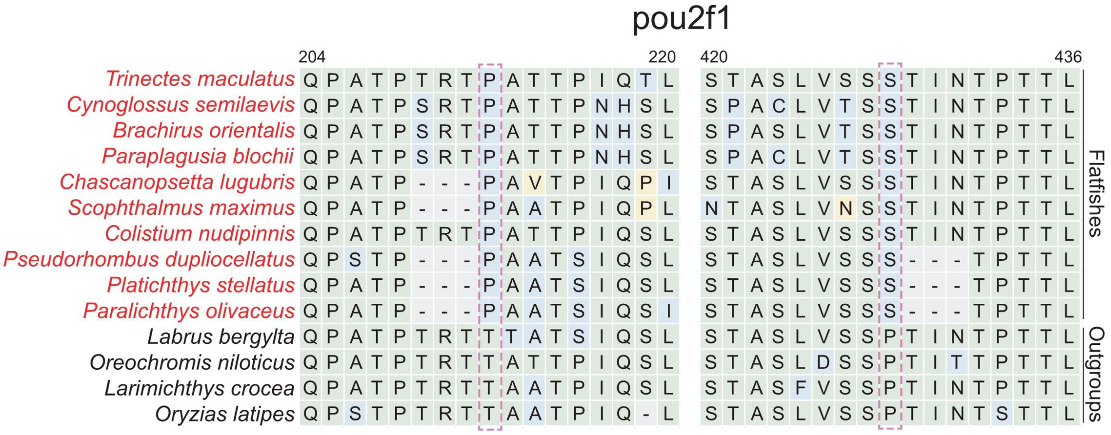

Supplementary Fig. 19. The rapidly evolving gene of *pou2f1* in real flatfish Pleuronectoidei species. The sites that showed variation between species are marked in different colors. The fixed variation site between real flatfish Pleuronectoidei species and outgroups are marked with a dashed box.

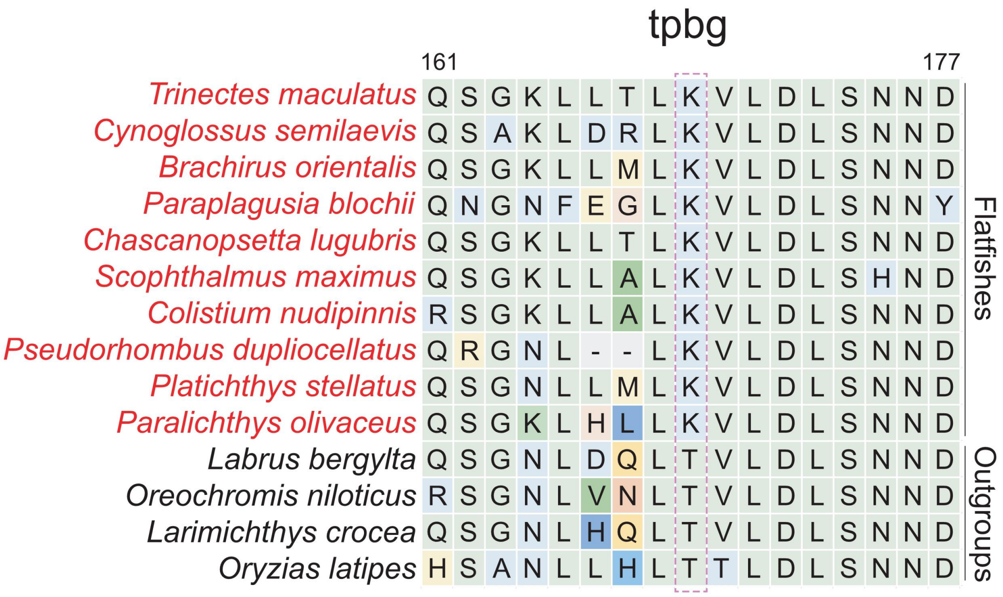

Supplementary Fig. 20. The positively selected gene of *tpbg* in real flatfish Pleuronectoidei species. The sites that showed variation between species are marked in different colors. The fixed variation site between real flatfish Pleuronectoidei species and outgroups are marked with a dashed box.

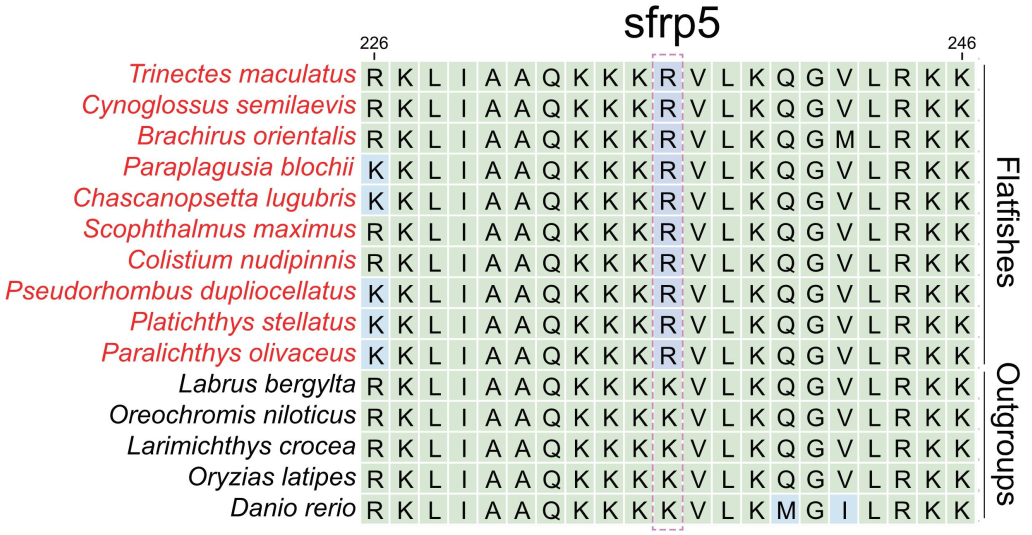

Supplementary Fig. 21. The lineage specific mutated gene of *sfrp5* in real flatfish Pleuronectoidei species. The sites that showed variation between species are marked in different colors. The fixed variation site between real flatfish Pleuronectoidei species and outgroups are marked with a dashed box.

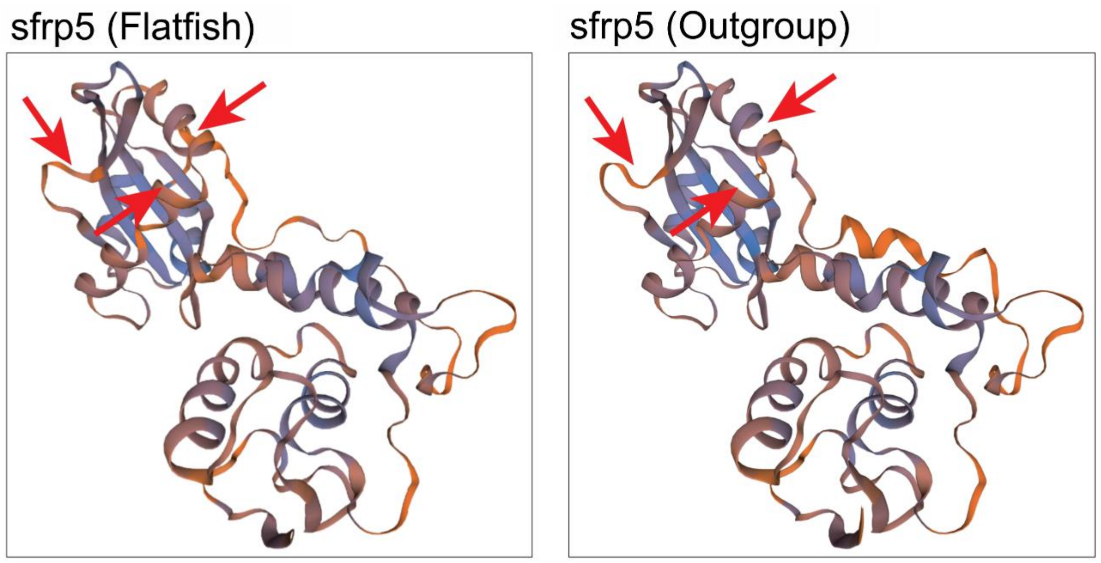

Supplementary Fig. 22. The 3D structure changes in protein of sfrp5 induced by the fixed mutation. The arrow showed the domains that having 3D structure changes.

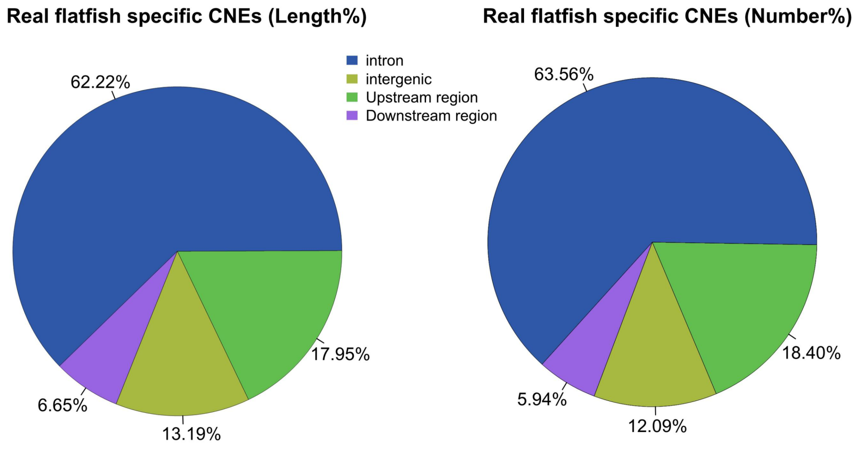

Supplementary Fig. 23. Statistics of SCNEs distribution in real flatfish Pleuronectoidei genomes. The results shown in the figure retain two significant digits after the decimal point.

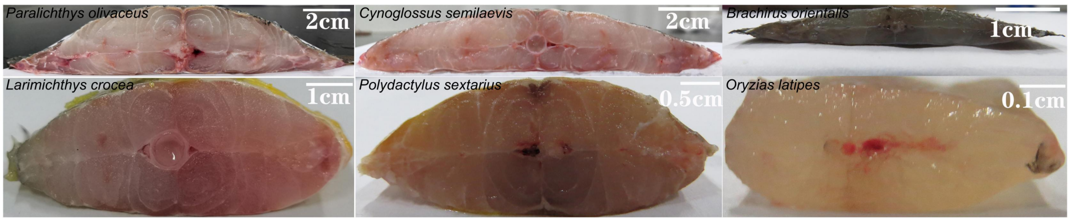

Supplementary Fig. 24. Body flatness of flatfishes relative to other non-flatfish teleost species. The images are enlarged to the same width for easy comparisons. The white line shows the scale of the fish.

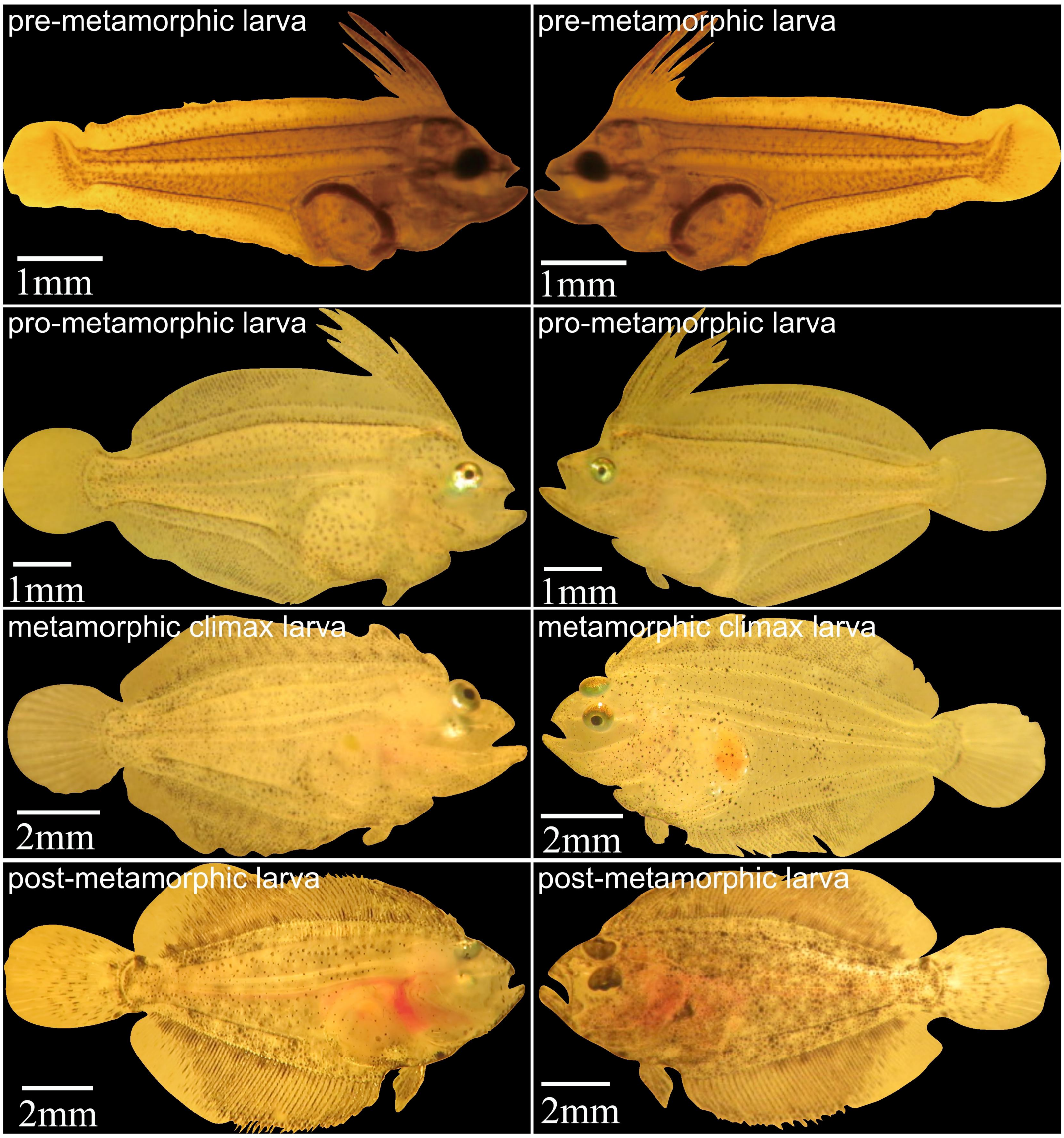

Supplementary Fig. 25. The metamorphosis process in flounder. Images from top to bottom shows the pre-metamorphic larva, pro-metamorphic larva, metamorphic climax larva, and post-metamorphic larva, respectively. The left and right panels respectively show the right and left sides of a same individual of each stage. The white line shows the scale of the fish.

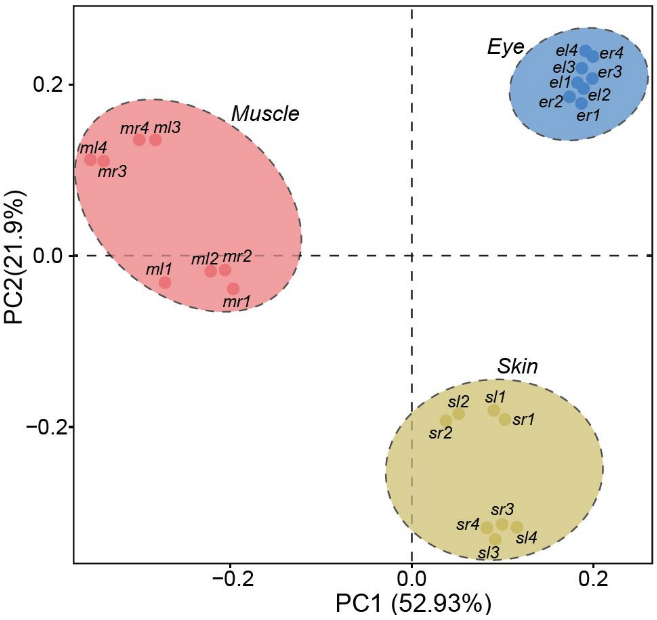

Supplementary Fig. 26. PCA analysis for RNA-seq samples in *Paralichthys olivaceus*. The letters m, e, and s represent the muscle, eye, and skin tissues, respectively. The number of 1, 2, 3, and 4 represent the four development stages (pre-metamorphic larva, pro-metamorphic larva, metamorphic climax larva, and post-metamorphic larva), respectively. The letters l and r represent the left and right-side of the body, respectively. Average gene expression levels were calculated from the three biological replicates for the PCA analysis.

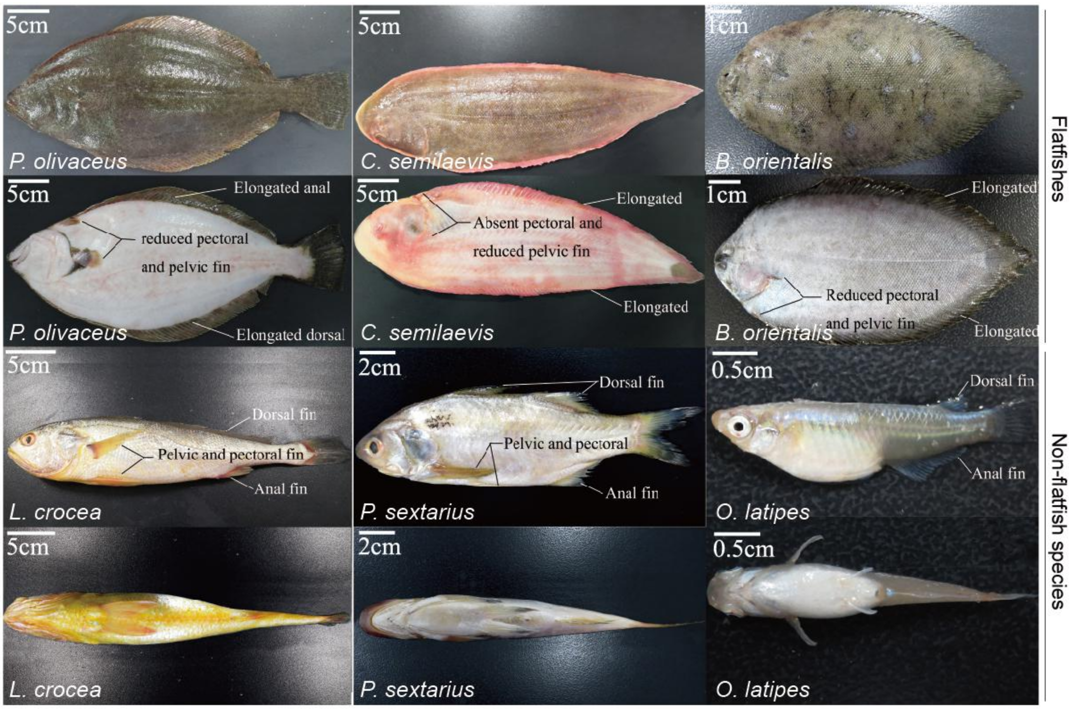

Supplementary Fig. 27. Images of dorsal, anal, pectoral, and pelvic fins in flatfishes

compared with non-flatfish species. For flatfishes, fins on both left and right sides are shown. For non-flatfishes, fins on both left and right sides are also shown, with additional view from the top of the abdomen. Three flatfish species, including *Paralichthys olivaceus*, *Cynoglossus semilaevis*, and *Brachirus orientalis*, and three outgroup species, including *Larimichthys crocea*, *Polydactylus sextarius*, and *Oryzias latipes*, were showed in this figure. The white line shows the scale of the fish.

Supplementary Tables

Supplementary Table 1. Sampling information for genome sequencing analysis in this study.

Supplementary Table 2. Sampling information for transcriptome analysis in *Platichthys stellatus*, *Toxotes chatareus*, *Polydactylus sextarius*, and *Paralichthys olivaceus*.

Supplementary Table 3. Statistics of the cleaned genomic sequencing data for *Trinectes maculatus*.

Supplementary Table 4. Statistics of the cleaned genomic sequencing data for *Chascanopsetta lugubris*.

Supplementary Table 5. Statistics of the cleaned genomic sequencing data for *Brachirus orientalis*.

Supplementary Table 6. Statistics of the cleaned genomic sequencing data for *Paraplagusia blochii*.

Supplementary Table 7. Statistics of the cleaned genomic sequencing data for *Colistium nudipinnis*.

Supplementary Table 8. Statistics of the cleaned genomic sequencing data for *Pseudorhombus dupliocellatus*.

Supplementary Table 9. Statistics of the cleaned genomic sequencing data for *Platichthys stellatus*.

Supplementary Table 10. Statistics of the cleaned genomic sequencing data for *Psettodes erumei*, *Toxotes chatareus*, and *Polydactylus sextarius*.

Supplementary Table 11. Statistics of the cleaned RNA-seq data for *Platichthys stellatus*, *Toxotes chatareus*, *Polydactylus sextarius*, and *Paralichthys olivaceus*.

Supplementary Table 12. Statistics of the cleaned genomic sequencing data of *Psettodes erumei*, *Toxotes chatareus*, and *Polydactylus sextarius* produced by the Nanopore platform.

Supplementary Table 13. Statistics of the cleaned Hi-C sequencing data for *Platichthys stellatus*, *Toxotes chatareus*, and *Polydactylus sextarius*.

Supplementary Table 14. Statistics of the *Trinectes maculatus* genome assembly.

Supplementary Table 15. Statistics of the *Brachirus orientalis* genome assembly.

Supplementary Table 16. Statistics of the *Paraplagusia blochii* genome assembly.

Supplementary Table 17. Statistics of the *Chascanopsetta lugubris* genome assembly.

Supplementary Table 18. Statistics of the *Colistium nudipinnis* genome assembly.

Supplementary Table 19. Statistics of the *Pseudorhombus dupliocellatus* genome assembly.

Supplementary Table 20. Statistics of the *Platichthys stellatus* genome assembly.

Supplementary Table 21. Statistics of the *Psettodes erumei* genome assembly.

Supplementary Table 22. Statistics of the *Polydactylus sextarius* genome assembly.

Supplementary Table 23. Statistics of the *Toxotes chatareus* genome assembly.

Supplementary Table 24. Statistics of the chromosome-level genome assembly for *Platichthys stellatus*.

Supplementary Table 25. Statistics of the chromosome-level genome assembly for *Toxotes chatareus*.

Supplementary Table 26. Statistics of the chromosome-level genome assembly for *Polydactylus sextarius*.

Supplementary Table 27. Statistics of the anchored chromosomes for *Platichthys stellatus* genome.

Supplementary Table 28. Statistics of the anchored chromosomes for *Toxotes chatareus* genome.

Supplementary Table 29. Statistics of the anchored chromosomes for *Polydactylus sextarius* genome.

Supplementary Table 30. Genome size estimated for each species in this study.

Supplementary Table 31. Genome assembly quality for *Trinectes maculatus* assessed by BUSCO analysis.

Supplementary Table 32. Genome assembly quality for *Chascanopsetta lugubris* assessed by BUSCO analysis.

Supplementary Table 33. Genome assembly quality for *Brachirus orientalis* assessed by BUSCO analysis.

Supplementary Table 34. Genome assembly quality for *Paraplagusia blochii* assessed by BUSCO analysis.

Supplementary Table 35. Genome assembly quality for *Colistium nudipinnis* assessed by BUSCO analysis.

Supplementary Table 36. Genome assembly quality for *Pseudorhombus dupliocellatus* assessed by BUSCO analysis.

Supplementary Table 37. Genome assembly quality for *Platichthys stellatus* evaluated assessed by BUSCO analysis.

Supplementary Table 38. Genome assembly quality for *Psettodes erumei* assessed by BUSCO analysis.

Supplementary Table 39. Genome assembly quality for *Toxotes chatareus* assessed by BUSCO analysis.

Supplementary Table 40. Genome assembly quality for *Polydactylus sextarius* assessed by BUSCO analysis.

Supplementary Table 41. Statistics of the Illumina reads mapped to the assembled genomes.

Supplementary Table 42. Results of transcript assembly for *Platichthys stellatus*.

Supplementary Table 43. Results of transcript assembly for *Toxotes chatareus*.

Supplementary Table 44. Results of transcript assembly for *Polydactylus sextarius*.

Supplementary Table 45. Statistics of the transcripts mapped to the *Platichthys stellatus* genome.

Supplementary Table 46. Statistics of the transcripts mapped to the *Toxotes chatareus* genome.

Supplementary Table 47. Statistics of the transcripts mapped to the *Polydactylus sextarius* genome.

Supplementary Table 48. Statistics of TEs in the *Trinectes maculatus* genome.

Supplementary Table 49. Statistics of repetitive sequences in the *Trinectes maculatus* genome.

Supplementary Table 50. Statistics of TEs in the *Brachirus orientalis* genome.

Supplementary Table 51. Statistics of repetitive sequences in the *Brachirus orientalis* genome.

Supplementary Table 52. Statistics of TEs in the *Paraplagusia blochii* genome.

Supplementary Table 53. Statistics of repetitive sequences in the *Paraplagusia blochii* genome.

Supplementary Table 54. Statistics of TEs in the *Platichthys stellatus* genome.

Supplementary Table 55. Statistics of repetitive sequences in the *Platichthys stellatus* genome.

Supplementary Table 56. Statistics of TEs in the *Chascanopsetta lugubris* genome.

Supplementary Table 57. Statistics of repetitive sequences in the *Chascanopsetta lugubris* genome.

Supplementary Table 58. Statistics of TEs in the *Colistium nudipinnis* genome.

Supplementary Table 59. Statistics of repetitive sequences in the *Colistium nudipinnis* genome.

Supplementary Table 60. Statistics of TEs in the *Pseudorhombus dupliocellatus* genome.

Supplementary Table 61. Statistics of repetitive sequences in the *Pseudorhombus dupliocellatus* genome.

Supplementary Table 62. Statistics of TEs in the *Psettodes erumei* genome.

Supplementary Table 63. Statistics of repetitive sequences in the *Psettodes erumei* genome.

Supplementary Table 64. Statistics of TEs in the *Toxotes chatareus* genome.

Supplementary Table 65. Statistics of repetitive sequences in the *Toxotes chatareus* genome.

Supplementary Table 66. Statistics of TEs in the *Polydactylus sextarius* genome.

Supplementary Table 67. Statistics of repetitive sequences in the *Polydactylus sextarius* genome.

Supplementary Table 68. Statistics of detailed repetitive categories in the analyzed species.

Supplementary Table 69. Statistics of protein-coding genes in species.

Supplementary Table 70. Functional annotation of protein-coding genes in *Trinectes maculatus*.

Supplementary Table 71. Functional annotation of protein-coding genes in *Brachirus orientalis*.

Supplementary Table 72. Functional annotation of protein-coding genes in *Paraplagusia blochii*.

Supplementary Table 73. Functional annotation of protein-coding genes in *Chascanopsetta lugubris*.

Supplementary Table 74. Functional annotation of protein-coding genes in *Colistium nudipinnis*.

Supplementary Table 75. Functional annotation of protein-coding genes in *Pseudorhombus dupliocellatus*.

Supplementary Table 76. Functional annotation of protein-coding genes in *Platichthys stellatus*.

Supplementary Table 77. Functional annotation of protein-coding genes in *Psettodes erumei*.

Supplementary Table 78. Functional annotation of protein-coding genes in *Toxotes chatareus*.

Supplementary Table 79. Functional annotation of protein-coding genes in *Polydactylus sextarius*.

Supplementary Table 80. The patterns of mutations in some annotated body plan genes in flatfishes and outgroups.

Supplementary Table 81. Relative evolutionary rates of species analyzed by LINTRE.

Supplementary Table 82. Relative evolutionary rates of species analyzed by Tajima test.

Supplementary Table 83. GO enrichment of expanded gene families in real flatfish Pleuronectoidei species.

Supplementary Table 84. GO enrichment of contracted gene families in real flatfish Pleuronectoidei species.

Supplementary Table 85. KEGG enrichment of contracted gene families in real flatfish Pleuronectoidei species.

Supplementary Table 86. GO enrichment of expanded gene families in flatfish-like Psettodoidei species.

Supplementary Table 87. KEGG enrichment of expanded gene families in flatfish-like Psettodoidei species.

Supplementary Table 88. GO enrichment of contracted gene families in flatfish-like Psettodoidei species.

Supplementary Table 89. KEGG enrichment of contracted gene families in flatfish-like Psettodoidei species.

Supplementary Table 90. Positively selected genes and rapidly evolving genes in real flatfish Pleuronectoidei species.

Supplementary Table 91. The positively selected genes and rapidly evolving genes in flatfish-like *Psettodes erumei*.

Supplementary Table 92. GO enrichment of positively selected genes and rapidly evolving genes in real flatfish Pleuronectoidei species.

Supplementary Table 93. KEGG enrichment of positively selected genes and rapidly evolving genes in real flatfish Pleuronectoidei species.

Supplementary Table 94. GO enrichment of positively selected genes and rapidly evolving genes in flatfish-like *Psettodes erumei*.

Supplementary Table 95. KEGG enrichment of positively selected genes and rapidly evolving genes in flatfish-like *Psettodes erumei*.

Supplementary Table 96. Lineage-specific mutations in real flatfish Pleuronectoidei species.

Supplementary Table 97. Genes with the same mutation in all flatfishes compared with outgroup species.

Supplementary Table 98. GO enrichment of SCNEs in real flatfish Pleuronectoidei species.

Supplementary Table 99. KEGG enrichment of SCNEs in real flatfish Pleuronectoidei species.

Supplementary Table 100. Tissue sampling in metamorphic flounders for RNA-seq.

Supplementary Table 101. KEGG enrichment of highly expressed genes in the muscle on the left side of pre-metamorphic flounders.

Supplementary Table 102. KEGG enrichment of highly expressed genes in the muscle on the left side of pro-metamorphic flounders.

Supplementary Table 103. KEGG enrichment of highly expressed genes in the muscle on

the left side of metamorphic climax flounders.

Supplementary Table 104. KEGG enrichment of highly expressed genes in the muscle on the left side of post-metamorphic flounders.

Supplementary Table 105. KEGG enrichment of highly expressed genes in the muscle on the right side of pre-metamorphic flounders.

Supplementary Table 106. KEGG enrichment of highly expressed genes in the muscle on the right side of pro-metamorphic flounders.

Supplementary Table 107. KEGG enrichment of highly expressed genes in the muscle on the right side of metamorphic climax flounders.

Supplementary Table 108. KEGG enrichment of highly expressed genes in the muscle on the right side of post-metamorphic flounders.

Supplementary Table 109. KEGG enrichment of highly expressed genes in the skin on the left side of pre-metamorphic flounders.

Supplementary Table 110. KEGG enrichment of highly expressed genes in the skin on the left side of pro-metamorphic flounders.

Supplementary Table 111. KEGG enrichment of highly expressed genes in the skin on the left side of metamorphic climax flounders.

Supplementary Table 112. KEGG enrichment of highly expressed genes in the skin on the left side of post-metamorphic flounders.

Supplementary Table 113. KEGG enrichment of highly expressed genes in the skin on the right side of pre-metamorphic flounders.

Supplementary Table 114. KEGG enrichment of highly expressed genes in the skin on the right side of pro-metamorphic flounders.

Supplementary Table 115. KEGG enrichment of highly expressed genes in the skin on the right side of metamorphic climax flounders.

Supplementary Table 116. KEGG enrichment of highly expressed genes in the skin on the right side of post-metamorphic flounders.

Supplementary Table 117. KEGG enrichment of highly expressed genes in the left eye of pre-metamorphic flounders.

Supplementary Table 118. KEGG enrichment of highly expressed genes in the left eye of

pro-metamorphic flounders.

Supplementary Table 119. KEGG enrichment of highly expressed genes in the left eye of metamorphic climax flounders.

Supplementary Table 120. KEGG enrichment of highly expressed genes in the left eye of post-metamorphic flounders.

Supplementary Table 121. KEGG enrichment of highly expressed genes in the right eye of pre-metamorphic flounders.

Supplementary Table 122. KEGG enrichment of highly expressed genes in the right eye of pro-metamorphic flounders.

Supplementary Table 123. KEGG enrichment of highly expressed genes in the right eye of metamorphic climax flounders.

Supplementary Table 124. KEGG enrichment of highly expressed genes in the right eye of post-metamorphic flounders.
