# Supplementary material for: Large-scale sequencing of flatfish genomes provides insights into the polyphyletic origin of their specialized body plan
Source: Nat Genet. 2021 Apr 19;53(5):742–51. doi: 10.1038/s41588-021-00836-9 (PMC8110480; doi:10.1038/s41588-021-00836-9)
Supplement: Supplementary file 2 — Reporting Summary [file 41588_2021_836_MOESM2_ESM.pdf]

nature research

Corresponding author(s): Yongxin Li; Xiaoyu Kong; Wen Wang

Last updated by author(s): Feb 1, 2021

Reporting Summary

Nature Research wishes to improve the reproducibility of the work that we publish. This form provides structure for consistency and transparency in reporting. For further information on Nature Research policies, see our Editorial Policies and the Editorial Policy Checklist.

Statistics

For all statistical analyses, confirm that the following items are present in the figure legend, table legend, main text, or Methods section.

n/a	Confirmed	
☐	☒	The exact sample size (*n*) for each experimental group/condition, given as a discrete number and unit of measurement
☐	☒	A statement on whether measurements were taken from distinct samples or whether the same sample was measured repeatedly
☐	☒	The statistical test(s) used AND whether they are one- or two-sided<br>*Only common tests should be described solely by name; describe more complex techniques in the Methods section.*
☒	☐	A description of all covariates tested
☐	☒	A description of any assumptions or corrections, such as tests of normality and adjustment for multiple comparisons
☐	☒	A full description of the statistical parameters including central tendency (e.g. means) or other basic estimates (e.g. regression coefficient) AND variation (e.g. standard deviation) or associated estimates of uncertainty (e.g. confidence intervals)
☐	☒	For null hypothesis testing, the test statistic (e.g. *F*, *t*, *r*) with confidence intervals, effect sizes, degrees of freedom and *P* value noted<br>*Give P values as exact values whenever suitable.*
☐	☒	For Bayesian analysis, information on the choice of priors and Markov chain Monte Carlo settings
☒	☐	For hierarchical and complex designs, identification of the appropriate level for tests and full reporting of outcomes
☒	☐	Estimates of effect sizes (e.g. Cohen's *d*, Pearson's *r*), indicating how they were calculated

Our web collection on statistics for biologists contains articles on many of the points above.

Software and code

Policy information about availability of computer code

Data collection: No software was used for data collection.

Data analysis: We list here the versions of all open-source tools used for data analysis.
1) HiC-Pro (v3.2);
2) Platanus (v1.2.4);
3) GapCloser (v1.10);
4) WTDBG (v1.2.8);
5) Pilon (v1.21);
6) Juicer (v1.5);
7) 3D de novo assembly (v170123);
8) Bridger (r2014-12-01);
9) BWA (v0.7.12);
10) BLAT (v34);
11) LAST (v802);
12) Tandem Repeat Finder (v4.04);
13) RepeatMasker (v4.0.6);
14) RepeatModeler (v1.0.8);
15) Augustus (v3.2.1);
16) GENSCAN (v1.0);
17) Genewise (v2.2.0);
18) PASA (v2.1.0);
19) EvidenceModeler (v1.1.1);
20) InterProScan (v4.8);

21) BLAST (v2.6.0);
22) OrthoMCL (v2.0.9);
23) MUSCLE (v3.8.31);
24) RAxML (v8.2.9);
25) MPEST (v2.0);
26) OrthoFinder (v2.3.5);
27) PAML (v4.8);
28) LINTRE (Unix version);
29) MEGA (v7.0.26);
30) CAFE (v3.1);
31) Tophat2 (v2.1.1);
32) Cufflinks (v2.2.1)

For manuscripts utilizing custom algorithms or software that are central to the research but not yet described in published literature, software must be made available to editors and reviewers. We strongly encourage code deposition in a community repository (e.g. GitHub). See the Nature Research guidelines for submitting code & software for further information.

Data

Policy information about availability of data

All manuscripts must include a data availability statement. This statement should provide the following information, where applicable:

- Accession codes, unique identifiers, or web links for publicly available datasets
- A list of figures that have associated raw data
- A description of any restrictions on data availability

All the sequencing data was deposited at the NCBI (Platichthys stellatus: PRJNA592732; Trinectes maculatus: PRJNA592733; Brachirus orientalis: PRJNA592734; Paraplagusia blochii: PRJNA592738; Chascanopsetta lugubris: PRJNA592739; Colistium nudipinnis: PRJNA592742; Pseudorhombus dupliocellatus: PRJNA592743; Polydactylus sextarius: PRJNA592744; Toxotes chatareus: PRJNA592745; Psettodes erumei: PRJNA592748; Paralichthys olivaceus: PRJNA632737). Besides, the source data of the fish photos were deposited at the Figshare database (https://doi.org/10.6084/m9.figshare.13664201.v1).

Field-specific reporting

Please select the one below that is the best fit for your research. If you are not sure, read the appropriate sections before making your selection.

☒ Life sciences ☐ Behavioural & social sciences ☐ Ecological, evolutionary & environmental sciences

For a reference copy of the document with all sections, see nature.com/documents/nr-reporting-summary-flat.pdf

Life sciences study design

All studies must disclose on these points even when the disclosure is negative.

Sample size

DNA were extracted from one sample for each species and sequenced at least 60X for the subsequent genome assembly. One sample for each species was used for genome sequencing, which is the routines for genome analyses in many taxon (Chen et al, 2014, doi: 10.1038/ng.2890; Shao et al, 2017, doi: 10.1038/ng.3732). For transcriptome sequencing and q-PCR analysis in Paralichthys olivaceus, we actually sampled muscle, eye and skin from both sides of at least 30 individual larvae and then respectively pooled each type of tissues together to conduct RNA extraction. We repeated three times from different batch of larvae for the sample collection as three biological replicates. Body and fin morphology, fat content of each species were measured in three individuals. For catalytic activity assay of enzyme, we conducted three reaction replicates to determine the mean +/- SD and calculate the statistical significance of differences between groups with a p value.

Data exclusions

No data were excluded.

Replication

For transcriptome sequencing and real-time PCR analysis, the analyses were conducted on three replicates for each tissue/stage (Paralichthys olivaceus). For body and fin morphology, fat content, the analyses were conducted on three individuals for each species. For catalytic activity assay of enzyme, we conducted three reaction replicates. All these experiments were technically repeated to ensure the reliability of the results.

Randomization

The samples for genomic DNA sequencing of each species were all randomly picked. The flounder larvae per development stage were also randomly picked to test the expression profiles of genes possibly involved in the asymmetrical development of body plans. The samples were also randomly selected to analyze the fat content, body and fin morphology of flatfishes, compared to outgroup teleosts.

Blinding

Investigators were blinded to group allocations during the specimen sampling, DNA/RNA extraction, library construction and sequencing process, and other phenotypical and biochemical analyses.

Reporting for specific materials, systems and methods

We require information from authors about some types of materials, experimental systems and methods used in many studies. Here, indicate whether each material, system or method listed is relevant to your study. If you are not sure if a list item applies to your research, read the appropriate section before selecting a response.

Materials & experimental systems

n/a	Involved in the study
☒	☐ Antibodies
☒	☐ Eukaryotic cell lines
☒	☐ Palaeontology and archaeology
☐	☒ Animals and other organisms
☒	☐ Human research participants
☒	☐ Clinical data
☒	☐ Dual use research of concern

Methods

n/a	Involved in the study
☒	☐ ChIP-seq
☒	☐ Flow cytometry
☒	☐ MRI-based neuroimaging

Animals and other organisms

Policy information about studies involving animals; ARRIVE guidelines recommended for reporting animal research

Laboratory animals

The flounder larvae used for the transcriptome analyses were collected from a flounder hatchery station located in Qingdao city, Shandong province, China.

Wild animals

Trinectes maculatus, Toxotes chatareus, and Colistium nudipinnis were collected from America, Thailand, and Australia, respectively, through laboratory to laboratory sample exchange programme. For other fish species, all the samples were collected by trawling from China during fishery resource surveys launched by South China Sea Institute of Oceanology, Chinese Academy of Sciences, and Zhejiang Ocean University. All the samples were collected dead from these survey activity and transported at low temperature from sampling sites to the laboratory and stored in -80 °C. For transcriptome analysis, adult fish and metamorphic flounder larvae were anaesthetized using MS-222 immersion before tissues were sampled. The adult fish and metamorphic larvae of Paralichthys olivaceus were used in the experiments and this specific study did not involve the sex of the species and so not identified, which has been clearly described in main text and supplementary information.

Field-collected samples

For genome sequencing, fat content, and phenotypical analysis, samples were collected dead from fishery resource surveys activities or through laboratory to laboratory sample exchange programme. For transcriptome analysis, adult fish and metamorphic flounder larvae were transported alive in aired seawater in low temperature and anaesthetized immediately after arrival using MS-222 immersion before tissues were sampled.

Ethics oversight

This study was approved by Institutional Animals Care and Use Committee of Zhejiang Ocean University and by Experimental Animal Management and Ethics Committee of South China Sea Institute of Oceanography, Chinese Academy of Sciences.

Note that full information on the approval of the study protocol must also be provided in the manuscript.
