# Supplementary material for: Large-scale sequencing of flatfish genomes provides insights into the polyphyletic origin of their specialized body plan
Source: Nat Genet. 2021 Apr 19;53(5):742–51. doi: 10.1038/s41588-021-00836-9 (PMC8110480; doi:10.1038/s41588-021-00836-9)
Supplement: Supplementary file 3 — Peer Review Information [file 41588_2021_836_MOESM3_ESM.pdf]

natureresearch

Peer Review Information

Journal: Nature Genetics
Manuscript Title: Large-scale sequencing of flatfish genomes provides insights into the polyphyletic origin of their specialized body plan
Corresponding author name(s): Dr. Yongxin Li

Reviewer Comments & Decisions:

Decision Letter, initial version:

Date: 20th Aug 20 17:58:57
Last Sent: 20th Aug 20 17:58:57
Triggered By: Tiago Faial
From: Tiago.Faial@us.nature.com
To: yxli28science@sina.com
Subject: Decision on Nature Genetics submission NG-A55146
Message: 20th Aug 2020

Dear Dr. Li,

Your Article, "Large-scale flatfish genome sequencing provides insights into non-monophyletic origin of their specialized body plan" has now been seen by 3 referees. You will see from their comments below that while they find your work of interest, some important points are raised. We are interested in the possibility of publishing your study in Nature Genetics, but would like to consider your response to these concerns in the form of a revised manuscript before we make a final decision on publication.

Reviewer #1 seems supportive overall and appears to have engaged deeply with this work. They raise a number of important issues regarding the evolutionary interpretation.
Reviewer #2, unfortunately did not submit a complete review, despite our multiple chase emails. They think that the analyses seem thorough and competently done, but that the conclusions (e.g. on the monophyly or rather lack thereof) about the

natureresearch

phylogeny of the Pleuronectiformes are not so new. Also, they note that some of the other conclusions, e.g. on the asymmetry, etc., have been suggested before based on single flatfish genomes.
Reviewer #3 is also positive regarding the experimental design and execution but brings up some potential issues with your interpretations. These points, along with reviewer #1's, need careful attention.

We therefore invite you to revise your manuscript taking into account all reviewer comments. Please highlight all changes in the manuscript text file. At this stage we will need you to upload a copy of the manuscript in MS Word .docx or similar editable format.

We are committed to providing a fair and constructive peer-review process. Do not hesitate to contact me if there are specific requests from the reviewers that you believe are technically impossible or unlikely to yield a meaningful outcome.

When revising your manuscript:

*1) Include a "Response to referees" document detailing, point-by-point, how you addressed each referee comment. If no action was taken to address a point, you must provide a compelling argument. This response will be sent back to the referees along with the revised manuscript.

*2) If you have not done so already please begin to revise your manuscript so that it conforms to our Article format instructions, available here. Refer also to any guidelines provided in this letter.

*3) Include a revised version of any required Reporting Summary:
https://www.nature.com/documents/nr-reporting-summary.pdf
It will be available to referees (and, potentially, statisticians) to aid in their evaluation if the manuscript goes back for peer review.
A revised checklist is essential for re-review of the paper.

Please be aware of our guidelines on digital image standards.

Please use the link below to submit your revised manuscript and related files:

[REDACTED]

Note: This URL links to your confidential home page and associated information about manuscripts you may have submitted, or that you are reviewing for us. If you wish to forward this email to co-authors, please delete the link to your homepage.

Please do not hesitate to contact me if you have any questions or would like to discuss these revisions further.

Nature Genetics is committed to improving transparency in authorship. As part of our efforts in this direction, we are now requesting that all authors identified as

‘corresponding author’ on published papers create and link their Open Researcher and Contributor Identifier (ORCID) with their account on the Manuscript Tracking System (MTS), prior to acceptance. ORCID helps the scientific community achieve unambiguous attribution of all scholarly contributions. You can create and link your ORCID from the home page of the MTS by clicking on ‘Modify my Springer Nature account’. For more information please visit please visit www.springernature.com/orcid.

We look forward to seeing the revised manuscript and thank you for the opportunity to review your work.

Sincerely,

Tiago

Tiago Faial, PhD
Senior Editor
Nature Genetics
https://orcid.org/0000-0003-0864-1200

Reviewers' Comments:

Reviewer #1:
Remarks to the Author:
The authors reported eight flatfish genomes including the Psettodes erumei which is regarded as one of the most primitive flatfish. Through comparative genomic analysis, they provided the well evidence that the origin of flatfish is non-monophyly. Based on this conclusion, they further provided the genetic basis of unique body plan of flatfish such as the flat body plan, asymmetric body plan and modified fins etc. The gene families or genes under positively selection or fast evolution that were identified and enriched in flatfish genomes has significant implications in understanding the unique flatfish body plan. In a word, this work is very nice and extends our understanding of the mechanisms of flatfish metamorphosis. The interpretation of the results is sound for the most part, and gives enough proof to verify their results.

My major concern is that these genes identified involving in the unique body plan are the cause of the metamorphosis or the adaptive result after the metamorphosis, which should be pointed out in the paper. Generally, in the evolutionary history of flatfish, the hampered musculature development may have started after the eyes moved to other side and the fish colonized on the bottom. Therefore, the hampered musculature development and the reduction of fat accumulation as well as other traits indicated in this paper are possibly the result of the asymmetry of the body, not the cause of the asymmetry. In addition, based on the previous research, the ancestors

of the flatfish should have lived in the relatively bottom waters, and their body plan already like the extant flatfish species. Therefore, based on the genes rapidly evolving from the genomes of extant flatfishes, if compared with the ancestor species, there should not be a great rapid evolution. If compared with fish with normal body plan, such as the zebrafish, medaka, the genes related to body shape should evolve very quickly. In this paper, how the comparison is made and how the out-group species are selected for different purpose, it is even more unclear.

Specific comments:
1.In line 116, it should provide the results of genome assembly for 8 flatfish species directly including the N50 size, the BUSCO etc. or at least the range in the main text, not just saying that the genome assembly have a high continuity an accuracy.
2.In line 123, the supplementary Figs. 5, 6 and 9 can not support the good genome synteny because the genome of zebrafish have evolved multi-chromosome rearrangement. So, I suggest to delete these figs.
3.In lines 124-133, the author said the genome size varied considerably among flatfishes from 399 Mb to 643 Mb, but in the supplementary table 30, it was showed the estimated genome sizes of flatfishes are not as different as the results of assembly in the main text. So, such difference may be result from the genome assembly for some species due to the different sequencing strategies. Correspondingly, the conclusion on the expand of LINE in the P. olivaceus compared to the P. dupliocellatus genome which has a biggest difference between estimated and assembly size is questionable. This may be caused by the LINE not being assembled in the P. dupliocellatus genome.
4.In line 137, the supplementary Fig. 16 just showed few flatfish species and zebrafish, I guess the author want to say here all the flatfish have the similar patterns of gene structure. So, it would be better to give all the flatfishes only in sup. Fig.16.
5.In line 142, it should be given full name when the species first appears and can be abbreviated when they appear again, please check it in the manuscript.
6.In line 148, please given the number of single-copy genes.
7.In lines 164-165, the authors have a conclusion on the non-monophyletic origin of flatfishes with settodoidei and Pleuronectoidei respectively arising from two independent evolutionary events. Could you give some analysis on the independent evolutionary events based on the flatfish genomes? Such as tracing the ancestor chromosomes of flatfish and to see if there is a difference during the chromosome evolution? This would be a proof for the non-monophyletic origin.
8.In line 188, please directly give the nucleotide substitution rates in RFPs, FLP and closely related perciformes species in the main text.
9.In lines 204-207, since those currently known genes to mediate body plan development are not involved in flatfish metamorphosis, it is thus recommended not to show this in the figure and you can put it in the supplementary file.
10.In lines 214-219, as I indicated that these genes associated with visual perception, immune response, hypoxia tolerance and cardiac function possible only reflect the adaptation after seafloor colonization but not the new body plan creation (line 237).
11.In line 248, it should be point out the detail information on the comparative genomic analysis here, which species, out-groups?
12.In lines 265-266, it is not making sense that the mutations in the flatfish genome are close to the known mutations in the human genome and thus are associated with the phenotype.

13.In lines 299-301, “the body plan asymmetry (such as asymmetrical eyes, cranium, and pigmentation) is another striking feature of flatfishes that confers advantages for living above substrates”. These characteristics are the inevitable result of the eye migration. Therefore, if you want to find genes that cause asymmetric body plan, you are actually looking for genes that cause eye migration. Currently, RA has literature support on eye migration, but WNT has no clear report, so it should be careful to deal with this pathway.
14.In lines 321-331, the authors said “three RA signal pathway genes have also undergone significant alteration in RFPs”; “Interestingly, our comparative genomic analyses revealed no obvious alteration of RA signal pathway genes in FLP”. If this is the case, it means the RA signal has not involved into the eye migration and thus not involved into metamorphosis.
15.In lines 331-333, why the different WNT signaling pathway genes plays in the body plan in RFP and FLP? It may also another proof on the non-monophyly.
16.In lines 336-343, it would be interesting to find that the RA, WNT genes exhibit a significant left-right asymmetrical expression. This result should be verified by qPCR at least.
17.Please follow the gene nomenclature of teleost species.

Reviewer #2:
Remarks to the Author:
The manuscript reports on a very large and comprehensive study in which 7 new long-read de novo genomes of flatfish (Order Pleuronectiformes) were sequenced as well as 3 other genomes of closely related perciform fishes that had been assumed to be sister lineages. Also 80 transcriptomes were created. One major issue that was addressed based on these data was the question of the monophyly of the flatfish order. This is an issue that had been addressed since Kyle 1923 and more recently with more and more molecular data sets. Various answers had been obtained. Bentancur et al. 2013 (Systematic Biology), which unfortunately was not cited here addresses this issue with sophisticated phylogenetic methods. Variously, the suborder Psettodoidei was placed more closely to other perciform fishes and not together in the Pleuronectiformes with the other suborder Pleuronectoidei.

In terms of the issue of asymmetry of left and right sides of flounders Li et al. provide more evidence for the role of Wnt and RA signalling. Previous work on flatfish genomics Shao et al. 2017 (Nature Genetics) had found that WNT and RA signaling does seem to play a role in the peculiar morphology of flat fishes.

Reviewer #3:
Remarks to the Author:
Lu Large-scale flatfish genome sequencing

Lu and colleagues sequenced the genomes of eight species of flatfish to learn the mechanisms behind their bizarre body plan. Few vertebrate clades have been sequenced in such breadth and depth. They performed extensive comparisons of genomes and identified fixed variations in a number of genes related to body asymmetry, muscle structure, and lipid metabolism. They performed RNA-seq of left

and right sides for several stages of metamorphosis. The sequencing all appears to have been accomplished in a competent fashion and the analysis is extensive.

General comments
I have made extensive suggestions on the language, which I hope will help the authors. These writing suggestions are in ALL CAPITAL LETTERS.
First, I think that the general method is precisely what needs to be done to spur research into this fascinating problem of the evolutionary developmental genetics of the flat body plan came about. The authors deserve applause for the efforts in assembling the biological material to perform the sequencing and performing such a large amount of sequencing and obtaining nearly chromosome-length genome sequences in some cases.
Second, it is good that they resolve the independent evolution of two different flatfish clades. This makes the evolutionary developmental genetics even more interesting.
A problem however exists with the authors' explanation for the independent evolution of flatness twice. While the independent evolution of the tens or hundreds of genes necessary to make a fish flat could have happened twice, the other possibility is that the last common ancestor of both groups evolved a flat body plan – the hundreds of genes evolved once – and that some. Main factors that cause flat body plans were secondarily lost twice, once in T. chatareus and once in P. sextarius. My bias is that it is easier to lose a highly specialized feature like flatness than to gain it and so it seems to me that it might be more likely to independently loose a specialized feature twice than to gain it twice. I think it is up to the authors to convince me that I'm wrong.
Another problem is that once the authors have identified flatfish-specific amino acid changes, they move to assuming mechanisms without showing that the mutations, the specific amino acid substitutions, actually would affect protein function. They just assume that these changes are causative. It is not at all clear that the gene pathways that they show and SAY explain the evolution of flatness actually DO link to flatness. I would expect that the causative changes would lie in gene expression changes rather than in changes in protein structure. That is my bias and I might be wrong. But it is the authors' duty to dissuade me of that bias. With respect to the gene expression studies, which were extensive – 80 libraries is a big experiment – and were done at appropriate stages, a problem was that DESeq2 on samples from four different individuals would have been better statistically than cufflinks on three 'biological replicates', and we are not told whether or not these replicates come from different individuals. The authors show for two genes some conserved non-coding elements that are clade specific but do not show that they actually would affect the expression of those genes, and if they did, whether expression changes in one or the other direction would lead to flatness.
All that being said, the authors provide a fantastic resource for performing experimental investigation of the problem of the evolution and development of the flat body plan that hopefully they and others will exploit to further our understanding of this remarkable trait.

Specific comments

34 been fully understood in the history of THE STUDY OF evolutionary biology since Darwin's time

36 clades (8/14) of Pleuronectiformes as well as two closely related species of

Perciformes[that presumably do not have flat body plans??].

40 ancestors about 76.1 and 80.0 million years ago RESPECTIVELY[??] via two independent evolutionary events.

42 of WNT and RA signaling in the evolution of body plan asymmetry.[PERIOD] THE FLAT BODY PLAN 43 may have evolved…

45 phenotype by adopting similar gene families and signal pathways
They did’t adopt gene families; they already had them. Adaptation may have occurred in similar gene families and signal pathways.

47 flatfish-like Psettodoidei provides a valuable model for studying the genetic origin of THE unique

58 have evolved the most specialized morphology and body plan THAT ever existed among teleosts
The body plan is extreme, but so is that of many others, exceptionally long eels, tiny cyprinids etc. In my opinion, this assertion should be toned down.

60 and thin body plan THAT facilitates

62 binocular vision, [comma] which ensures [lasting success of predation4,] and [a DELETE] modified median and
I don’t know if ‘lasting success of predstion’ means that they successfully avoid predation or this makes them more successful predators. Reword.

69 SOME progress HAS been made concerning the evolutionary origin and THE morphological

70 adaptations of the flatfishes in recent years. Current views support a THE origin of flatfishes AMONG BASALLY DIVERGING percoids

71 flatfishes, for their close resemblance in morphology, genetics, and evolution9-11. Despite THIS PROGRESS, there is still DISAGREEMENT regarding when and how FLATFISH diverged from

73 their ancestors. AN UNANSWERED QUESTION IS whether the flatfishes

74 and Psettodoidei, the only two suborders of this taxon)
What taxon? You’ve mentioned two, Pleuronectoidei and Psettodoidei, which one do you mean?

75 non-monophyletic origin due to controversies THAT resulted from

Such situations proved
77 defects in providing a solid evolutionary frame
I don’t know what this means. Should ‘proved’ be replaced by ‘included’?

79 predict differed genetic mechanismS for their

83 the first to elaborate this topic by applying A genomic framework

87 morphological adaptations (e.g. body plan flatness, body plan asymmetry and fin
What about eye migration?

93 of Perciformes
With more usual body plans???

94 added one more family (Scophthalmidae, Pleuronectoidei), and 80 transcriptomes
It would be more useful to say 'transcriptomes from an average of x organs from y species' or something to indicate breadth. 80 transcriptomes of 2 organs from one species would not be very helpful.

97 adaptation of flatfishes versus their ancestors; and 2) the genes THAT EXPERIENCED significant

103 Using [the DELETE] whole-genome sequencing strategies

109 species, spiny turbot (Psettodes erumei), spotted archerfish (Toxotes chatareus), and pacific
110 threadfin (Polydactylus sextarius),
Tell your readers which are flat fish and which not. Also, justify why these two specific non-flatfish. The tree makes it clear, but it should be in the text.

112 genomes of three species, starry flounder (Platichthys stellatus), spotted archerfish (T.
113 chatareus), and pacific threadfin (P. sextarius),
You don't need to give both common and Latin names twice.

119 scores (Supplementary Tables 31-40; see Supplementary Note 10), READ mapping ratios
120 (Supplementary Table 41; see Supplementary Note 10), and TRANSCRIPT mapping ratios

938 Geographical distribution of the species sequenced in this study. The DARK green area on

The legend should tell reader what the purple ovals are.

939 the map represents the global distribution regions of each species. The blue, red, and gray
940 circles
It would be so much easier to use L for left, R for right and B for both sides. The colors make your reader work too hard to learn a meaningless code.

940 circles next to the fish represents species that have left-SIDE eyes, right-SIDE eyes, or ONE EYE ON EACH SIDE of the body),

946 indicates the size of the DNA elements, LINE, SINE
The size of each element? Some LINES are longer than others. Or the fraction of the genome of each species that consists of LINEs etc?

Fig. 1e – it would help connectivities to have the same chromosome nomenclature across the entire clade.

130 considerable PROPORTION of

133 THAN the P. dupliocellatus genome

141 By combining our ten de novo assembled genomes with eight published GENOME SEQUENCES from

151 of suborder Psettodoidei formed one clade with Perciformes species WITHOUT FLAT BODIES, T. chatareus and

154 GENE TREES and SPECIES trees P. erumei is clustered with non-flatfish

155 lineage rather than with Pleuronectoidei species provided strong supports for independent
156 origins of Pleuronectoidei and Psettodoidei from lower-percoids
The other possibility is that the last common ancestor of both groups evolved a flat body plan that was secondarily lost in T. chatareus and P. sextarius. Text needs to present arguments to distinguish these two hypotheses.

166 first time, our results provided solid perspectives on the FLATFISH ORIGINS that has been

172 With [the DELETE] fossil calibration

173 approximately 76.1 and 80.0 million years ago (Ma), respectively IN THE late Cretaceous
I'm not qualified to critique this assertion. I hope someone with skills in that area is helping the authors get this correct.

174 Our time estimates were highly consistent with the time CALIBRATION by a previous

176 loci12,28. The late Mesozoic to early Cenozoic period, which includes THE Cretaceous

global peak pulse of fast seafloor spreading, which resulted in 182 a high stand of sea levels and
183 widespread epicontinental seas33.
Some of this repeats needlessly stuff just before.

185 and hence the eventual origin of both RFPs and FLP lineages.
Where in the world did these two lineages evolve? Were they sympatric?

192 Tables 80 and 81), which may explain why they EXHIBIT a "simply an asymmetric percoid

194 strong selection pressure they experienced34

How do you know that the rapid rate was not just due to very small population sizes and thus rapid drift?

197 Genes UNDERGOING significant alterations in flatfishes

201 FLP, Perciformes and other non-flatfish species. We analyzed [the DELETE] changed gene families in the
What does 'changed gene families' mean? Gene families whose individual proteins had rapid rates of sequence evolution? Or gene families that changed rapidly in gene number? Ambiguous.

208 identified genes UNDERGOING positive

209 lineage-specific mutation (LSGs) in both RFPs and FLP
This statement implies that you ruled out genes that experienced lineage specific mutation in only one of the two lineages. If you did that, then you would miss genes that changed in one flat fish lineage but not in the other and so could miss important causative agents.

non-coding elements (SCNEs) around the neighbouring regions 212 that potentially COULD RESULT in

214 The enrichment categories of top candidate genes under significant alteration in both RFP [NOT PLEURAL]

223 discovered for the first time TO BE ASSOCIATED WITH RAPID SEQUENCE EVOLUTION DURING AN evolutionary transition from THE water column to seafloor colonization.

225 this process may not only involve a cardiac morphological reorganization RESULTING from

227 involve cardiac functional remodeling RESULTING from selective

230 21). Such structural and functional alterations of THE cardiovascular system

231 may have contributed to their reinforced cardiac output, which is the highest [ever DELETE] known

233 readily encountered during in BURROWING INTO THE SUBSTRATE 54

The observed enrichment of genes associated with musculoskeletal 241 restructuring AND lipid

a phenotype
243 that is distinct in flatfishes (Fig. 4a; Supplementary Fig. 27; see Supplementary Note 23),
244 from which the name of "flatfishes" may arise.
Not needed.

952 red numbers represent the amounts of expanded and contracted gene FAMILIES

in this node,
Authors should add a scale bar to each image so reader can see how they vary in size as well.

953 respectively. The overall image of each species WAS drawn according

Fig. 3. Do the red and blue dots in 3A mean the position of the eyes? The same graphic device should mean the same thing throughout the paper.

Fig. 3a. What do the colored ovals mean?

Fig. 3b. This graphic isn't very helpful. It needs a device to show the size of the number so that the reader can get an impression of how gene family sizes change across the phylogeny.

Fig. 3c doesn't help me much. Consider cutting it even though it is cute.

958 the reference species. THE arrow represents

Fig. 4a. Error bars are too hard to see.

971 left-right axis. All the parameters were measured in three biological replicates for each
972 species.
Does that mean just three individuals for each species? Is that enough? How many species?
How does this parameter depend on the age of the animal? Or its standard length?

Fig 4b. The flatfish species need to be indicated by some graphic feature, like color in 3b.
Fig 4b. each aa or type of aa should have its own color so we can see easily how much variation exists at sites other than the one that the authors want to call our attention to.

245 advantage on the seafloor, where flatfishes usually [need to DELETE] hide from their enemies

250 (PSGs, P-value = 8.43e-3), SGCA (PSGs, P-value = 3.30e-3), SGCZ (SCNEs) and DMD
Tell reader what PSG and SCNE mean.

249 musculature development that have undergone significant alteration in RFPs, including THE SARCOLEMMA GENE SSPN
250 (PSGs, P-value = 8.43e-3), THE SARCOGLYCAN GENE SGCA (PSGs, P-value = 3.30e-3), SGCZ (SCNEs) and THE DYSTROPHIN GENE DMD

252 see Supplementary Notes 20 and 22). Unexpectedly, all FOUR OF these genes are

249 musculature development that have undergone significant alteration in RFPs
What does 'significant alteration' mean? Alteration in what parameter? Be specific.

256 development of muscular tissues58-60. Mutations or abnormal EXPRESSION of these four genes

260 that caused either structural or signaling modifications hampering normal musculature
261 development
The problem with this argument is that these all have to do with sarcomere structure, not the evolution of flatness. Flatness has to do with rib shapes and internal organ organization – you could have totally normal sarcomeres and be flat. The authors would have to show 1) that the sarcomeres of flatfish are different from 'normal ' fish in a way that's related to these proteins, and 2) that that difference is actually important for changing the shape of the fish. The point is that the thickness of the body wall, including the muscles, is not the primary factor in making the fish body flat. At least the authors have not shown that.

263 (sarcoglycanopathies) in humans throughout the world67
I would have thought that DMD was.

265 mutations were mapped very CLOSE to mutations associated with THE SYNDROMES of limb-girdle

268 CDC2 phosphorylation site (Fig. 4b) in SGCA, which has
Have such mutations been observed in human patients?

signal-dependent-activation profiles of muscular development 270 in RFPs that led to hampered
271 musculature and hence their flat phenotype.
Again, authors assume without proof that the musculature is the main reason that the fish are flat.

276 signals73,74 essential for ADIPOGENESIS

277 Mutations or 277 abnormal expressions of MEX3C and MLX would result in reduced adiposity
Zebrafish has an A at this position and flatfish have a G. These are conservative changes. What evidence is there to support the claim here that this specific mutation would result in reduced adiposity?
Fig. 4e shows that flatfishes differ from the other fish shown in crude fat, but that in no way means that this mutation in this gene is responsible.

982 were measured in three biological replicates for each species
What does biological replicate mean? Three different samples from one individual? If so, this is not meaningful. Also, sex is not given but sex and stage of reproductive cycle/season of the year affect this parameter. Were these the same in all species tested?

285 FOLD significantly lower.

286 muscular tissues respectively in RFPs than in [other DELETE] non-flatfish species

natureresearch

292 been adopted by both RFPs and FLP during the evolution of their flat body plan. TAKEN

293 together, our analyses provided the first piece of evidence supporting the role of hampered
294 development of musculature induced by altered DGC components, coupled with a restricted
295 lipid accumulation in evolution of the body flatness of flatfishes
These studies do show that flatfish have altered lipid biology but do not show how that phenotype is associated with flatness. Fig. 4e does not make that connection.

301 genetic basis remains largely unknown since Darwin's time8. RECENTLY, ADVANCES HAVE BEEN MADE UNDERSTANDING the genetic
302 regulation of body plan asymmetry in animals.

306 THE "NODAL-PITX2 signaling cassette

312 WNT9B (LSGs, L188M), SFRP5 (LSGs, K236R), TPBG (PSGs, P-value = 8.02e-4),
313 POU2F1 (
It is not possible to know the significance of these changes without additional data. 1) do they change the function of the proteins in some way relevant to body flattening? 2) lineage changes in many genes occur by chance in every large taxon, why do the authors focus on these? Are these the only lineage specific changes in the genome? For these genes, the authors don't go from unbiased look at all lineage specific function-changing mutations to see what they are involved in, but instead, take the biased approach of looking at their favorite genes and seeing if they have changes. 3) What does the P value mean? What is being compared to what?

329 the genetic variation in these genes may point to a role of RA signaling 329 in the left-right body
Only if authors demonstrate that these specific amino acid changes they observe in fact alter the protein functions in a way known to affect body symmetry.

332 PATHWAY genes that have undergone

337 Our transcriptomic data analyses lend further SUPPORT to the INVOLVEMENT of WNT

339 representative, we showed that multiple genes in both RA (ALDH1, ALDH8, RDH5, RDH7,
340 RDH8, RDH11, RDH12, RDH13) and WNT (WNT1, WNT4, and WNT10) signal pathways
341 exhibited a significant left-right asymmetrical expression in three examined flounder tissues
These observations are interesting but to reveal mechanisms, we need to know that 1) these genes are not asymmetrically expressed in closely related bilaterally symmetric species, and 2) that these are among the most significantly differentially expressed genes left vs. right, and 3) that the species with both eyes on the left have one way of asymmetry and the species with eyes on the right exhibit the opposite direction of asymmetrical expression.

349 metamorphosis. This was again supported by the evidence that the left deviation of expression
350 of pigmentation genes, such as TYR101, MITF101, and TYRP1101 usually occurs after the
351 asymmetrical expression of RA and WNT signals in the skin of metamorphosing flounder
Yes, this is a good observation supporting the authors' position.

352 larvae (Figs. 5c,d). Interestingly, significant left-right asymmetric expression of NODAL
353 signaling genes (including NODAL, LEFTY, and PITX2) was also observed in the tissues of
These genes are also asymmetrically expressed in 'symmetric' species like medaka and zebrafish.

357 asymmetrical expression of RA and WNT signals. Although obvious cross-TALK between

371 analysis, when we measured the dorsal, anal, pectoral and pelvic FIN length of flatfish species

386 indispensable for specification of the zone of polarizing activity (ZPA)102.
Yes, true, but 1) this is for paired fins, not the dorsal and anal fin that enact the fin-feet walking, and 2) the K to R substitution is a conservative change. Where is the evidence that this would cause a change in protein function? 3) is this hoxd12a or hoxd12b? 4) I don't see a K at position 105. 5) the outgroups the authors chose to present all have K at this position, but is this the only lineage among all teleosts or all vertebrates that has R at this position for both hoxd12a and hoxd12b?

>XP_019945365.1 PREDICTED: homeobox protein Hox-D12 [Paralichthys olivaceus]
1 memcernpln psyvgsllnf appdslyfsn lrgngahipg lhqlpynrre vctlpwtsss
61 sctsrgaqpa aaqsrafggy cppflsssvs lnssgghira hleepvrcfq dvghkaeeag
121 rreevyageh galsdggysd vhgrphgvaa htdaesagpl nvngtkqehd plqpparntc
181 srtsftegap wcssqvkirk krkpyskpql aelenefmmn efinrqkrke lsnrldlsdq
241 qvkiwfqnrr mkkkrlmmrd qafsay

392 of lysine105 to arginine105 in HOXD12,
This statement repeats info from above.
Suppl table 96 needs to give the accession number for each of these proteins, otherwise, how will reader be able to know what amino acid authors really mean, as illustrated by my problem with Hoxd12.
Throughout, the P as an abbreviation for the species is insufficient because it makes P. erumei and P. blochii and P. olivaceus all appear to be in the same genus. Ps. erumei and Pa. olivaceus, for example would help the non specialist.

411 genomics approaches to shed LIGHT on the evolutionary

420 Psettodoidei also exhibited unique mutation patterns in genes associated with less asymmetric body plan.
This seems to contradict that Psettodes erumei is asymmetric rather than symmetric. OK, I see, it's less asymmetric than flounder but more asymmetric than other

percoids. This sentence should be revised so not to confuse.

422 the phylogeny of flatfishes, while the genes highlighted in this study LAY a solid

443 maculatus, C. lugubris, B. orientalis, P. blochii, C. nudipinnis, P. dupliocellatus, and P.
As far as I could tell, the genera of many of these was given only in the 'data availability' section. The rule is that the first time a species is mentioned, it has to be the complete name.

456 species of P. stellatus, T. chatareus, P. sextarius, and P. olivaceus, the cDNA libraries were
The text does not say what organs or tissues were taken for study, even when these data are discussed.

512 Identification of orthologous genes. ORTHOLOGS were identified

522 best similarity pairs among species were considered as putative orthologs
This is good, but a comparison of conserved syntenies would be better, especially not to confuse the 'a' and 'b' copies from the teleost genome duplication.

524 Phylogenetic tree construction and divergence time evaluation. All the single-copy genes
Tell the reader how many genes that is.

531 OrthoFinder (v2.3.5)21. Divergence TIMES of these species were then estimated

542 much faster evolution rate using Chi-square test. All the single-copy genes were used in these
But the 'a' or the 'b' copy of duplicated genes might also be important in the evolution of flatfish traits. Excluding them from analysis will make the authors miss genes that might be important for evolution of traits.

562 IDENTIFICATION of genes

564 single copy genes among species were manually checked and
So did you exclude gene duplicates from the teleost genome duplication? Be clear.

574 Identification of conserved non-coding ELEMENTS. Using

575 the genomes of other species were aligned to the reference genome using
Which other species? Which genome was the reference genome?

587 The transcripts were assembled and gene expression values were analyzed using the cufflinks
Cufflinks is inadequate. The authors should have used DESeq2 because it gives a much better statistical treatment. I think it might be because DESeq2 works best with 4 or more replicates but they have just 3 'biological replicates', but they don't actually say if they come from 3 different individuals for each species.

It is very bothersome for the reviewer when the figures do not have figure numbers

on them.

971 left-right axis. All the parameters were measured in three biological replicates for each
972 species.
Does that mean three different individuals of each species? Be clear.

Fig. 4c – the exons and introns need to be marked and a scale ruler is needed across the gene.

Text does not make it clear what the take home message is for Fig 4c.

988 WNT9B gene in flatfishes compared with other non-flatfish teleosts
I saw no evidence that the M to L mutation would change the protein's function.

989 structure was shown on the top of the graph, and the site THAT SHOWED variation was marked

FIG 5b. Show where the exons are. Include a scale ruler in the horizontal axis.

Fig 5c is a nice series of images.

Fig 5e. What is the vertical dashed line?

Author Rebuttal to Initial comments

Reviewer #1:
The authors reported eight flatfish genomes including the *Psettodeserumei* which is regarded as one of the most primitive flatfish. Through comparative genomic analysis, they provided the well evidence that the origin of flatfish is non-monophyly. Based on this conclusion, they further provided the genetic basis of unique body plan of flatfish such as the flat body plan, asymmetric body plan and modified fins etc. The gene families or genes under positively selection or fast evolution that were identified and enriched in flatfish genomes has significant implications in understanding the unique flatfish body plan. In a word, this work is very nice and extends our understanding of the mechanisms of flatfish metamorphosis. The interpretation of the results is sound for the most part, and gives enough proof to verify their results.

Response: Thanks a lot for your positive comments on the manuscript.

My major concern is that these genes identified involving in the unique body plan are the cause of the metamorphosis or the adaptive result after the metamorphosis, which should be pointed out in the paper. Generally, in the evolutionary history of flatfish, the hampered musculature development may have started after the eyes moved to other side and the fish colonized on the bottom. Therefore, the

hampered musculature development and the reduction of fat accumulation as well as other traits indicated in this paper are possibly the result of the asymmetry of the body, not the cause of the asymmetry. In addition, based on the previous research, the ancestors of the flatfish should have lived in the relatively bottom waters, and their body plan already like the extant flatfish species. Therefore, based on the genes rapidly evolving from the genomes of extant flatfishes, if compared with the ancestor species, there should not be a great rapid evolution. If compared with fish with normal body plan, such as the zebrafish, medaka, the genes related to body shape should evolve very quickly. In this paper, how the comparison is made and how the outgroup species are selected for different purpose, it is even more unclear.

Response: Thank you for pointing out this important issue. Indeed body plan flatness and asymmetry are two different traits of flatfishes and it is possible that the hampered musculature development and the reduction of fat accumulation as well as other traits, are possibly the result of the asymmetry of the body, not the cause of the asymmetry, or had happened at the same time when the flatfishes were evolving asymmetric body plan. Accordingly, in the revision, we have clearly pointed out in related parts that the genes correlated with hampered musculature development, reduction of fat accumulation, and other traits are possibly the results of asymmetry and adaptation to the benthic life style of flatfishes after their metamorphosis and seafloor colonization. For example, we add this sentence in line 232, page 8 “The observed enrichment of genes associated with musculoskeletal restructuring and lipid deposition may reflect their roles in the evolution of body plan flatness after metamorphosis in flatfishes”. In addition, it is noteworthy that, in this study for the first time, we observe that genes related to musculature development and fat accumulation have undergone unique alternations, which may account for the hampered muscular development and low fat accumulation of flatfishes.

Regarding the outgroup species, we apologize for not clearly describing them when we introduced such results as positively selected genes, rapidly evolving genes, and so on. In the revision, rather than only describe them in the methods, we also clearly explain that we used *Larimichthys crocea*, *Labrus bergylta*, *Oreochromis niloticus*, *Oryzias latipes* and *Danio rerio* as the outgroup species, as well as the methods of the analysis. We have added the information of the outgroup species in line 237, page 8 of the main text and Method section in the revised manuscript.

Specific comments:

1.In line 116, it should provide the results of genome assembly for 8 flatfish species directly including the N50 size, the BUSCO etc. or at least the range in the main text, not just saying that the genome assembly have a high continuity an accuracy.

Response: Thank you for your suggestion. The ranges of N50 sizes, BUSCO scores, read mapping ratios, and transcript mapping ratios were added in lines 106-111, page 4 of the main text in the revised manuscript. The detailed information are also listed in the Supplementary files.

2.In line 123, the supplementary Figs. 5, 6 and 9 can not support the good genome synteny because the genome of zebrafish have evolved multi-chromosome rearrangement. So, I suggest to delete these figs.

Response: Thank you for this suggestion. We have deleted Supplementary Figs. 5, 6 and 9, and related statements.

3.In lines 124-133, the author said the genome size varied considerably among flatfishes from 399 Mb to 643 Mb, but in the supplementary table 30, it was showed the estimated genome sizes of flatfishes are not as different as the results of assembly in the main text. So, such difference may be result from the genome assembly for some species due to the different sequencing strategies. Correspondingly, the conclusion on the expand of LINE in the *P. olivaceus* compared to the *P. dupliocellatus* genome which has a biggest difference between estimated and assembly size is questionable. This may be caused by the LINE not being assembled in the *P. dupliocellatus* genome.

Response: We agree that the difference between estimated genome size and assembly in the main text may be a consequence of different sequencing and assembling strategies. Therefore, we rephrased this part and toned down the statement about genome size difference between flatfish species. Accordingly, we deleted the statement concerning the differentiation of the genome size between *Paralichthys olivaceus* and *Pseudorhombus dupliocellatus* being the consequence of LINE expansion in line 114, page 4 of the main text.

4.In line 137, the supplementary Fig. 16 just showed few flatfish species and zebrafish, I guess the author want to say here all the flatfish have the similar patterns of gene structure. So, it would be better to give all the flatfishes only in sup. Fig.16.

Response： Thank you for your suggestion. We tried to squeeze all species in a panel, however, it was too busy to clearly see so many different colored lines. So, we presented the results in two parts: the first part includes the top 4 panels showing 5 species sequenced in this study and 2 published species, and the next 4 panels show the other 5 sequenced species and 2 published ones (Supplementary Fig. 11).

5.In line 142, it should be given full name when the species first appears and can be abbreviated when they appear again, please check it in the manuscript.

Response: Yes, traditionally, it should be given full name when it first appears and should be abbreviated when it appears again. But one of the reviewers suggest that, to avoid the confusion caused by abbreviation because some of the species have same abbreviated genus name but belong to totally different genera. For instance, when we use *P* as an abbreviated name for genus of species, it is insufficient because it makes *Psettodes erumei*, *Paraplagusia blochii* and *Paralichthys olivaceus* all appear to be in the same genus. Therefore, we used the full name for each species throughout the manuscript.

6.In line 148, please given the number of single-copy genes.

Response: Thank you for your suggestion. We have added the number of single-copy genes and rephrased the sentence into “derived from 1,693 single-copy genes.....” in line 130, page 5 of the main text in the revised manuscript.

7.In lines 164-165, the authors have a conclusion on the non-monophyletic origin of flatfishes with Psettodoidei and Pleuronectoidei respectively arising from two independent evolutionary events. Could you give some analysis on the independent evolutionary events based on the flatfish genomes? Such as tracing the ancestor chromosomes of flatfish and to see if there is a difference during the chromosome evolution? This would be a proof for the non-monophyletic origin.

Response: Thank you for your valuable suggestion. Following your suggestion we constructed the ancestral chromosomes of all flatfishes using our chromosome level genomic data of *Platichthys stellatus* and *Cynoglossus semilaevis* (already published by Chen et al, 2014, *Nat. Genet.*) in real flatfish Pleuronectoidei (RFP) and *Toxotes chatareus,* and *Polydactylus sextarius* leading to the flatfish-like Psettodoidei (FLP) lineage (We did not obtain chromosome level assembly for *Psettodes erumei,* because of difficulties in sample collection). Indeed, no shared chromosome fusion or fission events were observed between the two lineages. We further used the contig sequences obtained from Nanopore reads of *Psettodes erumei* to check these lineage specific chromosome fusion and fission events, and found one contig in *Psettodes erumei* read through one of the lineage-specific chromosome fission identified in the Pleuronectoidei. As you suggested, these results further suggest possible independent origin of Psettodoidei and Pleuronectoidei. We briefly mentioned this conclusion in the lines 148-152 in the revised manuscript.

8. In line 188, please directly give the nucleotide substitution rates in RFPs, FLP and closely related perciformes species in the main text.

Response: We apologize for this quite misleading description. The nucleotide substitution rates here actually refered to “relative evolutionary rate”. We calculated relative evolutionary rate using *Platichthys stellatus* as the reference genome and compared the branch length of each species to the

reference genome in the obtained phylogenetic tree and got the value of confident probability, according to the method developed by Takezaki et al (1995, *Mol. Biol. Evol.*). Therefore, here there is only relative evolutionary rate compared with reference species and no absolute evolutionary rate value can be assigned to certain species. We have changed "nucleotide substitution rates" into "relative evolutionary rate" in this paragraph of the revised manuscript.

9. In lines 204-207, since those currently known genes to mediate body plan development are not involved in flatfish metamorphosis, it is thus recommended not to show this in the figure and you can put it in the supplementary file.

Response: Thank you for your suggestion. We have deleted this figure from the main text and put this figure in Supplementary Fig. 19 of the supplementary file.

10. In lines 214-219, as I indicated that these genes associated with visual perception, immune response, hypoxia tolerance and cardiac function possible only reflect the adaptation after seafloor colonization but not the new body plan creation (line 237).

Response: Yes, these genes may be benthic adaptation genes, and are not related to body axis generation. We described these genes here in order to exhibit a full scenario of what has been shaped in the flatfish genomes due to benthic adaptation, which is important for evolutionary origin of this bizarre taxa. And most interestingly, some of them such as cardiac morphogenesis genes are, for the first time, identified in flatfishes. We have pointed out clearly that all the visual perception, immune response, hypoxia tolerance and cardiac function may reflect the benthic adaptation after seafloor colonization. For example, we revise the phrase into "possibly suggesting a similar remodeling of their visual, immune, respiratory and circulatory systems in benthic adaptation to seafloor colonization" in lines 211-213, pages 7-8 of the main text to account for this point.

11. In line 248, it should be point out the detail information on the comparative genomic analysis here, which species, outgroups?

Response: Thank you for your suggestion. We used *Larimichthy scrocea*, *Labrus bergylta*, *Oreochromis niloticus*, *Oryzias latipes*, and *Danio rerio* as the outgroups. We have added the detailed information of which species we used as outgroups in our comparative genomic analysis in lines 237-238, page 8 of the main text in the revised manuscript by describing "Our comparative genomic analyses, using *Larimichthys crocea*, *Labrus bergylta*, *Oreochromis niloticus*, *Oryzias latipes*, and *Danio rerio* as the outgroups, revealed four genes.......".

12. In lines 265-266, it is not making sense that the mutations in the flatfish genome are close to the known mutations in the human genome and thus are associated with the phenotype.

Response: Thank you for pointing out this important issue. It is true that we can't assert that mutations in the flatfish genome are close to the known sites of mutation in the human genome and thereby are associated with the phenotype of flatfishes because of the too much divergence between these two taxa. However, we observed that the mutations are located within a conserved C-terminal intracellular domain (Fig. 3b), which contains the majority of functional sites of *SGCA* important for the signal-dependent-activated development of muscular tissues. Many site mutations in this domain have frequently been observed to cause hampered musculature development and severe muscular dystrophy such as limb-girdle muscular dystrophies (LGMD) syndrome in humans (Monies et al, 2016, *Hum. Genomics*; Xie et al, 2019, *Orphanet J. Rare Dis*.). Therefore, mutations in this domain may be very possibly correlated with the hampered musculature development and lean phenotype of flatfishes. To avoid misleading, we revised this sentence into "Both mutations locate within a conserved C-terminal intracellular domain (Fig. 3b), which is critical in the signal-dependent-activated development of muscular tissues....." in lines 250-254, page 9 of the main text in the revised manuscript.

13. In lines 299-301, "the body plan asymmetry (such as asymmetrical eyes, cranium, and pigmentation) is another striking feature of flatfishes that confers advantages for living above substrates". These characteristics are the inevitable result of the eye migration. Therefore, if you want to find genes that cause asymmetric body plan, you are actually looking for genes that cause eye migration. Currently, RA has literature support on eye migration, but WNT has no clear report, so it should be careful to deal with this pathway.

Response: Thank you for this important suggestion. Up to date, the role of *RA* signaling in the asymmetric development of the eyes and whole body plan has been demonstrated in the flounder *Paralichthys olivaceus*. In our study, using 11flatfish species, the marked selection signals on *RA* pathway genes further verified the role of *RA* signaling in the asymmetric development of eyes and body axis of flatfishes. At the same time, our comparative genomic analyses also detected strong selection signals in *WNT* pathway genes (Supplementary Table 90). *WNT* signaling genes also showed asymmetric expression during metamorphosis of flounder larva (Fig. 4d). These observations indicate *WNT* signaling pathway may also possibly be involved in the asymmetric development of body plan in flatfishes. Furthermore, intensive documents have indicated that *WNT* pathway played an important role in the left-right asymmetrical development of body plans in other animals, and there is even a complex "cross talk" between *WNT* and *RA* pathway in cell signaling (Harada et al, 2007, *Biochem. Biophys. Res. Commun*.; Yasuhara et al, 2010, *J Biol. Chem*.). But since no clear report regarding the effect of *WNT* on the eye migration was found in animals, as the reviewer pointed out, we toned down our description about the possible role of *WNT* in asymmetric development of flatfish body plan in the revised manuscript. For example, we add a sentence in our conclusion "……the exact role of these *RA* and *WNT* genes in the body plan asymmetry still awaits further investigations." in lines 354-355, page 12 of the main text to account for this point.

14.In lines 321-331, the authors said "three RA signal pathway genes have also undergone significant alteration in RFPs"; "Interestingly, our comparative genomic analyses revealed no obvious alteration of RA signal pathway genes in FLP". If this is the case, it means the RA signal has not involved into the eye migration and thus not involved into metamorphosis.

Response: Thank you for raising this important issue. Our comparative genomic analyses did not find any *RA* signaling pathway genes undergone marked alteration in *Psettodes erumei* genome. But whether the *RA* signaling pathway participates in the asymmetric body plan of *Psettodes erumei* or not is indeed worth further study, since *RA* has already been verified to play important role in eye migration and body plan asymmetry in the flatfish *Paralichthys olivaceus* using *in vivo* experiments (Shao et al, 2017, *Nat. Genet.*). Therefore, we used a more deliberative tone to interpret our finding concerning the role of *RA* in *Psettodes erumei* in the revised manuscript. For example, we have deleted the sentence ".....suggesting an exclusive contribution of *WNT* signaling to their flatfish-like asymmetrical phenotype in FLP" and rephrased into "It remains to be elucidated whether such distinction is related to the less extent of cranial asymmetry usually observed in FLP compared to typical RFP" in lines 321-322, page 11.

15.In lines 331-333, why the different WNT signaling pathway genes plays in the body plan in RFP and FLP? It may also another proof on the non-monophyly.

Response: Yes, it indeed provided another important evidence for non-monophyly of flatfishes. We added this discussion in the revision by describing "However, such distinction between RFP and FLP provides another evidence for the non-monophyletic origin of flatfishes" in lines 323-324, page 11.

16.In lines 336-343, it would be interesting to find that the RA, WNT genes exhibit a significant left-right asymmetrical expression. This result should be verified by qPCR at least.

Response: Thank you very much for this important suggestion. We have used qPCR to further verify the asymmetrical expression profile of these *RA* and *WNT* genes and the results have validated our previous results from transcriptome analysis. We have added the new results in lines 335-336 of the main text and in Supplementary Fig. 34 of the Supplementary file.

17.Please follow the gene nomenclature of teleost species

Response: Thank you for your suggestion. We have revised all the gene names following the gene nomenclature of teleost species, in the whole main text and supplementary file, as suggested. For example, we have rephrased the gene name of *HOXD12* into *HOXD12A* following the nomenclature of teleost species throughout the manuscript.

Reviewer #2:

The manuscript reports on a very large and comprehensive study in which 7 new long-read de novo genomes of flatfish (Order Pleuronectiformes) were sequenced as well as 3 other genomes of closely related perciform fishes that had been assumed to be sister lineages. Also 80 transcriptomes were created. One major issue that was addressed based on these data was the question of the monophyly of the flatfish order. This is an issue that had been addressed since Kyle 1923 and more recently with more and more molecular data sets. Various answers had been obtained. Bentancur et al. 2013 (Systematic Biology), which unfortunately was not cited here addresses this issue with sophisticated phylogenetic methods. Variously, the suborder Psettodoidei was placed more closely to other perciform fishes and not together in the Pleuronectiformes with the other suborder Pleuronectoidei.
In terms of the issue of asymmetry of left and right sides of flounders Li et al. provide more evidence for the role of *Wnt* and *RA*signalling. Previous work on flatfish genomics Shao et al. 2017 (Nature Genetics) had found that *WNT* and *RA* signaling does seem to play a role in the peculiar morphology of flat fishes

Response: Thank you very much for acknowledging the importance of our results.
Regarding the novelties of this study, it is indeed that Kyle (1923, *Phil. Trans. R.Soc. Lond. B.*) suspected the non-monophyly of the flatfishes mainly based on general fish morphology by such statement as “There are good reasons for believing, therefore, that the Flat-fishes are a composite group of Teleosts derived from several different sources”. However, it has been in fierce controversy about this issue since that time. Recently,
Betancur-R (2014, *Mol. Phylogenet. Evol.*), Harrington (2016, *BMC Evol.Biol.*) and Shi (2018, *BMC Genomics*), for instance, have provided evidence for monophyly of the flatfishes, whereas Dettai et al (2005, *Curr. Biol.*), Li (2011, *Mol. Phylogen. Evol.*), and Campbell (2013, *Mol. Phylogenet. Evol.*; 2014, *Mol. Phylogenet. Evol.*) inferred non-monophyly of the flatfishes. Therefore, up to now no clear conclusion has been reached regarding this issue. In this study, as you pointed out, we generated unprecedented large quantity of data and used them to get a well-resolved phylogeny based on multiple gene trees and species tree, which reveal a non-monophyletic origin of flatfishes, with real flatfish Pleuronectoidei and flatfish-like Psettodoidei, respectively, evolved from differed percoid ancestors through two independent evolutionary events. Based on this phylogenomic context, these results substantially clarified the long-standing controversies over the phylogeny concerning monophyly or non-monophyly of flatfishes.

In addition, through this analysis, we were also able to illuminate a number of important novel discoveries including: 1) we revealed for the first time that *WNT* signal pathways may also play a role in shaping the specialized body plan in flatfishes in addition to *RA* signaling. In Shao et al (2017,*Nat. Genet.*) study, they pioneeringly observed *RA* signaling pathway has shaped the asymmetric body plan in flatfishes, but no WNT; 2) for the first time we revealed that such genes as *SGCA*, *SSPN*, *BBOX1*, *MEX3C* and *MLX* may partially account for the flatness of the flatfishes; 3) our results show that although Pleuronectoidei and Psettodoidei evolved their flatfish morphologies through using similar

gene families and signal pathways, they also have lineage-specific mutations in body plan-related genes, which may partially explain why Psettodoidei resembles a Pleuronectoidei phenotype but still varies in degree of flatness and asymmetry in their body morphology. These discoveries have markedly extended understanding of the genetic basis of the specialized body plan of flatfishes, which would be difficult to be revealed using single species genome information. We apologized for overlooking the paper by Betancur-R et al (2013, *Syst. Biol.*), and we have cited this reference (reference 13 in this revision) to explain the fierce controversy about monophyletic or non-monophyletic origin of flatfishes in line 65, page 3 of the main text in the revised manuscript.

Reviewer #3:
Lu and colleagues sequenced the genomes of eight species of flatfish to learn the mechanisms behind their bizarre body plan. Few vertebrate clades have been sequenced in such breadth and depth. They performed extensive comparisons of genomes and identified fixed variations in a number of genes related to body asymmetry, muscle structure, and lipid metabolism. They performed RNA-seq of left and right sides for several stages of metamorphosis. The sequencing all appears to have been accomplished in a competent fashion and the analysis is extensive.
General comments
I have made extensive suggestions on the language, which I hope will help the authors. These writing suggestions are in ALL CAPITAL LETTERS.
First, I think that the general method is precisely what needs to be done to spur research into this fascinating problem of the evolutionary developmental genetics of the flat body plan came about. The authors deserve applause for the efforts in assembling the biological material to perform the sequencing and performing such a large amount of sequencing and obtaining nearly chromosome-length genome sequences in some cases.
Second, it is good that they resolve the independent evolution of two different flatfish clades. This makes the evolutionary developmental genetics even more interesting.

Response: Thank you very much for your positive comments and encouragement to our study, and we are especially grateful for your patient correction on the whole manuscript including the English grammar. In addition, due to tight space limit of a *Nature Genetics* article, we deleted some sentences or phrases. For example, we intensively compressed the abstract to about 100 words.

A problem however exists with the authors' explanation for the independent evolution of flatness twice. While the independent evolution of the tens or hundreds of genes necessary to make a fish flat could have happened twice, the other possibility is that the last common ancestor of both groups evolved a flat body plan – the hundreds of genes evolved once – and that some. Main factors that cause flat body plans were secondarily lost twice, once in T. chatareus and once in P. sextarius. My bias is that it is easier to lose a highly specialized feature like flatness than to gain it and so it seems to me that it might

be more likely to independently loose a specialized feature twice than to gain it twice. I think it is up to the authors to convince me that I'm wrong.

Response: Thank you for your intriguing proposal that the flatfishes might have originated from one evolutionary event but secondarily lost independently in lineage of *Toxotes chatareus* and *Polydactylus sextarius*. We have added the possibility of multiple losses after the discussion of two independent evolutionary events. Given the current data and evidence, the independent origin hypothesis seems more likely. Firstly, from the point view of evolutionary biology, multiple losses along a lineage are less parsimonious (Hillis and Moritz, 1990, *Molecular Systematics*, *Sinauer Associates In. Publication*). Considering that *Toxotes chatareus* and *Polydactylus sextarius* form independent sub-lineages in the Psettodoidei lineage rather than forming sister group to the *Psettodes erumei*, such multiple losses along the lineage is less likely according to parsimony principle of evolution. Here we used only *Toxotes chatareus* and *Polydactylus sextarius* as the outgroup of the Psettodoidei lineage, if more outgroup species such as *Sphyraena argentea*, *Centropomus armatus*, *Coryphaena hippurus*, *Nematistius pectoralis,* and many other perciforme species, which have also been included in Psettodoidei lineage as indicatedby previous phylogenetic studies (Li et al, 2009, *Mol. Phylogen. Evol.*; Betancur-R et al,2013, *PLoS Curr.*), were included, the loss events would be more and more. The chance of such large scale of multiple losses along a lineage is less likely. Secondly, intrigued by your proposal we looked at some body plan genes in the lineage of *Toxotes chatareus*, *Polydactylus sextarius* and *Psettodes erumei*, we observed majority of body plan genes in *Toxotes chatareus* and *Polydactylus sextarius* have the same mutations with perciformes instead of real flatfishes (Pleuronectoidei) (Supplementary Table 80). It is less likely that so many perciforme-like mutations in *Toxotes chatareus* and *Polydactylus sextarius* are consequences of reverse mutations according to the evolutionary principle that reverse mutations rarely happen (Li, 1997, *Molecular Evolution*). In this context, the multiple loss hypothesis seems less likely. We have added these new results and discussions about the two possible hypotheses in lines 138-148, page 5 of the main text.

Another problem is that once the authors have identified flatfish-specific amino acid changes, they move to assuming mechanisms without showing that the mutations, the specific amino acid substitutions, actually would affect protein function. They just assume that these changes are causative. It is not at all clear that the gene pathways that they show and SAY explain the evolution of flatness actually DO link to flatness.

Response: Thank you for pointing out this problem. We are sorry for the narrating manner by hurriedly moving to assuming mechanisms without showing that the mutations may affect protein functions right after describing flatfish-specific amino acid changes. To provide additional data to support our statements, in the revision, we not only conducted more experiments to provide functional evidence, but also rephrased our statements to avoid subjective conclusions.

Firstly, we conducted experiments to validate the expected functions of mutations in two enzyme-coding genes inferred from our comparative genomics analyses. The first is the *BBOX1* gene which encodes an enzyme to catalyze the formation of L-carnitine from gamma-butyrobetaine. RFP specific *BBOX1* genes and that of the outgroups were synthesized and cloned them into protein expression vectors, and then tested the catalytic dynamics for the proteins. The results show that RFP-specific BBOX1 has significantly higher (*P*-value < 0.01) activity catalyzing the formation of L-carnitine from gamma-butyrobetaine (Fig. 3d), indicating higher carnitine production in RFP. It is well-known that L-carnitine is a molecule critical in lipid oxidation (Zhao et al, 2020, *Gastroenterology*).Therefore, this result indicates that the RFP *BBOX1* may at least partially account for the low fat accumulation phenotype in RFP. The second protein we tested is the RDH14, which could mainly catalyze retinaldehyde back to retinol. Our result shows that the RFP RDH14 enzyme has 2.51 fold lower (*P*-value < 0.01) catalytic activity than that of outgroups (Supplementary Fig. 30), implying more retinaldehyde (substrate for RA synthesis) accumulation, thus possible *RA* signaling changes in the RFP. RA has been proved to be a critical factor in the induction of asymmetric body plan in flatfish (Shao et al, 2017, *Nat. Genet.*). The change in the RDH14 catalytic activity may thus play a role in the *RA* signaling-mediated body plan asymmetry.

Secondly, we used real-time quantitative PCR (qPCR) experiments to validate the asymmetric expression of several important genes, including *WNT10A* (core gene in *WNT* signaling), *PITX2* (core gene in *NODAL* signaling), *ALDH4A* and *ALDH9A* (core genes in *RA* signaling). The results confirmed their asymmetric expression (Supplementary Fig. 34) as suggested by the transcriptome analysis (Fig. 4d). All these three signaling pathways have been shown to play roles in formation of the left-right axis and regulation of the bilateral symmetry during the early development (Yoshioka et al, 1998, *Cell*; Vermot et al, 2005, *Science*; Vilhais-Neto et al, 2010, *Nature*; Kitajima et al, 2013, *Dev Biol.*; Watanabe et al, 2014, *Nature*; Minegishi, 2017, *Dev Cell.*). Moreover, abnormal expression of *RA* has also been proved to disrupt the development of the asymmetric body plan in Japanese flounder (Shao et al, 2017, *Nat. Genet.*). These results suggest the asymmetric expression of genes in these signaling pathways may have contributions in the asymmetric body plan in flatfishes, and are consistent to your comment that gene expression changes may has important roles in traits evolution. In addition, we also validated that two core genes (*MITF* and *TRYO*) in the melanin synthesis pathway were asymmetrically expressed during the metamorphosis process (Supplementary Fig. 34), which is correlated with the asymmetric pigmentation in the later stage of metamorphosis in flatfishes (Fig. 4c).

Finally, we carefully analyzed whether mutations in genes besides *BBOX1* and

RDH14 may have functional implications by looking at physicochemical property and 3D structure alternations. The results show that many amino acid substitutions in genes standing out from our comparative genomics analyses can alter either physicochemical property of amino acids or 3D

structure of proteins. For example, we found that the fixed amino acid substitution of *SFRP5* in RFP, which is a core inhibitor in *WNT* signaling pathway (Satoh et al, 2008, *Genesis*), can cause 3D structure change of this protein (Supplementary Fig. 24). Fixed amino acid substitution (T212P, P428S) of POU2F1, which is a positive activator in *WNT* signaling pathway (Katoh et al, 2005, *Int.J.Mol.Med.*) changed physicochemical property of amino acids. More such physicochemical property changing mutations are also observed for other genes, which have been described when we discussed those genes in the texts.

We have added these additional results of experiments and computational prediction in the revised manuscript. Furthermore, we also carefully toned down those sentences related to flatfish mutations throughout the manuscript to avoid any far-fetched conclusion.

I would expect that the causative changes would lie in gene expression changes rather than in changes in protein structure. That is my bias and I might be wrong. But it is the authors' duty to dissuade me of that bias. With respect to the gene expression studies, which were extensive – 80 libraries is a big experiment – and were done at appropriate stages, a problem was that DESeq2 on samples from four different individuals would have been better statistically than cufflinks on three 'biological replicates', and we are not told whether or not these replicates come from different individuals. The authors show for two genes some conserved non-coding elements that are clade specific but do not show that they actually would affect the expression of those genes, and if they did, whether expression changes in one or the other direction would lead to flatness.

Response: We agree that gene expression changes may play an important role in morphological trait evolution. We also managed to identify some asymmetrically expressed genes using left and right side transcriptomes of larvae at the metamorphosis stage. These genes are such key components as *WNT10A*, *PITX2*, *ALDH4A*, and *ALDH9A* in the *RA* and *WNT* signaling pathways, and the *RA* pathway has been proved to play a key role in the formation of asymmetric body plan (Shao et al, 2017, *Nat. Genet.*), supporting your idea about the roles of gene expression changes. Furthermore, during our revision process, as suggested the reviewer#1, we further used quantitative PCR (qPCR) experiments and validated their asymmetric expression patterns (Supplementary Fig. 34). However, at the current stage of phylogenomics, it is difficult to comprehensively study gene regulatory evolution in such non-model animals as flatfishes. We all are looking forward to the extension of such functional genomics projects as ENCODE into non-model organism, and at that moment we would have more power to understand the functional roles of gene expression changes in the flatfish evolution. In this study, we have to acknowledge that we are able to identify much more protein structure changes than gene regulation changes.

Regarding the biological replicates in the transcriptome analysis, we apologize for the ambiguous presentation. Because the metamorphic flounder larva is too small and tissue samples from one individual is far from enough for a regular transcriptome analysis, we actually dissected muscle, eyes and skin from both sides of at least 30 individual larvae and then respectively pooled each type of

tissues together to conduct RNA-seq. We repeated three times from different batch of larvae for the sample collection as three biological replicates. We have added how we collected samples in our transcriptome analysis in Supplementary Note 26 of the supplementary file. Yes, DESeq2 method for four samples would have good statistical significance. But Cufflinks method for from three samples (Secco et al, 2013, *Plant Cell*; Goldstein et al, 2017, *Genome Res*.) are also widely used in transcriptome analysis. Thank you for your suggestion.

Regarding the two genes that showed clade-specific conserved non-coding elements, we agree that it is difficult to show evidence that if they actually would affect the expression of those genes and whether expression changes in one or the other direction would lead to the specialized phenotype of flatfishes. Therefore, we have toned down the statement as "These RFP-specific mutations in the *RA* signaling genes might have played roles in the asymmetric body plan of RFP, though their actual role still awaits further verification." in lines 316-318, page 11 and "though the exact role of these *RA* and *WNT* genes in the body plan asymmetry still awaits further investigations" in lines 354-355, page 12 of the main text to account for this in the revised manuscript.

All that being said, the authors provide a fantastic resource for performing experimental investigation of the problem of the evolution and development of the flat body plan that hopefully they and others will exploit to further our understanding of this remarkable trait.

Response: Thank you very much again for your positive comments and encouragement to our work.

34 been fully understood in the history of THE STUDY OF evolutionary biology since
Darwin's time
36 clades (8/14) of Pleuronectiformes as well as two closely related species of Perciformes[that presumably do not have flat body plans??].
40 ancestors about 76.1 and 80.0 million years ago RESPECTIVELY[??] via two independent evolutionary events.
42 of WNT and RA signaling in the evolution of body plan asymmetry.[PERIOD] THE FLAT BODY PLAN 43 may have evolved.

Response: Thank you very much for your helps to improve these four phrases. But due to stringent space limit of *Nature Genetics*, we have to delete these less function-relevant sentences by following the guideline of *Nature Genetics*.

45 phenotype by adopting similar gene families and signal pathways
They did't adopt gene families; they already had them. Adaptation may have occurred in similar gene families and signal pathways.

Response: Sorry for this vague phrasing. We have revised this phrase into “Evolution of Psettodoidei involved similar but not identical genes.” in the revised manuscript.

47 flatfish-like Psettodoidei provides a valuable model for studying the genetic origin of THE unique

Response: We have added “the” here as suggested.

58 have evolved the most specialized morphology and body plan THAT ever existed among teleosts The body plan is extreme, but so is that of many others, exceptionally long eels, tiny cyprinids etc. In my opinion, this assertion should be toned down.

Response: Sorry for this exaggerated wording. We have changed this sentences into “have evolved a specialized morphology that is unique in teleosts”.

60 and thin body plan THAT facilitates

Response: Corrected as suggested.

62 binocular vision, [comma] which ensures [lasting success of predation4,] and [a DELETE] modified median and

I don’t know if ‘lasting success of predstion’means that they successfully avoid predation or this makes them more successful predators. Reword.

Response: We apologize for the previous ambiguous wording. We have revised this phrase into “ensures their improved success of predating”.

69 SOME progress HAS been made concerning the evolutionary origin and THE morphological

Response: Corrected as suggested.

70 adaptations of the flatfishes in recent years. Current views support a THE origin of flatfishes AMONG BASALLY DIVERGING percoids.

Response: Thank you for your suggestion. We have change the sentence into “Current views support the origin of flatfishes among basally diverged percoids.”

71 flatfishes, for their close resemblance in morphology, genetics, and evolution9-11. Despite THIS PROGRESS, there is still DISAGREEMENT regarding when and how FLATFISH diverged from

Response: Corrected as suggested.

73 their ancestors. AN UNANSWERED QUESTION IS whether the flatfishes

Response: Corrected as suggested.

74 and Psettodoidei, the only two suborders of this taxon)
What taxon? You've mentioned two, Pleuronectoidei and Psettodoidei, which one do you mean?
and Psettodoidei, the only two suborders of this taxon

Response: We apologize for this ambiguous wording. We have made it clear by rephrasing it into "Pleuronectoidei and Psettodoidei, the only two suborders of Pleuronectiformes".

75 non-monophyletic origin due to controversies THAT resulted from

Response: Due to stringent space limit of *Nature Genetics*, we deleted this sentence by following the guideline of *Nature Genetics*.

Such situations proved 77 defects in providing a solid evolutionary frame I don't know what this means. Should 'proved'be replaced by 'included'?

Response: Corrected as suggested. 79 predict differed

genetic mechanismS for their

Response: Corrected as suggested.

83 the first to elaborate this topic by applying A genomic framework

Response: Corrected as suggested.

87 morphological adaptations (e.g. body plan flatness, body plan asymmetry and fin What about eye migration?

Response: Thank you for pointing this. We have rephrased it into "while the genetic basis of a wider spectrum of morphological adaptations (e.g. body plan flatness, body and eye asymmetry, and fin modification) in the whole flatfish group remains to be explored from a systematic evolutionary perspective" here.

93 of Perciformes

With more usual body plans???

Response: Thank you for your suggestion. We revised this phase into "of Perciformes with regular body plans" in the revised manuscript.

94 added one more family (Scophthalmidae, Pleuronectoidei), and 80 transcriptomes It would be more useful to say 'transcriptomes from an average of x organs from y species' or something to indicate breadth. 80 transcriptomes of 2 organs from one species would not be very helpful.

Response: Thank you for your suggestion. We have added more detailed information of how we implement the transcriptomes analysis, including how many species and organs we selected by adding "80 transcriptomes (including 72 from three tissues of *Paralichthys olivaceus*; 4 from two tissues of *Platichthys stellatus*; 2 from two tissues of *Toxotes chatareus* and 2 from two tissues of *Polydactylus sextarius*) we generated" in lines 86-88, page 3 of the main text.

97 adaptation of flatfishes versus their ancestors; and 2) the genes THAT EXPERIENCED significant

Response: Corrected as suggested.

103 Using [the DELETE] whole-genome sequencing strategies

Response: Corrected as suggested.

109 species, spiny turbot (Psettodeserumei), spotted archerfish (Toxoteschatareus), and pacific
110 threadfin (Polydactylussextarius),
Tell your readers which are flat fish and which not. Also, justify why these two specific non-flatfish. The tree makes it clear, but it should be in the text.

Response: Thank you for your suggestion. We have revised the sentence into "Among them, three species with controversial phylogenetic status, including spiny turbot (*Psettodes erumei*, Pleuronectiformes), spotted archerfish (*Toxotes chatareus*, Perciformes), and pacific threadfin (*Polydactylus sextarius,* Perciformes), were sequenced......" in lines 99-102, page 4 of the main text to clarify this point.

112 genomes of three species, starry flounder (Platichthysstellatus), spotted archerfish (T.
113 chatareus), and pacific threadfin (P. sextarius),
You don't need to give both common and Latin names twice.

Response: Thank you for your suggestion. We have deleted the common names here and revised this sentence into “genomes of three species, *Platichthys stellatus*, *Toxotes chatareus*, and *Polydactylus sextarius*, were assembled into the chromosome-level....”.

119 scores (Supplementary Tables 31-40; see Supplementary Note 10), READ mapping ratios
120 (Supplementary Table 41; see Supplementary Note 10), and TRANSCRIPT mapping ratios

Response: Corrected as suggested.

938 Geographical distribution of the species sequenced in this study. The DARK green area on The legend should tell reader what the purple ovals are.

Response: Thank you for your suggestion. The purple ovals in Fig. 1a were previously supposed to represent the global distribution regions of all the sequenced species in this study. But considering that the rest images of Fig. 1a have provided the more detailed and accurate information of the real global distribution regions of each species, we deleted these purple ovals, but instead show all the species in the right place to give the overall distribution information in first panel in the revised manuscript.

939 the map represents the global distribution regions of each species. The blue, red, and gray 940 circles
It would be so much easier to use L for left, R for right and B for both sides. The colors make your reader work too hard to learn a meaningless code.

Response: Thank you for this great suggestion. We have revised the legend of Fig. 1a and used L to represent left, R for right, and LR for left and right in the figure to indicate the eye phenotypes of these fishes sequenced in this study.

940 circles next to the fish represents species that have left-SIDE eyes, right-SIDE eyes, or ONE EYE ON EACH SIDE of the body),

Response: Corrected as suggested.

946 indicates the size of the DNA elements, LINE, SINE
The size of each element? Some LINES are longer than others. Or the fraction of the genome of each species that consists of LINEs etc?

Response: Sorry for the ambiguity. Here we refer to the actual assembled size of each element, including coding regions, DNA elements, LINE, SINE, LTR and other genomic regions, in each species.

We have revised this sentence into "Diagram indicates the size of each type of elements including the coding regions, DNA elements, LINE, SINE, LTR and other genomic regions in each species".

Fig. 1e – it would help connectivities to have the same chromosome nomenclature across the entire clade.

Response: Thank you for your suggestion. We have updated the Fig.1e and used same chromosome nomenclature across the entire clade in the revised manuscript.

130 considerable PROPORTION of

Response: We have deleted this description about repetitive sequences content among species, according to the suggestion by reviewer#1 in case that the observed values of repetitive sequences content might have been influenced by different sequencing strategies.

133 THAN the P. dupliocellatus genome

Response: We have deleted this description, as above one.

141 By combining our ten de novo assembled genomes with eight published GENOME SEQUENCES from

Response: Corrected as suggested.

.

151 of suborder Psettodoidei formed one clade with Perciformes species WITHOUT FLAT BODIES, T. chatareus and

Response: We have revised it into "......of suborder Psettodoidei forms one clade with the two Perciformes species with regular body plan, *Toxotes chatareus* and......".

154 GENE TREES and SPECIES trees P. erumei is clustered with non-flatfish

Response: Corrected as suggested.

155 lineage rather than with Pleuronectoidei species provided strong supports for independent 156 origins of Pleuronectoidei and Psettodoidei from lower-percoids.The other possibility is that the last common ancestor of both groups evolved a flat body plan that was secondarily lost in T. chatareus and P. sextarius. Text needs to present arguments to distinguish these two hypotheses.

Response: Thank you for raising this intriguing alternative explanation for our observations. As explained above, we have added the possibility of multiple losses after the discussion of two independent evolutionary events. Given the current data and evidence, the independent origin hypothesis seems more likely. Firstly, from the point view of evolutionary biology, multiple losses along a lineage are less parsimonious (Hillis,
1990, *Sinauer Associates In. Publication*). Considering that *Toxotes chatareus* and *Polydactylus sextarius* form independent sub-lineages in the Psettodoidei lineage rather than forming sister group to the *Psettodes erumei*, such multiple losses along the lineage is less likely according to parsimony principle of evolution. Herewe used only *Toxo*tes *chatareus* and *Polydactylus sextarius* as the outgroup of the Psettodoidei lineage, if more outgroup species such as *Sphyraena argentea, Centropomus armatus, Coryphaena hippurus, ematistius pectoralis* and many other perciforme species which have also been included in Psettodoidei lineage as indicated by previous phylogenetic studies (Li et al, 2009, *Mol. Phylogen. Evol.*; Betancur-R et al, 2013, *PLoS Curr.*), were involved, the loss events would be more and more. The chance of such large scale of multiple losses along a lineage is quite slim. Secondly, intrigued by your proposal we looked at some body plan genes in the lineage of *Toxotes chatareus*, *Polydactylus sextarius* and *Psettodes erumei*, we observed majority of body plan genes in *Toxotes chatareus* and *Polydactylus sextarius* have the same mutations with perciformes instead of real flatfishes (Pleuronectoidei) (Supplementary Table 80). It is less likely that so many perciformes-like mutations in *Toxotes chatareus* and *Polydactylus sextarius* are consequences of reverse mutations according to the evolutionary principle that reverse mutations are rarely happened (Li, 1997, *Molecular Evolution*). In this context, the multiple losses hypothesis seems less likely. We have added these new results and discussions about the two possible hypotheses in lines 138-148, page 5 of the main text.

166 first time, our results provided solid perspectives on the FLATFISH ORIGINS that has been

Response: Due to stringent space limit of *Nature Genetics*, we deleted this sentence.

172 With [the DELETE] fossil calibration

Response: Corrected as suggested.

173 approximately 76.1 and 80.0 million years ago (Ma), respectively IN THE late
Cretaceous
I'm not qualified to critique this assertion. I hope someone with skills in that area is helping the authors get this correct.

Response: Thank you for your concern about this point. We consulted experts and confirm that the time calibrated for origin of flatfishes is correct and is highly consistent with the time calibrated from other studies using multiple nuclear loci (Campbell, et al, 2013, *Mol. Phylogenet. Evol*). The estimation

of divergence time was analyzed using the PAML software (Yang, et al, 2007, *Mol. Biol. Evol.*) and calibrated with fossil data, which is the classical method currently widely used in genomics studies(Lin et al, 2016,*Nature*; Zhang et al, 2017, *Nature*). The time deduced for the first emergence of the Pleuronectoidei and Psettodoidei at approximately 76.1 and 80.0 million years ago (Ma), which fall right into the time range of Late Cretaceous (99.6-65.5Ma, Goswami et al, 2011, PNAS; GowerJ. et al, 2016, *J. Biogeogr.*).

174 Our time estimates were highly consistent with the time CALIBRATION by a previous Response:

Corrected as suggested.

176 loci12,28. The late Mesozoic to early Cenozoic period, which includes THE Cretaceous Response:

Corrected as suggested.

global peak pulse of fast seafloor spreading, which resulted in 182 a high stand of sea levels and
183 widespread epicontinental seas33.
Some of this repeats needlessly stuff just before.

Response: We apologize for the redundant presentation here. We have abbreviated this sentence into "The period of 80-75 Ma during the late Cretaceous experienced a peak of such a global change".

185 and hence the eventual origin of both RFPs and FLP lineages.
Where in the world did these two lineages evolve? Were they sympatric?

Response: Thanks for raising this important question. We have dug literature about the global seafloor spreading and we found that the seafloor spreading is a global event during the mid to the late Cretaceous, although pacific is the most predominant (Seton et al, 2009, *Geology*; Larson, 1991, *Geology*). In addition, if we look at the distributional pattern of flatfishes, there are around 600 flatfish species in the world and they distribute widely in all oceans throughout the world (Li et al, 1995, *Fauna Sinica: Ostichthyes Tleuronectiformes, Science press*), although again pacific recorded most of the species (Li et al, 1995, *Fauna Sinica: Ostichthyes Tleuronectiformes, Science press*). Therefore, based on these pieces of information, it is hard to make a conclusion in which ocean these two lineages firstly evolved and whether they evolve sympatrically or allopatrically.

192 Tables 80 and 81), which may explain why they EXHIBIT a "simply an asymmetric percoid

Response: Corrected as suggested.

194 strong selection pressure they experienced34
How do you know that the rapid rate was not just due to very small population sizes ?

Response: We apologize for hurriedly moving to make the conclusion that the higher evolutionary rates in both RFP and FLP indicated strong selection pressure they experienced. Other factors such as small population size and random drift could also play important roles. Therefore, we have revised this sentence here into "The higher relative evolutionary rates in both RFP and FLP indicate possible selection pressure they experienced, though other factors such as limited population size and rapid drift could also not be excluded (Kimura et al, 1971; *J.Mol.Evol.*)". 197 Genes UNDERGOING significant alterations in flatfishes

Response: Corrected as suggested.

201 FLP, Perciformes and other non-flatfish species. We analyzed [the DELETE] changed gene families in the
What does 'changed gene families' mean? Gene families whose individual proteins had rapid rates of sequence evolution? Or gene families that changed rapidly in gene number? Ambiguous.

Response: We apologized for these unclear descriptions. The changed gene families mean the gene families that changed rapidly in gene number. We have revised this description into "We first analyzed gene families that changed rapidly in gene number during the evolution process" in the revised manuscript.

208 identified genes UNDERGOING positive

Response: Corrected as suggested.

209 lineage-specific mutation (LSGs) in both RFPs and FLP
This statement implies that you ruled out genes that experienced lineage specific mutation in only one of the two lineages. If you did that, then you would miss genes that changed in one flat fish lineage but not in the other and so could miss important causative agents.

Response: We apologized for these misleading descriptions. We did identify lineage-specific mutations in RFPs and FLP separately. We have revised the sentence into "we further identified genes undergoing positive selection (PSGs), or rapid evolution (REGs), or containing lineage-specific mutations (LSGs), or lineage-specific conserved non-coding elements (SCNEs) in RFP and FLP, respectively." in the revised manuscript.

non-coding elements (SCNEs) around the neighbouring regions 212 that potentially COULD RESULT in

Response: Due to stringent space limit of *Nature Genetics*, we deleted this sentence.

214 The enrichment categories of top candidate genes under significant alteration in both RFP [NOT PLEURAL]
Response: Corrected as suggested.

223 discovered for the first time TO BE ASSOCIATED WITH RAPID SEQUENCE EVOLUTION DURING AN evolutionary transition from THE water column to seafloor colonization.

Response: Corrected as suggested.

225 this process may not only involve a cardiac morphological reorganization RESULTING from

Response: Corrected as suggested.

227 involve cardiac functional remodeling RESULTING from selective

Response: Corrected as suggested.

230 21). Such structural and functional alterations of THE cardiovascular system

Response: Corrected as suggested.

231 may have contributed to their reinforced cardiac output, which is the highest [ever DELETE] known

Response: Corrected as suggested.

233 readily encountered during in BURROWING INTO THE SUBSTRATE 54

Response: Corrected as suggested.

The observed enrichment of genes associated with musculoskeletal 241 restructuring AND lipid

Response: Corrected as suggested.

a phenotype
243 that is distinct in flatfishes (Fig. 4a; Supplementary Fig. 27; see Supplementary Note 23), 244 from which the name of "flatfishes" may arise. Not needed.

Response: Thank you for your suggestion. We have deleted the phrase “a phenotype that is distinct in flatfishes (Fig. 4a; Supplementary Fig. 27; see Supplementary Note 23) from which the name of flatfishes may arise”.

952 red numbers represent the amounts of expanded and contracted gene FAMILIES in this node, Authors should add a scale bar to each image so reader can see how they vary in size as well.

Response： Thank you for this valuable suggestion. We have added the scale bar in each image in Fig.

2a, and used “families” in the revised manuscript. 953 respectively. The overall image of each species

WAS drawn according

Response: Corrected as suggested.

Fig. 3. Do the red and blue dots in 3A mean the position of the eyes? The same graphic device should mean the same thing throughout the paper.

Response: Thank you for your suggestion. The dot here indicates the corresponding value of the relative evolutionary rate for each species. We have changed the color of dots to black to avoid ambiguity.

Fig. 3a. What do the colored ovals mean?

Response: The colored ovals represent different fish groups that showed contrasted relative evolutionary rates. Using the ovals, it would be easy to see the difference in evolutionary rates between RFP, FLP, and their Perciformes ancestors. But dividing the RFP into two groups was quite misleading, therefore, we have merged these two groups into one oval in the Fig. 2b of the revised manuscript.

Fig. 3b. This graphic isn’t very helpful. It needs a device to show the size of the number so that the reader can get an impression of how gene family sizes change across the phylogeny.

Response： Thanks for your suggestion. We have changed the size of the circles according to the number of each gene family in the previous Fig. 3b so that the reader can easily find the gene number difference between species, as requested in the revised manuscript. But according to the suggestion by reviewer#1, we have moved this figure into supplementary files as the Supplementary Fig. 19.

Fig. 3c doesn’t help me much. Consider cutting it even though it is cute.

Response: Thank you for your suggestion. We have moved this figure into supplementary files as the Supplementary Fig. 26.

958 the reference species. THE arrow represents

Response: Corrected as suggested.

Fig. 4a. Error bars are too hard to see.

Response: Thank you for this valuable comment. We have changed the size of the error bars of this figure (Fig. 3a in this revision) to make it more visible in the revised manuscript.

971 left-right axis. All the parameters were measured in three biological replicates for each 972 species. Does that mean just three individuals for each species? Is that enough? How many species? How does this parameter depend on the age of the animal? Or its standard length?

Response: We did use three individuals for each of the six species of *Cynoglossus semilaevis*, *Paralichthys olivaceus*, *Brachirus orientalis*, *Polydactylus sextarius*, *Larimichthys crocea* and *Oryzias latipes*. All fishes were adults in similar size, collected from wild. In order to further decrease the impact of age on the value of left-right axis, we have standardized the values of left-right axis to the dorsal-ventral axis of each individual. We have revised how we standardized these values in Supplementary Note 24 of the supplementary file.

Fig 4b. The flatfish species need to be indicated by some graphic feature, like color in 3b. Fig 4b. each aa or type of aa should have its own color so we can see easily how much variation exists at sites other than the one that the authors want to call our attention to.

Response: Thank you for this important suggestion. We have marked the flatfish species and non-flatfish species in different color.And we have also used different colors for aa that show substitutions in the new Fig. 3b.

245 advantage on the seafloor, where flatfishes usually [need to DELETE] hide from their enemies

Response: Corrected as suggested.

250 (PSGs, P-value = 8.43e-3), SGCA (PSGs, P-value = 3.30e-3), SGCZ (SCNEs) and DMD Tell reader what PSG and SCNE mean.

Response: Thank you for your suggestion. PSGs represent positively selected genes, SCNE represent specific conserved non-coding elements. The exact meanings of these abbreviations have been already described when they first appeared in lines 202-204 of the main text.

249 musculature development that have undergone significant alteration in RFPs, including THE SARCOLEMMA GENE SSPN
250 (PSGs, P-value = 8.43e-3), THE SARCOGLYCAN GENE SGCA (PSGs, P-value = 3.30e-3), SGCZ (SCNEs) and THE DYSTROPHIN GENE DMD
Response: Corrected as suggested.

252 see Supplementary Notes 20 and 22). Unexpectedly, all FOUR OF these genes are

Response: Corrected as suggested.

249 musculature development that have undergone significant alteration in RFPs What does 'significant alteration'mean? Alteration in what parameter? Be specific.

Response: We apologized for the previous ambiguous description. The word "significant" is not suitable here because it relates to no statistical parameter, we have changed it into "marked alteration" in the revised manuscript.

256 development of muscular tissues58-60. Mutations or abnormal EXPRESSION of these four genes

Response: Corrected as suggested.

260 that caused either structural or signaling modifications hampering normal musculature 261 development
The problem with this argument is that these all have to do with sarcomere structure, not the evolution of flatness. Flatness has to do with rib shapes and internal organ organization – you could have totally normal sarcomeres and be flat. The authors would have to show 1) that the sarcomeres of flatfish are different from 'normal ' fish in a way that's related to these proteins, and 2) that that difference is actually important for changing the shape of the fish. The point is that the thickness of the body wall, including the muscles, is not the primary factor in making the fish body flat. At least the authors have not shown that.

Response: Thank you for helping clarify this point. We agree that rib shape and internal organ organization are the factors that influence the flatness of fish body, although some documents also suggest an involvement of muscle development and their related gene expression in shaping body morphology in several fish species (Pavey et al, 2011, *BMC Ecology*; Ostberg et al, 2015, *Plos one*).

We have toned down the statement by rephrasing this sentence into "......thus may have implications in their thinner musculature and flat phenotype." in line 256, page 9.

263 (sarcoglycanopathies) in humans throughout the world67 I would have thought that DMD was.

Response: We apologize for the vague phrase. Both mutations of *DMD* and *SGCs* genes can cause severe muscular dystrophy in human. Sarcoglycanopathies is one type of the muscular dystrophy caused by mutations of Sarcoglycans genes, such as *SGCA*, *SGCB*, *SGCD* and *SGCG* (Liang et al, 2016, *J. Neurol. Sci.*). *SGCA* mutation is the most important gene causing sarcoglycanopathy (Liang et al, 2016, *J. Neurol. Sci.*). In order to avoid this ambiguity, we have changed this sentence into: "*SGCA* gene has been the most frequently reported locus that causes the majority of sarcoglycanopathies (one of the severe muscular dystrophy) in humans (Liang et al, 2016, *J. Neurol. Sci.*)".

265 mutations were mapped very CLOSE to mutations associated with THE SYNDROMES of limb-girdle

Response： We have deleted this description according to the suggestion by reviewers #1 because it seems not reasonable such mutations in the flatfish genome are equivalent to the known mutations in the human genome which are associated with the human phenotype.

268 CDC2 phosphorylation site (Fig. 4b) in SGCA, which has Have such mutations been observed in human patients?

Response: Sorry for such imprecise description. We have checked reports about the human sarcoglycanopathy caused by *SGCA* mutations. We found that dozens of mutations in this gene has been observed to cause hampered musculature development and severe muscular dystrophy such as limb-girdle muscular dystrophies (LGMD) syndrome in humans, suggesting a possible role of this site mutation in the specialized musculature of flatfishes. But no case was recorded to specifically account for the phosphorylation of these mutated sites. Therefore, we have deleted this sentence in the revised manuscript.

signal-dependent-activation profiles of muscular development 270 in RFPs that led to hampered
271 musculature and hence their flat phenotype.
Again, authors assume without proof that the musculature is the main reason that the fish are flat.

Response: As indicated above, we actually don't have proof to attribute the flat phenotype mainly to the hampered musculature. We have toned down the statement by revising this sentence into "Such

alterations in *SGCA* may change the signal-dependent-activation process of muscular development in RFP and thus may have implications in their thinner musculature and flat phenotype".

276 signals73,74 essential for ADIPOGENESIS

Response: Corrected as suggested.

277 Mutations or 277 abnormal expressions of MEX3C and MLX would result in reduced adiposity
Zebrafish has an A at this position and flatfish have a G. These are conservative changes.
What evidence is there to support the claim here that this specific mutation would result in ××reduced adiposity?
Fig. 4e shows that flatfishes differ from the other fish shown in crude fat, but that in no way means that this mutation in this gene is responsible.

Response: Yes, we apologize for the narrating manner by hurriedly moving to assuming these mutations would affect protein function without further evidences. Actually, both *MEX3C* and *MLX* are rapidly evolving genes in RFP and there is no fixed amino acid substitution between flatfishes and outgroups. We have abbreviated the description about *MEX3C* and *MLX* in lines 262-264, page 9 in the main text. In addition, as described above, in order to provide evidence that these observed mutations in lipid-related genes would probably correlate with reduced adiposity, intrigued by you, we further conducted *in vitro* enzyme catalytic activity assay for BBOX1.The results show that RFP-specific BBOX1 has significantly higher (P-value < 0.01) activity catalyzing the formation of L-carnitine from gamma-butyrobetaine (Fig. 3d), indicating higher carnitine production in RFP. It is well-known that L-carnitine is a molecule critical in lipid oxidation (Zhao et al, 2020, *Gastroenterology*).Therefore, this result indicates that the RFP *BBOX1* may at least partially account for the low fat accumulation phenotype in RFP.

982 were measured in three biological replicates for each species
What does biological replicate mean? Three different samples from one individual? If so, this is not meaningful. Also, sex is not given but sex and stage of reproductive cycle/season of the year affect this parameter. Were these the same in all species tested?

Response: We apologize for not clearly describing the measurement. Three biological replicates represent three individuals for each species. It is true that reproductive cycle and season may affect the lipid content of the teleost fish. When we were conducting this measurement, to decrease the impact of these factors, we collected all fish samples from the wild at the same season in December of 2018. But we have to confess that we did not check the sex. In the revision, we have clearly described these facts and added the information such as sampling season, reproductive status, fish size in Supplementary Note 24 of the supplementary file to show how we analyzed the lipid content for

these species, and further cite more references which observed flatfishes have the lower lipid content than other teleosts to further support this point (Schloesser, et al, 2017, *T.Am.Fish.Soc.*; Wander, et al, 1991, *J. Food.Cpmpos.Anal.*) in the main text.

285 FOLD significantly lower.

Response: Corrected as suggested.

286 muscular tissues respectively in RFPs than in [other DELETE] non-flatfish species

Response: Corrected as suggested.

292 been adopted by both RFPs and FLP during the evolution of their flat body plan. TAKEN

Response: Corrected as suggested.

293 together, our analyses provided the first piece of evidence supporting the role of hampered
294 development of musculature induced by altered DGC components, coupled with a restricted
295 lipid accumulation in evolution of the body flatness of flatfishes
These studies do show that flatfish have altered lipid biology but do not show how that phenotype is associated with flatness. Fig. 4e does not make that connection.

Response: Thank you for point out this important issue. Body lipid content is an important factor that influences the fat or lean morphs of teleost fish species (Goetz et al, 2010, *Mol. Ecol*; Hansen et al, 2016, *Hydrobiologia*), with low lipid content usually correlate with thin body wall (Eschmeyer et al, 1965, *T. Am. Fish. Soc.*) and lean phenotype (Goetz et al, 2010, *Mol.Ecol*; Hansen et al, 2016, *Hydrobiologia*). We observed low lipid content and a lean flat body in flatfishes (Fig. 3e; Supplementary Figs. 28 and 29). These results suggest that the less lipid content in flatfishes may partially correlate with their lean and flat morphs. Considering other factors such as the rib shapes and internal organ organization may also influence the body flatness, as you have pointed out. Therefore, we revise our description here by not attributing the body flatness solely to lipid profile, and toned down the causal relationship between them by describing "Taken together, our analyses provide the first piece of evidence that marked changes have been undergone in musculature development and lipid accumulation genes in flatfishes, and thus may correlate with the evolutionary origin of their body flatness."

301 genetic basis remains largely unknown since Darwin's time8. RECENTLY,ADVANCES HAVE BEEN MADE UNDERSTANDING the genetic
302 regulation of body plan asymmetry in animals.

Response: Corrected as suggested.

306 THE "NODAL-PITX2 signaling cassette

Response: Due to stringent space limit of *Nature Genetics*, we deleted this sentence.

312 WNT9B (LSGs, L188M), SFRP5 (LSGs, K236R), TPBG (PSGs, P-value = 8.02e-4), 313 POU2F1 (
It is not possible to know the significance of these changes without additional data. 1) do they change the function of the proteins in some way relevant to body flattening? 2) lineage changes in many genes occur by chance in every large taxon, why do the authors focus on these? Are these the only lineage specific changes in the genome? For these genes, the authors don't go from unbiased look at all lineage specific function-changing mutations to see what they are involved in, but instead, take the biased approach of looking at their favorite genes and seeing if they have changes. 3) What does the P value mean? What is being compared to what?

Response: Thank you for raising the important questions. Yes, just as you have pointed out, it is hard to know the exact function of these changes without additional data. We might not have clearly explained this result. At the current stage, we were only able to add enzymatic experiments for two enzyme-coding genes, and more functional characterizations on other genes await other independent projects. The genes highlighted with significant evolutionary signals in this study provide a guide list for future functional characterizations.

Regarding the four genes, we didn't take the biased approach of looking at our favorite genes and seeing if they have changes. We actually have taken a unbiased approach to find out all the PSGs, REGs, and LSGs, that usually conferred important information correlated with lineage specific traits, following the traditional comparative genomic analysis (Lindblad-Toh et al, 2011, *Nature*; Qiu et al, 2012, *Nat. Genet.*) . There are not too many such kind of genes, and all these genes have been provided in Supplementary Tables 90 and 96 of the supplementary file. Among these genes, the four, *TPBG*, *POU2F1*, *WNT9B*, and *SFRP5*, are related to body development (Juriloff et al, 2014, *Birth Defects Res.*; Wu et al, 2017, *Dev. Cell.*), and they all are the core components essential for *WNT* signaling transduction, where WNT9B act as ligand and POU2F1 serving as an positive activator of *WNT* signaling pathway. The alterations of these key *WNT* signal pathway genes may indicate their roles in the body plan asymmetry of flatfishes.

The *P* value represents statistical significance in χ^2-test that was used to check whether $\omega 2$ was significantly higher than $\omega 1$ and $\omega 0$ under the threshold *P*-value < 0.05, suggesting that these genes may be under positive selection or fast evolution. Here $\omega 2$, $\omega 1$, $\omega 0$ represents the ratio of the rate of non-synonymous substitutions to the rate of synonymous substitutions between sequences calculated using branch and branch-site models in the codeml program of the PAML package (Yang, et al, 2007,*Mol. Biol. Evol.*). The sequences of RFP lineage were compared with outgroup (*Larimichthys*

crocea, *Labrus bergylta*, *Oreochromis niloticus*, *Oryzias latipes*, and *Danio rerio*) when the branch and branch-site models were implemented.

In order to provide more evidence for the possible functional significance, we also checked if the observed amino acid mutations would change the physicochemical property of the residues, and the results showed that most of these mutations in these peptides do have obvious physicochemical effects. In addition, the 3D structures of SFRP5 can be successfully modeled and the results show that there are obvious structure changes caused by the mutations (Supplementary Fig. 24). We have added these additional data and cautiously toned down our statements to avoid misunderstanding that we were claiming causal relationship between these genes' mutations and asymmetric body plan of flatfishes by these sentences: "Taken together, our analyses provide gene evolution and expression evidence for the possible involvement of *WNT* combined with *RA* signal pathways in shaping the asymmetric body plan in flatfishes for the first time (Fig. 4f), though the exact role of these *RA* and *WNT* genes in the body plan asymmetry still awaits further investigations" (lines 352-355, page 12).

329 the genetic variation in these genes may point to a role of RA signaling 329 in the left-right body Only if authors demonstrate that these specific amino acid changes they observe in fact alter the protein functions in a way known to affect body symmetry.

Response: Thank you for this valuable suggestion. As you suggested, we conducted experiment to test if flatfish specific amino acid changes in RDH14 would affect its enzyme activity. The *RDH14* gene catalyzes retinaldehyde into retinol. Our result shows that the RFP RDH14 enzyme has 2.51 fold lower (*P*-value < 0.01) catalytic activity than that of outgroups , implying more retinaldehyde (substrate for RA synthesis) accumulation and thus *RA* signaling changes in RFP. RA has been proved to be a critical factor in the induction of asymmetric body plan in flatfish (Shao et al., 2017, *Nat. Genet.*). Such RA signaling alterations in RFP may be an adaptive signal to this drastic turnover of body plan program. Furthermore, our transcriptome and qPCR analysis further revealed that many of these *RA* signaling genes exhibited an obvious transient expression fluctuation in flounder tissues correlating with the metamorphosis (Supplementary Fig. 34), further predicting their involvements in development of their asymmetric body plan of flatfishes. These results provide evidence that these changes in *RA* signaling may possibly play a role in development of asymmetric body plan of flatfishes.

332 PATHWAY genes that have undergone

Response: Corrected as suggested.

337 Our transcriptomic data analyses lend further SUPPORT to the INVOLVEMENT of WNT

Response: Corrected as suggested.

representative, we showed that multiple genes in both RA (ALDH1, ALDH8, RDH5, RDH7, RDH8, RDH11, RDH12, RDH13) and WNT (WNT1, WNT4, and WNT10) signal pathways exhibited a significant left-right asymmetrical expression in three examined flounder tissues These observations are interesting but to reveal mechanisms, we need to know that 1) these genes are not asymmetrically expressed in closely related bilaterally symmetric species, and 2) that these are among the most significantly differentially expressed genes left vs. right, and 3) that the species with both eyes on the left have one way of asymmetry and the species with eyes on the right exhibit the opposite direction of asymmetrical expression.

Response: Thank you for pushing to clarify these problems. We confess that we are not presently able to directly compare the gene expression profile of flatfishes with other non-flatfish species because of the difficulties in defining exact correspondent developmental time windows between the two groups, especially during post-embryonic development when classic/marked events characteristics of certain developmental time window are no longer easily recognized in these non-model fishes. However, it is very feasible for us to compare the gene expression profile among different developmental time windows in a flatfish species according to the time sequential of development. From our transcriptome analyses, we observed a general marked fluctuation of gene expression profiles in many *WNT* and *RA* signaling genes during the metamorphic development of flatfishes, with asymmetrical gene expression begins in pre-metamorphic larva, becoming full asymmetry during pro-metamorphic and metamorphic climax, and then back to symmetrical during post-metamorphosis. That means that these gene expression became asymmetry during a very narrow time window correlate with metamorphoric process in flatfishes. In addition, as you pointed out, many of these genes are indeed the most significantly left vs. right differentially expressed genes (Supplementary Fig. 34). Such obvious asymmetric signaling in larva stage much later after the early embryonic somite period, is not usual in teleost with regular body plan and in other vertebrate (Suzuki et al, 2009, *Dev.Growth & Differ.*; Schweickert et al, 2018, *J. Cardiovasc. Dev. Dis.*). Therefore, these differentially expressed genes correlate with metamorphoric process may possibly play a role in driving the metamorphosis and hence the body plan asymmetry in flatfishes. Based on your comments and above facts, we have improved the description by 1) verifying the fluctuation of some gene expression using the qPCR and add the fluctuation profiles of these gene expression in Supplementary Fig. 34; 2) clearly describe that these genes'expression fluctuates during metamorphosis and the fluctuation is coincident with the metamorphic event; 3) adding discussion about the expression profile of body axis related genes during post-embryonic stage in teleost fish species with normal body axis to further support the possible involvement of these genes in metamorphic event and hence the asymmetrical development of body plan in flatfishes.

metamorphosis. This was again supported by the evidence that the left deviation of expression of pigmentation genes, such as TYR101, MITF101, and TYRP1101 usually occurs after the

351 asymmetrical expression of RA and WNT signals in the skin of metamorphosing flounder Yes, this is a good observation supporting the authors'position

Response: Thanks for your positive comments.

352 larvae (Figs. 5c,d). Interestingly, significant left-right asymmetric expression of NODAL 353 signaling genes (including NODAL, LEFTY, and PITX2) was also observed in the tissues of These genes are also asymmetrically expressed in 'symmetric' species like medaka and zebrafish.

Response: We apologize for the unclear description here. Yes, at the very early stage of embryonic development, i.e. during early somite stage of vertebrates including fishes, it is common that *NODAL* signaling are usually left-right asymmetrically expressed in embryonic node thus providing the cues for establishing the left-right axis and hence promoting the left-right asymmetrical development of many important organs (Montague et al, 2018, *Development*). In contrast, *NODAL* genes were founded to be again reactivated in the metamorphosis stage much later after somite period in flatfishes (Suzuki et al, 2009, *Dev. Growth & Differ.*). Now it is believed that such *NODAL* signaling reactivation in metamorphic larvae is related to the asymmetrical body plan development in flatfishes during metamorphosis (Suzuki, et al, 2009, *Dev. Growth & Differ.*; Schreiber et al, 2013, *Curr. Top. Dev. Biol.*). To make this description clearer, we changed this sentence into "Left-right asymmetric expression of *NODAL* signaling genes (including *NODAL*, *LEFTY*, and *PITX2*) was also observed in the tissues of metamorphic flounder larvae (e.g. muscles and eyes) (Fig. 4d). Such obvious reactivation of *NODAL* signaling in metamorphosis, usually not observed in teleosts with regular body plan, is believed to have initiated the left-right asymmetry of flatfishes".

357 asymmetrical expression of RA and WNT signals. Although obvious cross-TALK between

Response: Corrected as suggested.

371 analysis, when we measured the dorsal, anal, pectoral and pelvic FIN length of flatfish species

Response: Due to stringent space limit of *Nature Genetics*, we deleted this sentence.

386 indispensable for specification of the zone of polarizing activity (ZPA)102. Yes, true, but 1) this is for paired fins, not the dorsal and anal fin that enact the fin-feet walking, and 2) the K to R substitution is a conservative change. Where is the evidence that this would cause a change in protein function? 3) is this hoxd12a or hoxd12b? 4) I don't see a K at position 105. 5) the outgroups the authors chose to present all have K at this position, but is this the only lineage among all teleosts or all vertebrates that has R at this position for both hoxd12a and hoxd12b?

Response: Thank you very much for pushing to clarify these problems.

1) Sorry for having not clearly described this issue. Yes, as you pointed out, *HOXD12A* gene was found to function in the development of paired fins in many teleosts (Small et al, 2016, *Genome Biol.*; Crow et al, *Front. Ecol. Evol.*), and it is also found to play roles in the development of unpaired fins such as dorsal fin in flounders (Chen et al, 2017, *Gene Expr. Patterns*), as we cited in lines 375 of the main text. In addition, paired fins are also important for enacting a fin-feet walking, because it not only serves as a rotation point to help keep an accurate maneuvering orientation (Fox et al, 2018,*Zoology*), as has been described in lines 364-366, but also gives an added thrust to movements for some flatfish species (Orcutt, 1950,*California Department of Natural Resouces Division of Fish and Game Fish Bulletin*; Gibson et al, 2015, *Flatfishes:Biology and Exploitation. Second edition*,John Wiley and Sons,Ltd). We are sorry for not clearly describing these in the original main text. We have revised the sentence in lines 364-366, page 13 of the main text into "these specialized fins enable a repeated generation of the "fin-feet" (mainly by dorsal and anal) pushing down against the substrate to produce constant forward movement while keeping an accurate maneuvering orientation (mainly by pectoral)" in the revised manuscript to clearly describe this context.

2) Yes, we are not presently able to provide sufficient evidences that this mutation would influence the function of *HOXD12A*. We have toned down our statement about the role of *HOXD12A* in the development of the specialized fin of the flatfishes in the revised manuscript as "The observed mutations in *HOXD12A* may have implications in the morphological changes of median and paired fins in RFP, though the causative effect of these mutations still awaits further verification".

3) Yes, this is *HOXD12A* gene. We have revised all the "*HOXD12*" into "*HOXD12A*" throughout the revised manuscript.

4) This mutation site (105) was based on the peptide sequence of the *Platichthys stellatus HOXD12A*, as shown in Supplementary Fig. 27. Therefore, the 105 position aa may not be seen in other species. This equivalent mutation site can only be observed through homologous alignment using software.

5) It is difficult to extend this check to all teleosts or all vertebrates because there are huge quantities of *HOXD12A* sequences in the databases. Intrigued by your suggestion, we have downloaded some *HOXD12A* sequences of teleost species to compare, including *Ictalurus punctatus* (NM_001200975.1), *Luciobrama macrocephalus* (GU218388.1),

Culter alburnus (GU218385.1), *Ochetobius elongatus* (GU218387.1), *Opsariichthys bidens* (GU218386.1), *Hypophthalmichthys molitrix* (GU218383.1), *Ctenopharyngodon idella* (GU218382.1), *Mylopharyngodon piceus* (GU218381.1), and *Squaliobarbus curriculus* (GU218384.1), from NCBI and performed multiple sequence alignment analysis. At the sites homologous to the 105 site of *Platichthys stellatus*, none of them is the amino acid "R", but they are all the amino acid "K" except for *Ictalurus punctatus* in which it is an amino acid "G". This might indicate that at least in teleosts, amino acid "R" at this site are not usual. But as you pointed out that there is still no solid evidence showing this mutation would influence the function of *HOXD12A*. Therefore, we have toned down our statement about the role of *HOXD12A* in the development of the specialized fin of the flatfishes in the revised manuscript as "The observed mutations in *HOXD12A* may have implications in the morphological changes of median and paired fins in RFP, though the causative effect of these mutations still awaits further verification".

392 of lysine105 to arginine105 in HOXD12, This
statement repeats info from above.

Response: Sorry for the problem. We have changed this sentence into "The observed mutations in *HOXD12A* may have implications in the morphological changes of median and paired fins in RFP" in the revised manuscript.

Suppl table 96 needs to give the accession number for each of these proteins, otherwise, how will reader be able to know what amino acid authors really mean, as illustrated by my problem with Hoxd12.
Throughout, the P as an abbreviation for the species is insufficient because it makes P. erumei and P. blochii and P. olivaceus all appear to be in the same genus. Ps. erumei and Pa. olivaceus, for example would help the non specialist.

Response: Thank for your valuable suggestion. We have added accession number for each protein in Supplementary Table 97.

And we also have unified the nomenclature of genus name for each species throughout the main text and supplementary files. We noticed that for some species, for instance, *Paraplagusia blochii* and *Paralichthys olivaceus*, it takes at least five alphabets to distinct genus *Paraplagusia* and *Paralichthys*, and it may take two or three for other species. Therefore, to keep clear, we finally decided to use full Latin names for each species throughout the manuscript in the revision.

411 genomics approaches to shed LIGHT on the evolutionary.

Response: Corrected as suggested.

420 Psettodoidei also exhibited unique mutation patterns in genes associated with less asymmetric body plan.
This seems to contradict that Psettodeserumei is asymmetric rather than symmetric. OK, I see, it's less asymmetric than flounder but more asymmetric than other percoids. This sentence should be revised so not to confuse.

Response: Thank you for your suggestion. We have revised this sentence into "Psettodoidei also exhibited unique mutations that may contribute to their less asymmetric body plan compared to Pleuronectoidei" to make this clearer. 422 the phylogeny of flatfishes, while the genes highlighted in this study LAY a solid

Response: Corrected as suggested.

443 maculatus, C. lugubris, B. orientalis, P. blochii, C. nudipinnis, P. dupliocellatus, and P. As far as I could tell, the genera of many of these was given only in the 'data availability' section. The rule is that the first time a species is mentioned, it has to be the complete name.

Response: Thanks for your suggestion. We used the full name for each species throughout the manuscript.

456 species of P. stellatus, T. chatareus, P. sextarius, and P. olivaceus, the cDNA libraries were The text does not say what organs or tissues were taken for study, even when these data are discussed.

Response: We apologize for the ambiguous description. We have added the detailed information on what organs are used by revising this sentence into "the cDNA libraries were constructed from RNA extracted from various tissues such as eye, liver, muscle, and skin, as indicated in Supplementary Table 2 for different analyzing purposes ...." in the main text in the revised manuscript.

512 Identification of orthologous genes. ORTHOLOGS were identified

Response: Corrected as suggested.

522 best similarity pairs among species were considered as putative orthologs This is good, but a comparison of conserved syntenies would be better, especially not to confuse the 'a'and 'b' copies from the teleost genome duplication。

Response: Yes, we have carefully checked whether these putative orthologs are conserved syntenies using MCscan software (Tang et al, 2008, *Science*) after the genome-wide alignment. The results

showed that the putative orthologs identified matched well with the conserved syntenies in these species. We have added this in the Materials and Methods section in the revised manuscript by describing "....and the reciprocal best similarity pairs among species were considered as putative orthologs after further evaluation using MCscan software (v0.9.13)". Thank you for your suggestion.

524 Phylogenetic tree construction and divergence time evaluation. All the single-copy genes Tell the reader how many genes that is.

Response: Thank you for the suggestion. We have revised this sentence into "All the 1,693 single-copy homologous genes identified among species....."in the revised manuscript.

531 OrthoFinder (v2.3.5)21. Divergence TIMES of these species were then estimated Response: Corrected as suggested.

542 much faster evolution rate using Chi-square test. All the single-copy genes were used in these But the 'a' or the 'b' copy of duplicated genes might also be important in the evolution of flatfish traits. Excluding them from analysis will make the authors miss genes that might be important for evolution of traits.

Response: Yes, both the single-copy genes and multi-copy genes may have important functions in development and evolution. But to avoid the noise aroused by paralogous copies, we chose solely single-copy genes (orthologs) to more stringently guarantee that we estimate the real evolutionary rates between species, as in the routine practice of molecular evolution analysis (Li, 1997, *Molecular evolution*, *Sinauer Associates Inc publication*).

562 IDENTIFICATION of genes

Response: Corrected as suggested.

564 single copy genes among species were manually checked and
So did you exclude gene duplicates from the teleost genome duplication? Be clear.

Response: Yes, we did exclude the gene duplicates in this analysis and clearly explained that we only used single copy genes in the "Identification of genes with lineage-specific mutation" section of Materials and Method.

574 Identification of conserved non-coding ELEMENTS. Using

Response: Corrected as suggested.

575 the genomes of other species were aligned to the reference genome using Which other species? Which genome was the reference genome?

Response: We apologize for the unclear presentation. We have revised this sentence into “Using *Platichthys stellatus* genome as the reference, the genomes of flatfish and outgroup species were aligned to the reference genome using LAST software” in the revised manuscript.

587 The transcripts were assembled and gene expression values were analyzed using the cufflinks Cufflinks is inadequate. The authors should have used DESeq2 because it gives a much better statistical treatment. I think it might be because DESeq2 works best with 4 or more replicates but they have just 3 ‘biological replicates’, but they don’t actually say if they come from 3 different individuals for each species.

Response: Thank you for your suggestion. We apologize for the ambiguous presentation for the biological replicates in the transcriptome analysis. As indicated above, because the metamorphic flounder larva is too small and tissue samples from one individual is far from enough for a regular transcriptome analysis, we actually collected at least 30 individuals for each sampling and repeated three times for the sampling as three biological replicates. Yes, DESeq2 method for samples from four individuals would have good statistical significance. But Cufflinks method for samples from three individuals is also widely used in transcriptome analysis (Secco et al, 2013, *Plant Cell*; Goldstein et al, 2017, *Genome Res*.). Therefore, we analyzed the gene expression profile in our case using Cufflinks method.

It is very bothersome for the reviewer when the figures do not have figure numbers on them.

Response: We have added the figure above the corresponding figure legend for the sake of convenience of the reviewers in the revised manuscript.

971 left-right axis. All the parameters were measured in three biological replicates for each972 species. Does that mean three different individuals of each species? Be clear.

Response: We apologize for the unclear description. Yes, the three biological replicates mean three different individuals for each species. We have revised the sentence into “All the parameters in 3a and 3e were measured in three biological replicates (three individuals) for each species” in lines 1010-1011, page 32 in the revised manuscript.

Fig. 4c – the exons and introns need to be marked and a scale ruler is needed across the gene.

Response: Thank you for your suggestion. We have marked the exons/introns using different colors and a scale ruler has been added across the gene in Fig. 3c in the revised manuscript.

Text does not make it clear what the take home message is for Fig 4c

Response: We apologize for the unclear description about the original Fig. 4c. We have added the detailed information about the new Fig. 3c in legend in lines 1005-1009, page 32 in the revised manuscript.

988 WNT9B gene in flatfishes compared with other non-flatfish teleosts I saw no evidence that the M to L mutation would change the protein's function.

Response: Yes, we indeed have no clear evidence to indicate the change would influence the protein's function presently. Given the fact that *WNT9B* has been involved in the craniofacial development, disruption of *WNT9B* signaling would cause severe craniofacial development disorder such as bilateral craniofacial asymmetry and skull malformation in vertebrates (Juriloff et al, 2014, *Birth Defects Res.*; Jackson et al, 2015, *Am. J. Mol. Biol.*), and M to L mutation in the cysteine-rich conserved wnt1 domain of the *WNT9B* gene, concrete role of this substitution in *WNT9B* still await further verification in future. We have toned down the description of WNT path way, in which *WNT9B* was included in the origin of specialize body plan of flatfishes by "Taken together, our analyses provide gene evolution and expression evidence for the possible involvement of WNT combined with RA signal pathways in shaping the asymmetric body plan in flatfishes for the first time (Fig. 4f), though the exact role of these *RA* and *WNT* genes in the body plan asymmetry still awaits further investigations" in lines 352-355, page 12.

989 structure was shown on the top of the graph, and the site THAT SHOWED variation was marked

Response: Corrected as suggested.

FIG 5b. Show where the exons are. Include a scale ruler in the horizontal axis.

Response: Thank you for your suggestion. We have use different colors to indicate where the exons/introns are in this figure (Fig. 4b in this revision), and we also have added a scale ruler in the horizontal axis, as requested in the revised manuscript.

Fig 5e. What is the vertical dashed line?

Response: We apologize for the ambiguity. The dashed line here was supposed to mark the stage during which the number of the specific highly-expressed genes began to show the most remarkable changes, which may indicate important events correlated with metamorphosis. But as you pointed out, the vertical dashed line can not clearly convey this notion. We therefore changed the vertical

dashed line into a “rhombus” symbol to clearly show the time point when the most remarkable changes appear, and we have also explained in the legend of the figure to account for this change.

Reference

1. Belyaeva, O.V. &Kedishvili N.Y. Human pancreas protein 2 (PAN2) has a retinal reductase activity and is ubiquitously expressed in human tissues. *FEBS Lett.*531, 489-493 (2002). 2. Betancur-R, R. &Orti, G. Molecular evidence for the monophyly of flatfishes (Carangimorpharia: Pleuronectiformes). *Mol.Phylogenet. Evol.*73, 18-22 (2014).

3. Betancur-R, R. et al. Addressing gene tree discordance and non-stationarity to resolve a multi-locus phylogeny of the flatfishes (Teleostei: Pleuronectiformes). *Syst.Biol.*62, 763-785 (2013).

4. Betancur-R R, et al. The tree of life and a new classification of bony fishes. *PLoS Curr.*5, (2013).

5. Campbell, M.A., Chen, W.J. & Lopez, J.A. Are flatfishes (Pleuronectiformes) monophyletic? *Mol.Phylogenet. Evol.*69, 664-673 (2013).

6. Campbell, M.A., Chen, W.J. & Lopez, J.A. Molecular data do not provide unambiguous support for the monophyly of flatfishes (Pleuronectiformes): A reply to Betancur-R and Orti. *Mol.Phylogenet. Evol.*75, 149-153 (2014).

7. Chen, S.L. et al. Whole-genome sequence of a flatfish provides insights into ZW sex chromosome evolution and adaptation to a benthic lifestyle. *Nat. Genet.*46, 253-262 (2014). 8. Chen, J., Liu, X.Y., Yao, X.H., Gao, F. & Bao, B.L. Dorsal fin development in flounder, Paralichthysolivaceus: Bud formation and its cellular origin. *Gene Expr. Patterns*25, 22-28 (2017).

9.Crow, K.D. Klomp J., Fisher R., & Crow K. How the Devil Ray got its Horns: the Genetic Basis of Body Plan Remodeling in Manta Rays and their Relatives. *Front. Ecol. Evol.*13, 181 (2018).

10.Liang, D., Wu, R., Geng, J., Wang, C. & Zhang, P. A general scenario of Hox gene inventory variation among major sarcopterygian lineages. *Bmc Evol. Biol.*11:25(2011).

11. Dettai, A.and Guillaume, L. Further support for the clades obtained by multiple molecular phylogenies in the acanthomorph bush. *C. R. Biol.*328, 674-689 (2005).

12. Eschmeyer, P.H. and Phillips, A.M. Fat content of the flesh of siscowets and lake trout from Lake Superior. *T. Am. Fish. Soc.* 94, 62-74 (1965).

13. Fox, C.H., Gibb, A.C., Summers, A.P. & Bemis, W.E. Benthic walking, bounding, and maneuvering in flatfishes (Pleuronectiformes: Pleuronectidae): New vertebrate gaits. *Zoology*130, 19-29 (2018).

14. Gibson, R.N., Stoner,A.W. &Ryer, C.H. The behaviour of flatfishes. Flatfishes:Biology and Exploitation. *Second edition (R. N. Gibson, ed.):John Wiley and Sons,Ltd.* 2015.

15. Goetz, F.et al. A genetic basis for the phenotypic differentiation between siscowet andlean lake trout (Salvelinus namaycush). *Mol. Ecol.*19, 176-196 (2010).
16. Goldstein, I.et al.Transcription factor assisted loading and enhancer dynamics dictate the hepatic fasting response. *Genome Res.*27, 427-439 (2017).
17. Goswami,A. et al.A radiation of arboreal basal eutherian mammals beginning in the Late Cretaceous of India.*Proc. Natl. Acad. Sci. USA*108, 16333-16338 (2011).
18. Gower, D. J. et al. The role of wet-zone fragmentation in shaping biodiversity patterns in peninsular India: insights from the caecilian amphibian Gegeneophis. *J Biogeogr*43, 1091-1102 (2016).
19. Hansen, M.J. et al. Life history differences between fat and lean morphs of lake charr (Salvelinus namaycush) in Great Slave Lake, Northwest Territories, Canada. *Hydrobiologia*783, 21-35 (2016).
20. Harada, Y., Yokota, C.,Habas, R.,et al. Retinoic acid-inducible G protein-coupled receptors bind to frizzled receptors and may activate non-canonical Wnt signaling. *Biochem. Biophys. Res. Commun.*358, 968-975 (2007).
21. Harrington, R.C. et al. Phylogenomic analysis of carangimorph fishes reveals flatfish asymmetry arose in a blink of the evolutionary eye. *BMC Evol.Biol.*16, 224-238 (2016).
22. Hillis, D.M. & Moritz, C. *Molecular systematics. Sunderland, MA: Sinauer Associates Inc.*, 1990.
23. Jackson, H.W., Prakash, D., Litaker, M., Ferreira, T. &Jezewski, P.A. Zebrafish Wnt9b patterns the first pharyngeal arch into D-I-V domains and promotes anterior-medial outgrowth. *Am. J. Mol. Biol.*5, 57-83 (2015).
24. Juriloff, D.M., Harris, M.J., Mager, D.L. &Gagnier, L. Epigenetic mechanism causes Wnt9b deficiency and nonsyndromic cleft lip and palate in the A/WySn mouse strain. *Birth Defects Res.*100, 772-788 (2014).
25. Katoh, M. Comparative genomics on Wnt3-Wnt9b gene cluster. *Int. J. Mol. Med.* 15, 743-747 (2005).
26. Kimura, M. &Ohta, T. On the rate of molecular evolution. *J.Mol.Evol.*1, 1-17 (1971).
27. Kitajima, K., Oki, S., Ohkawa, Y., Sumi, T. &Meno, C. Wnt signaling regulates left-right axis formation in the node of mouse embryos. *Dev. Biol.*380, 222-232 (2013).
28. Kyle, H. M.The asymmetry, metamorphosis, and origin of flat-fishes.*Phil. Trans. R.Soc. Lond. B.*211, 75-129 (1923).
29. Larson, R. L. Latest pulse of Earth: Evidence for a mid-Cretaceous superplume. *Geology*19, 547-550 (1991).
30. Li, C., Betancur-R, R., Smith, WL &Ortí, G. Monophyly and interrelationships of Snook and Barramundi (Centropomidaesensu Greenwood) and five new markers for fish phylogenetics. *Mol. Phylogen. Evol.*60, 463-471 (2011).

31. Li, S. Z. and Wang, H.M. Fauna Sinica: Ostichthyes Tleuronectiformes. Science press, Beijing,1995.
32. Li, W., Molecular Evolution. *Sinauer Associates Inc publication*, 1997.
33. Liang, W.C. et al. Probable high prevalence of limb-girdle muscular dystrophy type 2D in Taiwan. *J.Neurol.Sci.*362, 304-308 (2016).
34. Lin, Q. et al. The seahorse genome and the evolution of its specialized morphology.*Nature*540, 395-399 (2016).
35. Lindblad-Toh, K.A high-resolution map of human evolutionary constraint using 29mammals. *Nature*478, 476-482 (2011).
36. Minegishi, K. et al. AWnt5 Activity Asymmetry and Intercellular Signaling via PCP Proteins Polarize Node Cells for Left-Right Symmetry Breaking. *Dev Cell.*40, 439-452 (2017).
37. Monies, D., Alhindi, H. N., Almuhaizea, M. A., et al. A first-line diagnostic assay for limb-girdle muscular dystrophy andother myopathies. *Hum. Genomics*10, 32 (2016).
38. Montague, T.G., Gagnon, J.A. & Schier A.F.Conserved regulation of Nodal-mediated left-right patterning in zebrafish and mouse. *Development*145, 1-9 (2018).
39. Orcutt, H.G. The life history of the starry flounder, Platichthysstellatus(Pallas). *California Department of Natural Resouces Division of Fish and Game Fish Bulletin*78, 1-64 (1950).
40. Ostberg, C.O., Chase, D.M.& Hauser, L. Hybridization between Yellowstone Cutthroat Trout and Rainbow Trout Alters the Expression of Muscle Growth-Related Genes and Their Relationships with Growth Patterns. *Plos one*10, 1-16 (2015).
41. Pavey, S.A. et al. Ecological transcriptomics of lake-type and riverine sockeye salmon (Oncorhynchus nerka). *BMC Ecology*11, 31 (2011).
42. Qiu, Q.et al. The yak genome and adaptation to life at high altitude. *Nat.Genet.*44, 946-949 (2012).
43. Satoh, W., Matsuyama, M., Takemura, H., Aizawa, S. &Shimono, A. Sfrp1, Sfrp2, and Sfrp5 regulate the Wnt/beta-catenin and the planar cell polarity pathways during early trunk formation in mouse. *Genesis*46, 92-103 (2008).
44. Schloesser, R.W. & Fabrizio, M.C. Condition indices as surrogates of energy density and lipid content in juveniles of three fish species.*T.Am.Fish.Soc.*146, 1058-1069 (2017).
45. Schreiber,A.M.Flatfish: An Asymmetric Perspective on Metamorphosis. *Curr. Top. Dev. Biol.*103, 167-188 (2013).
46. Schweickert, A. et al. Vertebrate left-right asymmetry: what can Nodal cascade gene expression patterns tell us?*J. Cardiovasc. Dev. Dis.*5, 1 (2018).
47Secco, D.et al. Spatio-Temporal Transcript Profiling of Rice Roots and Shoots in Response to Phosphate Starvation and Recovery. *Plant Cell*25, 4285-4304 (2013).

48. Seton, M., Gaina,C., Müller, R.D.& Heine, C. Mid-Cretaceous seafloor spreading pulse: Fact or fiction?. *Geology*37, 687-690 (2009).

49. Shao, C.W., Bao, B. L., Xie, Z. Y. et al. The genome and transcriptome of Japanese flounder provide insights into flatfish asymmetry. *Nat. Genet.*49, 119-124 (2017). 50. Shi, W. et al. Flatfish monophyly refereed by the relationship of Psettodes in Carangimorphariae. *Bmc Genomics*19, 1-11 (2018).

51. Small, C.M. et al. The genome of the Gulf pipefish enables understanding of evolutionary innovations. *Genome Biol.*17, 258 (2016).

52. Suzuki, T. et al. Metamorphic pitx2 expression in the left habenula correlated with lateralization of eye-sidedness in flounder. *Dev.Growth & Differ.*51, 797-808 (2009).

53. Takezaki, N., Rzhetsky, A. & Nei, M. Phylogenetic Test of the Molecular Clock and Linearized Trees. *Mol. Biol. Evol.*12, 823-833 (1995).

54. Tang, H.B.et al. Synteny and Collinearity in Plant Genomes. *Science*320, 486-488 (2008). 55. Vermot, J. et al. Retinoic acid controls the bilateral symmetry of somite formation in the mouse embryo. *Science*308, 563-566 (2005).

56. Vilhais-Neto, G.C. et al. Rere controls retinoic acid signalling and somite bilateral symmetry. *Nature*463, 953-957 (2010).

57. Wander, R.C.& Patton, B.D.Lipids and fatty acids of three species of northeast pacific finfish harvested in summer. *J.Food. Cpmpos. Anal.*4, 128-135 (1991).

58. Watanabe, H. et al. Nodalsignalling determines biradial asymmetry in Hydra. *Nature*515, 112-115 (2014).

59. Wu,J.&Mlodzik,M.Wnt/PCP Instructions for Cilia in Left-Right Asymmetry.*Dev. Cell.*40, 423-424 (2017).

60. Xie, Z., Hou, Y., Yu, M., et al. Clinical and genetic spectrum of sarcoglycanopathies in a large cohort of Chinese patients. *Orphanet J. Rare Dis.*14, 1-13 (2019).

61. Yang, Z. PAML4: Phylogenetic Analysis by Maximum Likelihood. *Mol. Biol. Evol.*24, 1586-1591 (2007).

62. Yasuhara, R., Yuasa, T.andWilliams, J.A. Wnt/-Catenin and retinoic acid receptor signaling pathways interact to regulate chondrocyte function and matrix turnover. *J Biol. Chem.*285, 317-327 (2010).

63. Yoshioka, H. et al.Pitx2, a bicoid-type homeobox gene, is involved in a lefty-signaling pathway in determination of left-right asymmetry. *Cell*94, 299-305 (1998).

64. Zhang, G. et al. The Apostasia genome and the evolution of orchids.*Nature* 549, 379-383 (2017).

65. Zhao, M. et al. TMAVA, a Metabolite of Intestinal Microbes, Is Increased in Plasma From Patients With Liver Steatosis, Inhibits g-Butyrobetaine Hydroxylase, and Exacerbates Fatty Liver in Mice. *Gastroenterology*158, 2266-2281 (2020).

Decision Letter, first revision:

13th Jan 2021

Dear Yongxin,

Thank you for submitting your revised manuscript "Large-scale flatfish genome sequencing provides insights into non-monophyletic origin of their specialized body plan" (NG-A55146R). It has now been seen by the original referees and their comments are below. The reviewers find that the paper has improved in revision, and therefore we will be happy in principle to publish it in Nature Genetics, pending minor revisions to satisfy the referees' final requests and to comply with our editorial and formatting guidelines.

** Note that I will send you a checklist detailing these editorial and formatting requirements in about a week. Please do not finalize your revisions or upload the final materials until you receive this additional information.**

In recognition of the time and expertise our reviewers provide to Nature Genetics's editorial process, we would like to formally acknowledge their contribution to the external peer review of your manuscript entitled "Large-scale flatfish genome sequencing provides insights into non-monophyletic origin of their specialized body plan". For those reviewers who give their assent, we will be publishing their names alongside the published article.

While we prepare these instructions, we encourage the Corresponding Author to begin to review and collect the following:

-- Confirmation from all authors that the manuscript correctly states their names, institutional affiliations, funding IDs, consortium membership and roles, author or collaborator status, and author contributions.

-- Declarations of any financial and non-financial competing interests from any author. For the sake of transparency and to help readers form their own judgment of potential bias, the Nature Research Journals require authors to declare any financial and non-financial competing interests in relation to the work described in the submitted manuscript. This declaration must be complete, including author initials, in the final manuscript text.

If you have any questions as you begin to prepare your submission please feel free to contact our Editorial offices at genetics@us.nature.com. We are happy to assist you.

Thank you again for your interest in Nature Genetics.

natureresearch

Sincerely,

Tiago

Tiago Faial, PhD
Senior Editor
Nature Genetics
https://orcid.org/0000-0003-0864-1200

Reviewer #1 (Remarks to the Author):

I went through the revised paper carefully. My major concern regarded the cause or the origin of identified genes involved in the unique body plan, and genomic questions such as the out-group species selected for comparative analysis that raised some important questions, most of which are addressed by the authors.
I just have a few small questions. In line 352-354, previously a study has shown that the WNT and RA signaling pathways are possibly involved in the metamorphosis in flatfish respectively. So, one may not say "for the first time" because the cross-talk between WNT and RA are not verified in this study. Besides, please follow the gene nomenclature of teleost species with lowercase italic style. Anyway, I think the results' interpretation is enough at the genomic level for this paper.

Reviewer #3 (Remarks to the Author):

The authors have addressed the major points from all three reviewers. The main issues I had were overinterpreting their results. This is much better now.

ORCID

Nature Genetics is committed to improving transparency in authorship. As part of our efforts in this direction, we are now requesting that all authors identified as 'corresponding author' create and link their Open Researcher and Contributor Identifier (ORCID) with their account on the Manuscript Tracking System (MTS) prior to acceptance. ORCID helps the scientific community achieve unambiguous attribution of all scholarly contributions. For more information please visit http://www.springernature.com/orcid

For all corresponding authors listed on the manuscript, please follow the instructions in the link below to link your ORCID to your account on our MTS before submitting the final version of the manuscript. If you do not yet have an ORCID you will be able to create one in minutes.
https://www.springernature.com/gp/researchers/orcid/orcid-for-nature-research

natureresearch

IMPORTANT: All authors identified as ‘corresponding author’ on the manuscript must follow these instructions. Non-corresponding authors do not have to link their ORCIDs but are encouraged to do so. Please note that it will not be possible to add/modify ORCIDs at proof. Thus, if they wish to have their ORCID added to the paper they must also follow the above procedure prior to acceptance.

To support ORCID's aims, we only allow a single ORCID identifier to be attached to one account. If you have any issues attaching an ORCID identifier to your MTS account, please contact the Platform Support Helpdesk.

Author Rebuttal, first revision:

Reviewer #1:

I went through the revised paper carefully. My major concern regarded the cause or the origin of identified genes involved in the unique body plan, and genomic questions such as the out-group species selected for comparative analysis that raised some important questions, most of which are addressed by the authors.
I just have a few small questions. In line 352-354, previously a study has shown that the WNT and RA signaling pathways are possibly involved in the metamorphosis in flatfish respectively. So, one may not say “for the first time” because the cross-talk between WNT and RA are not verified in this study. Besides, please follow the gene nomenclature of teleost species with lowercase italic style. Anyway, I think the results' interpretation is enough at the genomic level for this paper

Response: Thank you for your valuable suggestions.

1) We have deleted the phrase “for the first time” in line 354 according to your suggestion and revised it into “Taken together, our analyses provide gene evolution and expression evidence for the possible involvement of WNT combined with RA signal pathways in shaping the asymmetric body plan in flatfishes (Fig. 4f), though the exact role of these RA and WNT genes in the body plan asymmetry still awaits further investigations”.
2) We have revised and followed the gene nomenclature of teleost species with lowercase italic style throughout the manuscript as requested.

Final Decision Letter:

5th Mar 2021

Dear Yongxin,

I am delighted to say that your manuscript "Large-scale sequencing of flatfish genomes provides insights into the polyphyletic origin of their specialized body plan" has been accepted for publication in an upcoming issue of Nature Genetics.

Prior to setting your manuscript, we may make minor changes to enhance the lucidity of the text and with reference to our house style. We therefore ask that you examine the proofs most carefully to ensure that we have not inadvertently altered the sense of your text in any way.

Once your manuscript is typeset and you have completed the appropriate grant of rights, you will receive a link to your electronic proof via email with a request to make any corrections within 48 hours. If, when you receive your proof, you cannot meet this deadline, please inform us at rjsproduction@springernature.com immediately.

Your paper will be published online after we receive your corrections and will appear in print in the next available issue. You can find out your date of online publication by contacting the Nature Press Office (press@nature.com) after sending your e-proof corrections. Now is the time to inform your Public Relations or Press Office about your paper, as they might be interested in promoting its publication. This will allow them time to prepare an accurate and satisfactory press release. Include your manuscript tracking number (NG-A55146R1) and the name of the journal, which they will need when they contact our Press Office.

Before your paper is published online, we shall be distributing a press release to news organizations worldwide, which may very well include details of your work. We are happy for your institution or funding agency to prepare its own press release, but it must mention the embargo date and Nature Genetics. Our Press Office may contact you closer to the time of publication, but if you or your Press Office have any enquiries in the meantime, please contact press@nature.com.

Acceptance is conditional on the data in the manuscript not being published elsewhere, or announced in the print or electronic media, until the embargo/publication date. These restrictions are not intended to deter you from presenting your data at academic meetings and conferences, but any enquiries from the media about papers not yet scheduled for publication should be referred to us.

Nature Genetics is a Transformative open access journal. In approximately 10 business days you will receive an email with a link to choose the appropriate publishing options for your paper and our Author Services team will be in touch regarding any additional information that may be required.

Please note that Nature Research offers an immediate open access option only for papers that were first submitted after 1 January, 2021.

You will not receive your proofs until the publishing agreement has been received through our system.

If you have any questions about our publishing options, costs, Open Access requirements, or our legal forms, please contact ASJournals@springernature.com

In the event that you choose to publish under the subscription model, Nature Research allows authors to self-archive the accepted manuscript (the version post-peer review, but prior to copy-editing and typesetting) on their own personal website and/or in an institutional or funder repository where it can be made publicly accessible 6 months after first publication, in accordance with our self-archiving

policy. Please review our self-archving policy for more information.

Several funders require deposition the accepted manuscript (AM) to PubMed Central or Europe PubMed Central. To enable compliance with these requirements, Nature Research therefore offers a free manuscript deposition service for original research papers supported by a number of PMC/EPMC participating funders. If you do not choose to publish immediate open access, we can deposit the accepted manuscript in PMC/Europe PMC on your behalf, if you authorise us to do so.

If you have posted a preprint on any preprint server, please ensure that the preprint details are updated with a publication reference, including the DOI and a URL to the published version of the article on the journal website.

To assist our authors in disseminating their research to the broader community, our SharedIt initiative provides you with a unique shareable link that will allow anyone (with or without a subscription) to read the published article. Recipients of the link with a subscription will also be able to download and print the PDF.

As soon as your article is published, you will receive an automated email with your shareable link.

You can now use a single sign-on for all your accounts, view the status of all your manuscript submissions and reviews, access usage statistics for your published articles and download a record of your refereeing activity for the Nature journals.

An online order form for reprints of your paper is available at https://www.nature.com/reprints/author-reprints.html. Please let your coauthors and your institutions' public affairs office know that they are also welcome to order reprints by this method.

Sincerely,

Tiago

Tiago Faial, PhD
Senior Editor
Nature Genetics
https://orcid.org/0000-0003-0864-1200

Click here if you would like to recommend Nature Genetics to your librarian
http://www.nature.com/subscriptions/recommend.html#forms

** Visit the Springer Nature Editorial and Publishing website at www.springernature.com/editorial-and-publishing-jobs for more information

about our career opportunities. If you have any questions please click here.**
